# Chemotherapeutic agents and leucine deprivation induce codon-biased aberrant protein production in cancer

**DOI:** 10.1093/nar/gkae1110

**Published:** 2024-11-26

**Authors:** Adva Kochavi, Remco Nagel, Pierre-Rene Körner, Onno B Bleijerveld, Chun-Pu Lin, Zowi Huinen, Yuval Malka, Natalie Proost, Marieke van de Ven, Xiaodong Feng, Jasmine Montenegro Navarro, Abhijeet Pataskar, Daniel S Peeper, Julien Champagne, Reuven Agami

**Affiliations:** Division of Oncogenomics, Oncode Institute, The Netherlands Cancer Institute, Plesmanlaan 121, 1066CX, Amsterdam, the Netherlands; Division of Oncogenomics, Oncode Institute, The Netherlands Cancer Institute, Plesmanlaan 121, 1066CX, Amsterdam, the Netherlands; Division of Oncogenomics, Oncode Institute, The Netherlands Cancer Institute, Plesmanlaan 121, 1066CX, Amsterdam, the Netherlands; NKI Proteomics Facility, The Netherlands Cancer Institute, Plesmanlaan 121, 1066CX, Amsterdam, The Netherlands; Division of Molecular Oncology and Immunology, Oncode Institute, The Netherlands Cancer Institute, Plesmanlaan 121, 1066CX, Amsterdam, The Netherlands; Division of Molecular Oncology and Immunology, Oncode Institute, The Netherlands Cancer Institute, Plesmanlaan 121, 1066CX, Amsterdam, The Netherlands; Division of Oncogenomics, Oncode Institute, The Netherlands Cancer Institute, Plesmanlaan 121, 1066CX, Amsterdam, the Netherlands; Preclinical Intervention Unit and Pharmacology Unit of the Mouse Clinic for Cancer and Ageing (MCCA), The Netherlands Cancer Institute, Plesmanlaan 121, 1066CX, Amsterdam, The Netherlands; Preclinical Intervention Unit and Pharmacology Unit of the Mouse Clinic for Cancer and Ageing (MCCA), The Netherlands Cancer Institute, Plesmanlaan 121, 1066CX, Amsterdam, The Netherlands; Division of Oncogenomics, Oncode Institute, The Netherlands Cancer Institute, Plesmanlaan 121, 1066CX, Amsterdam, the Netherlands; Division of Oncogenomics, Oncode Institute, The Netherlands Cancer Institute, Plesmanlaan 121, 1066CX, Amsterdam, the Netherlands; Division of Oncogenomics, Oncode Institute, The Netherlands Cancer Institute, Plesmanlaan 121, 1066CX, Amsterdam, the Netherlands; Division of Molecular Oncology and Immunology, Oncode Institute, The Netherlands Cancer Institute, Plesmanlaan 121, 1066CX, Amsterdam, The Netherlands; Division of Oncogenomics, Oncode Institute, The Netherlands Cancer Institute, Plesmanlaan 121, 1066CX, Amsterdam, the Netherlands; Division of Oncogenomics, Oncode Institute, The Netherlands Cancer Institute, Plesmanlaan 121, 1066CX, Amsterdam, the Netherlands; Erasmus MC, Rotterdam University, Dr. Molewaterplein 40, 3015GD, Rotterdam, the Netherlands

## Abstract

Messenger RNA (mRNA) translation is a tightly controlled process frequently deregulated in cancer. Key to this deregulation are transfer RNAs (tRNAs), whose expression, processing and post-transcriptional modifications are often altered in cancer to support cellular transformation. In conditions of limiting levels of amino acids, this deregulated control of protein synthesis leads to aberrant protein production in the form of ribosomal frameshifting or misincorporation of non-cognate amino acids. Here, we studied leucine, an essential amino acid coded by six different codons. Surprisingly, we found that leucine deprivation leads to ribosomal stalling and aberrant protein production in various cancer cell types, predominantly at one codon, UUA. Similar effects were observed after treatment with chemotherapeutic agents, implying a shared mechanism controlling the downstream effects on mRNA translation. In both conditions, a limitation in the availability of tRNA^Leu^(UAA) for protein production was shown to be the cause for this dominant effect on UUA codons. The induced aberrant proteins can be processed and immune-presented as neoepitopes and can direct T-cell killing. Altogether, we uncovered a novel mode of interplay between DNA damage, regulation of tRNA availability for mRNA translation and aberrant protein production in cancer that could be exploited for anti-cancer therapy.

## Graphical Abstract

**Graphical Abstract:**
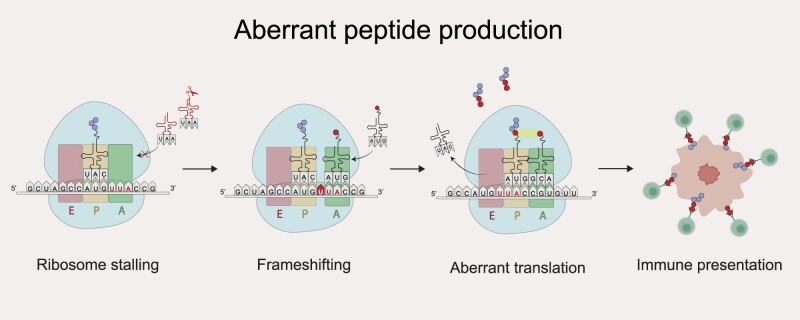

## Introduction

Even though cancer is considered a genetic disease, evidence indicates that global deregulation of protein production contributes to malignant transformation and cancer progression (1,2). This deregulated protein production in cancer is facilitated by oncogene-induced alterations in signaling to ribosomes, increased expression of ribosomal subunits, abnormal codon-biased mRNA translation and changes in the levels or modifications of transfer RNAs (tRNAs) (3–7).

Accumulating evidence shows that tRNAs are highly modified, with an average of around 12 modifications within a limited length of merely 80 nucleotides (8). These modifications vary significantly between tissue types, tRNA isoforms and cellular conditions, whereby protein synthesis is influenced (9). Not surprisingly, several studies indicate that tumor cells hijack machineries for tRNA modifications to drive oncogenic mRNA translation. In particular, specific codon-biased mRNA translation programs are achieved by the presence or absence of modifications in the anticodon loop, which ensures high fidelity or efficiency in mRNA decoding (5). Melanomas driven by the B-Raf Proto-Oncogene^V600E^ (BRAF) oncogene, for example, were shown to depend critically on tRNA modifications at the wobble position for the oncogenic rewiring of mRNA translational programs (10). In addition to alterations in modifications, deregulation of tRNA levels and stability have been observed in tumor samples as well. In breast cancer, upregulated expression of specific tRNAs for glutamic acid drives a metastatic pathway, and leucyl-tRNA synthetase 1 (LARS1) was shown to function as a tumor suppressor by promoting codon-specific translation of growth suppressive genes (11,12). Another interesting example is the activity of the tRNA-cleaving enzyme Schlafen 11 (SLFN11), which is an important determinant of the response to DNA damaging agents (13). SLFN11 is activated upon DNA-damage, targeting type II tRNAs with high specificity toward tRNA^leu^(UAA) (14,15). This activation ultimately results in reduced translation efficiency of transcripts enriched with the corresponding UUA leucine codon (14,16,17).

Thus, in addition to contributing to carcinogenesis, deregulated mRNA translation can become a liability for tumor cells in stress conditions. Recent studies have indicated that the continuous demand of cancer cells for *de novo* protein synthesis has been shown to form an Achilles heel for tumor cells, which could be exploited for therapy (18). Due to the impaired capacity to regulate mRNA translation, shortages in the essential amino acids can lead to cancer-specific aberrant protein production and the widespread formation of inducible neoepitopes (19–22).

In this work, we studied the impact of shortages of the essential amino acid leucine on mRNA translation in cancer. We observed an unexpected predominant stalling of ribosomes on only one out of six codons for leucine, UUA. Interestingly, similar effects on the same codon could be achieved after treatment with DNA-damaging agents, as a result of SLFN11 activation. We show that this dominant effect on UUA codons in both conditions results from limiting the availability of tRNA^leu^(UAA) for mRNA translation. Furthermore, we show that both leucine deprivation and treatment with chemotherapeutic agents lead to aberrant protein production mainly from UUA codons. These faulty protein products are immune presented and can serve as targets for T-cell recognition.

## Materials and methods

### Cells and reagents

PC3, MD55A3 and DU145 cells were cultured in Roswell Park Memorial Institute 1640 Medium (RPMI 1640, Gibco) supplemented with 10% fetal bovine serum (Gibco) and 100 U/ml penicillin–streptomycin (Gibco). HEK293T, A375, SNB-19 A549, RPE-1, BJ fibroblasts and MDA-MB-231 cells were cultured in Dulbecco’s modified Eagle’s medium (DMEM; Gibco), supplemented with 10% fetal bovine serum and 100 U/ml penicillin–streptomycin. All cell lines were maintained in a humidified atmosphere containing 5% CO_2_ at 37 °C and were regularly tested by polymerasechain reaction (PCR) analysis and were found negative for mycoplasma contamination. Leucine-free medium was prepared by adding glucose (Gibco), sodium pyruvate (Sigma) and all amino acids except for L-leucine to RPMI 1640 Medium without amino acids (US Biologicals). MG-132 (Selleckchem) and etoposide (Selleckchem) were dissolved in dimethylsufloxide (DMSO) and both used at a final concentration of 10 μM, unless stated otherwise. Cisplatin (Accord-Healthcare) was dissolved in water at concentration of 1 mg/ml, and hydroxyurea (Sigma) was dissolved in DMSO at a final concentration of 500 mM. Polyethylenimine (PEI, Polysciences) was dissolved in water at concentration of 1 mg/ml. All treatments were given for 48 h before downstream analyses.

### Generation of constructs

A gBlock (Integrated DNA Technologies) was synthesized for split superfolderGFP (sfGFP)-(β1–10)-spacer-(β11) (Supplementary Table S1). This sequence was ligated in the XbaI and NotI sites in the pCDH-Blast vector using T4 DNA ligase (Thermo Scientific) for 1 h at room temperature. The ligation products were subsequently transformed to DH5α competent cells. Next, the produced vector was used as a template for PCR to generate the leucine reporters. An N-terminal V5-tag and individual leucine codons before β-sheet 11 were added to the sequence by PCR (primers listed in Supplementary Table S1). PCR products were purified and digested with NheI and NotI restriction enzymes for 1 h at 37°C. After digestion, the DNA was purified and ligated into the XbaI and NotI sites of the pCDH-Blast plasmid using T4 DNA ligase (Thermo Scientific) for 1 h at room temperature. The ligation products were subsequently transformed to DH5α competent cells. SLFN11 sequence was digested from pcDNA-SLFN11 plasmid using NheI and NotI restriction enzymes. The digested sequence product was run on a 1% agarose (Merck) gel and was purified using the ‘Wizard SV Gel and PCR clean-up system’ (Promega). Then, the sequence was ligated in the XbaI and NotI sites in the pCDH-puro vector using T4 DNA ligase (Thermo Scientific) for 2 h at room temperature. All resulting plasmids were sequence verified by Sanger sequencing (Macrogen).

pLentiCRISPRv2-puro plasmid was digested using FastDigest Esp3I and dephosphorylated with FastAP enzymes (both from Thermo Scientific). Then, the digested vector product was run on a 1% agarose (Merck) gel and was purified using the ‘Wizard SV Gel and PCR clean-up system’ (Promega). Oligonucleotides targeting LARS1 or SLFN11 (Supplementary Table S1) were annealed and phosphorylated using T4 PNK. The digested vector and the annealed oligos were ligated using T4 DNA ligase (Thermo Scientific) for 1 h at room temperature. The ligation products were transformed to subcloning efficiency DH5α competent cells (Invitrogen). All resulting plasmids were sequence verified by Sanger sequencing.

The pLKO.1-puro vector was digested with EcoRI and AgeI restriction enzymes (both from New England Biolabs) and dephosphorylated with FastAP enzyme (Thermo Scientific). The digested vector product was run on a 1% agarose (Merck) gel and was purified using the ‘Wizard SV Gel and PCR clean-up system’ (Promega). Oligonucleotides coding for tRNA-Leu-CAA-6–1 or tRNA-Leu-TAA-1–1 were annealed and phosphorylated (Supplementary Table S1). Next, a ligation reaction was performed with the annealed oligos and the digested vector using T4 DNA ligase (Thermo Scientific) for 1 h at room temperature. The ligation products were subsequently transformed to DH5α competent cells. All resulting plasmids were sequence verified by Sanger sequencing.

pLKO.1-puro vectors for expression of tRNA-Leu-CAG-1–7, tRNA-Leu-CAG-1–7-to-TAA and tRNA-TAA-3–1 were a kind gift from Heidi Greulich (23).

### Lentiviral production and transduction

For the production of lentiviruses, HEK293T cells were seeded in tissue culture dishes and transfected the next day. For each transfection, 10 μg of the lentiviral backbone to be packaged, 5 μg of pMDL RRE, 3.5 μg pVSV-G and 2.5 μg of pRSV-REV plasmids were mixed, Next, 63 μl of a 1 mg/ml PEI solution was added. After mixing, the solution was left at room temperature for 15 min, after which it was added to the HEK293T cells. The next day, the medium was replaced by fresh medium. The lentivirus-containing supernatants were collected 48 and 72 h after transfection, and snap-frozen in liquid nitrogen. Lentivirus-containing supernatants were supplemented with 8 μg/ml polybrene (Sigma) before being used for transduction of the target cells. One day after transduction, transduced cells were selected by the addition of 5 μg/ml blasticidin (Invivogen), 2 μg/ml puromycin (Adipogen) or 50–300 ng/ml hygromycin B (Gibco) to the medium.

### Ribosome profiling

#### Sample preparation for ribosome profiling

The generation of ribosome-protected fragment (RPF) libraries was done as previously described (24). Total RNA was isolated using Trizol reagent (Invitrogen), according to manufacturer’s instructions. Rapid gel extraction was used, followed by overnight precipitation at −20°C for RNA extraction from the polyacrylamide gels. Ribosomal RNA (rRNA) depletion was not performed. Prepared RPF libraries were analyzed on a 2100 Bioanalyzer using a 7500 chip (Agilent, Santa Clara, CA) and quantified by quantitative polymerase chain reaction (qPCR, cat.no. 07 960 140 001, Roche, Switzerland) and subsequently diluted to a 10 nM concentration. Next, the libraries were pooled and loaded with 1.5 pM on a NextSeq550Dx system (RUO mode) and sequenced with 75-base single-read using a 75 Cycles High Output Kit v2.5 (Illumina Inc., San Diego).

#### Data analysis

Adaptors were removed from FASTQ files using cutadapt (25) with parameters (-quality-base = 33 -O 7 -e 0.15 -m 20 -q 5). rRNA and tRNA contaminants were removed by aligning FASTQ reads with Bowtie2 (26) and parameters (-seed 42 -p1 -local) against a reference library (rRNA reference was obtained from GENCODE v19 (27) : rRNA, MT_RNA, rRNA_pseudogene and tRNA reference data were obtained from GtRNAdb) (28).

Preprocessed FASTQ files were then aligned with TopHat2 (29) and Bowtie2 against GRCh37/hg19 and GENCODE v19/BASIC transcripts with Ensembl coordinates using the parameters (seed 41 -n 2-m 1 -novel-juncs – no-novel-indels –no-coverage-search –segment-length 25). Aligned reads were then filtered for minimum mapping quality of 10. Read depth per sample is displayed in Supplementary Table S2.

Quality control of frames and periodicity of RPFs were performed using the RiboWaltz (30) package (version 2.0). For this analysis transcript aligned files were used and no additional filters were added. For visualization the confidence level was set to 99.

For subsequence analysis, the frequency of codon occupancy of RPFs was compared between two tested conditions (e.g. leucine depletion versus WT), as previously described (20) with a minimum readcount of 100 per gene in all conditions. RPF density analysis was performed by comparison of normalized 5′-RPF densities per codon between two conditions, as described in (31) with a minimum readcount of 25 per gene.

To determine the potential of cloning bias within our ribosome profiling we utilized the RUST pipeline (32). As suggested by the RUST pipeline the A-site was set at position 17 and readlength offset was between 28 and 30 nucleotides.

#### Western blotting

Proteins of interest were visualized by sodium dodecyl sulfate-polyacrylamide gel electrophoresis (SDS-PAGE) and western blotting. Cell lysates were prepared by straight lysing in 1 × Laemmli buffer. Proteins were separated by SDS-PAGE gels and transferred 22-μm pore size nitrocellulose membranes (Santa Cruz). Stainings were performed using V5 Tag antibodies (Thermo Fisher Scientific, 1:1000), Tubulin (Santa Cruz, 1:10 000), LARS1 (Cell Signaling. 1:1000), HSP90αβ (Santa Cruz, 1:1000), RPA32-S33 (Bethyl, 1:1000) or SLFN11 (Santa Cruz, 1:1000) antibodies. IRDye 680RD donkey anti-mouse (LI-COR, 926–68 072, 1:10 000), IRDye 800CW goat anti-rat (LI-COR, 926–32 219, 1:10 000) and IRDye 800CW goat anti-rabbit (LI-COR, 926–32 211, 1:10 000) were used as secondary antibodies. Visualization was performed by use of an Odyssey infrared scanning device (LI-COR).

#### Flow cytometry analyses

Cells expressing the split sfGFP reporters were seeded and mock-treated or treated with the indicated treatments the next day. Forty-eight hours after the start of treatment, cells were collected by trypsinization and centrifugation. Next, the cells were analyzed on an Attune NxT machine (Thermo Fisher Scientific). Obtained data were analyzed using FlowJo V10 software (FlowJo).

#### Amino acid mass spectrometry

Cells were washed with cold PBS and lysed with lysis buffer composed of methanol/acetonitrile/H_2_O (2:2:1). The lysates were collected and centrifuged at 16 000 *g* (4°C) for 15 min and the supernatant was transferred to a new tube for liquid chromotography-mass spectometry (LC-MS) analysis.

LC-MS analysis was performed on an Exactive mass spectrometer (Thermo Fisher Scientific) coupled to a Dionex Ultimate 3000 autosampler and pump (Thermo Fisher Scientific). Metabolites were separated using a Sequant ZIC-pHILIC column (2.1 × 150 mm, 5 μm, guard column 2.1 × 20 mm, 5 μm; Merck) using a linear gradient of acetonitrile (A) and eluent B (20 mM (NH_4_)_2_CO_3_, 0.1% NH_4_OH in ULC/MS grade water (Biosolve)), with a flow rate of 150 μl/min. The MS operated in polarity-switching mode with spray voltages of 4.5 kV and −3.5 kV. Metabolites were identified on the basis of exact mass within 5 ppm and further validated by concordance with retention times of standards. Quantification was based on peak area using LCquan software (Thermo Fisher Scientific).

### Analyses of tRNA expression

#### NCI-60 cell line panel small RNA-seq analysis for tRNA expression

Small non-coding RNA sequencing reads from the NCI-60 panel were obtained from the sequence read archive (accession number PRJNA390643) (33). The tRNA genome reference files in fasta format (hg19) was retrieved from GtRNAdb (28). Alignment of reads was done with bowtie2 (26) using the parameters (-N 1 –very-sensitive). Aligned reads per tRNA were counted from SAMfiles using a custom script. Obtained tRNA reads per sample were then normalized using the TMM method from the edgeR package (34).

#### qPCR

For qPCR analysis of levels of expression of the sfGFP reporter, total RNA was isolated from UUA+1 and UUG+1 expressing MDA-MB-231 and RPE-1. RNA extraction was performed isolated using Trizol reagent (Invitrogen), according to manufacturer’s instructions. Samples were treated with Turbo DNase (Thermo Fisher Scientific) and RNA was purified using the Zymo RNA Clean and Concentrator-5 kit (Zymo Research). RNA was converted to complementary DNA (cDNA) by use of High-Capacity cDNA Reverse Transcription Kit, according to manufacturer’s instructions, using random hexamers for priming. qPCRs to detect levels of expression of the split sfGFP reporter and GAPDH were performed using the primers in Supplementary Table S3. qPCRs were performed using the SensiFAST SYBR Lo-ROX kit (Bioline) on a Quantstudio 5 machine (Applied Biosystems). Expression levels of the reporter were normalized to GAPDH (^Δ^Ct).

For qPCR analysis of exogenously expressed tRNA levels, total RNA was isolated using Trizol reagent (Invitrogen), according to manufacturer’s instructions. RNA was converted into cDNA by use of the rtStar tRNA pretreatment and first-strand cDNA synthesis kit (Arraystar). qPCRs to determine tRNA expression levels were performed using the primers listed in Supplementary Table S3. The qPCRs were performed using the SensiFAST SYBR No-ROX kit (Bioline) on a Quantstudio 5 machine (Applied Biosystems). Data were analyzed by use of the ^ΔΔ^Ct method.

For qPCR analysis of tRNA aminoacylation levels, previously described methods were used (11,31,35,36). In short, total RNA was isolated using Trizol reagent (Invitrogen), according to manufacturer’s instructions. The samples were treated with Turbo DNase (Thermo Fisher Scientific), and small RNA fraction containing tRNAs (≤200 nt) was retained using a Zymo RNA Clean and Concentrator-5 kit (Zymo Research). Samples were split in two: deacylated and non-deacylated. The sample used for deacylation was incubated in 50 mM Tris–HCl (pH 9.0) at 37°C for 30 min and recovered using a Zymo RNA Clean and Concentrator-5 kit (Zymo Research). Next, both deacylated and non-deacylated tRNAs were then ligated to an annealed adapter set complementary to the 3′ NCCA tRNA overhang, using T4 RNA ligase 2 (New England Biolabs) for 2 h at room temperature. Then, annealed reverse transcription (RT) template set was ligated using T4 DNA ligase (New England Biolabs) for 30 min at room temperature. The ligated tRNA samples were converted into cDNA using Maxima H Minus Reverse Transcriptase cDNA synthesis kit (Life Technologies). qPCRs to determine tRNA levels were performed using the primers listed in Supplementary Table S3. The qPCRs were performed using the SensiFAST SYBR No-ROX kit (Bioline) on a Quantstudio 5 machine (Applied Biosystems). Relative aminoacylation levels were calculated by qRT-PCR using the levels of each detected tRNA in the deacylated and non-deacylated samples.

#### RNA sequencing

MDA-MB-231 cells were either treated with control medium or leucine-less medium for 48 h. Next, the cells were harvested in RLT buffer (Qiagen) and total RNA was isolated using the RNeasy Mini Kit (Qiagen), including an on column DNase digestion, according to the manufacture’s instructions. Quality and quantity of the total RNA was assessed by the 2100 Bioanalyzer using ‘Agilent RNA 6000 Nano’ (Agilent Technologies). Total RNA samples having RIN > 8 were subjected to library generation using the TruSeq stranded mRNA library preparation kit, according to the manufacturer’s instructions (Illumina) with incorporation of xGen UDI-UMI adapters (Integrated DNA Technologies). Stranded mRNA libraries were analyzed on a 2100 Bioanalyzer instrument following the manufacturer’s protocol ‘Agilent DNA 7500 kit’ (Agilent Technologies), diluted to 10 nM and pooled at equimolar ratios into multiplexed sequencing pools for paired end sequencing on a NovaSeq 6000 Illumina sequencing instrument. Paired-end sequencing was performed using 54 cycles for Read 1, 19 cycles for Read i7, 10 cycles for Read i5 and 54 cycles for Read 2, using the NovaSeq6000 Reagent Kit v1.5 (100 cycles, Illumina). On average 20 M paired reads per sample were generated.

RNA sequencing data was aligned to the human hg19 genome (GRCh37.p13) using STAR (version 2.6.1). File conversions were performed using SAMtools. Readcounts per gene were obtained by HTSeq, couting reads at exons of protein-coding genes. Normalization of library size was performed using DESeq2. Next, the log_2_-transformed ratios of transcript expression (-Leu/Ctrl) were binned for UUA content.

### Proteomics

#### Data generation from proteomics

For proteome-wide screening for aberrant proteins, cells were grown in Petri dishes and given the indicated treatments for 48 h, and subsequently treated with 10 μM of MG132 for 4 h. Cell pellets were lysed in 1x S-Trap lysis buffer according to the manufacturer’s instructions (ProtiFi) using probe sonication. Aliquots comprising 225 μg of protein were reduced and alkylated with 20 mM dithiothreitol (DTT, 15 min at 55°C) and 40 mM iodoacetamide (20 min at room temperature in the dark), respectively. Proteins were digested overnight with trypsin (Sigma-Aldrich; enzyme/substrate ratio 1:10) on S-Trap Mini spin columns (Protifi) according to the manufacturer’s instructions. Peptides were eluted, vacuum dried and stored at −80°C.

2D-LC-MS/MS was achieved as described previously (19), with the following changes. On-line liquid chromatography was performed on a Proxeon nLC1200 system connected to an Orbitrap Fusion Tribrid mass spectrometer (Thermo Scientific). HpH-RP peptide fractions were reconstituted in 2% formic acid, after which 10% of the sample was directly loaded onto the analytical column (ReproSil-Pur 120 C18-AQ, 2.4μm, 75 μm × 500 mm column, packed in-house in fritted Empty Self Pack NanoLC column tubes with integrated emitter tip (CoAnn Technologies LLC). Peptides were eluted in a 140-min gradient containing a 124-min linear increase from 7% to 24% solvent B (solvent A was 0.1% formic acid/water and solvent B was 0.1% formic acid/80% acetonitrile), followed by washout at 90% solvent B. Survey scans of peptide precursors from *m*/*z* = 375–1500 were acquired in the Orbitrap at 120 K with a 4 × 10^5^ ion count target and a maximum injection time of 50 ms. Tandem MS was performed by quadrupole isolation at 1.6 Th. followed by HCD fragmentation with normalized collision energy of 29 and ion trap MS2 fragment detection. The MS2 ion count target was set to 1 × 10^4^ and the max injection time was set to 100 ms. Only precursors with charge state 2–6 were sampled for MS2. Monoisotopic precursor selection was turned on; the dynamic exclusion duration was set to 30s with a 10 ppm tolerance around the selected precursor. The mass spectrometry proteomics data have been deposited to the ProteomeXchange Consortium (http://proteomecentral.proteomexchange.org) via the PRIDE partner repository (37), with the dataset identifier PXD044085.

#### Construction of an *in silico* database for leucine codon-derived frameshifts and truncations

Coding sequences starting from AUG and ending with a canonical stop-codon were obtained from Ensembl GRCh37 / hg19. All transcript sequences containing an in-frame UUA or a CUA codon were included. Frameshifted sequences were generated in the +1 and -1 direction for all individual in-frame UUA or CUA codons. These generated sequences were translated *in silico* until the first occurrence of a stop codon. In the cases that the +1 frameshift at a codon resulted in a stop-codon immediately, the peptide was labelled as truncated. Canonical human proteome sequences were obtained from uniprot (38). Additional decoy and contaminant sequences were added using the philosopher database custom option (39).

#### Database search and filtration

The obtained database in FASTA format was used with the philosopher pipeline (39) to detect peptides from MZML files. In short, MSFragger (40) was used for peptide detection using the parameters; precursor mass lower -20ppm, upper 20ppm, precursor mass tolerance 20ppm, calibrate mass: True, Deisotoping: True, mass offset: False, isotope error: Standard, digestion: Strictly Trypsin, search_enzyme_nocut P, max missed cleavages: 2. Variable modifications included were: 15.99490 M 3, 42.01060 [^ 1, -17.026500 nQnC 1, 0.984016 [F 1. Further parameters used were Min Length:7, Max Length: 50, digest mass range: 500;5000 Daltons, Max Charge: 2, remove precursor range: -1.5,1.5, topN peaks: 300, minimum peaks: 15.

Peptide validation was then performed with PeptideProphet (41) using the parameters; accmass: True, decoyprobs: True, expectScore: True, Glycosylation: False, ICAT: False, masswidth: 5, minimum probability after first pass of a peptide: 0.9, minimum number of NTT in a peptide: 2.

Peptides they were mapped back toward the uniprot proteome to ensure they are not able to originate from other in-frame sequences. The identified peptides were only considered when they were assigned as trans-frame chimeras and truncated proteins. These peptides were filtered for their presence in either both replicates of control treated cells or their presence in both replicates of the treated cells.

#### Integration of protein versus mRNA levels

Protein expression data of MDA-MB-231 was filtered for not matching to contaminants or reverse controls. Protein Intensity values per gene were then linked to the corresponding mRNA readcounts of MDA-MB-231 cells found for this gene. For each gene the longest transcript (based on gencode version 19) with an intact open reading frame (ORF) was taken as canonical transcript and the number of UUA codons for this transcript was added to the gene name.

To allow further calculations all mRNA readcounts were floored to 10. The mean expression of mRNA read counts between replicates per condition and the mean intensity of proteomics samples between replicates per condition were calculated. Ratios were subsequently binned by absence or presence of UUA codons. The translation efficiency ratio per condition was calculated by dividing protein intensity by mRNA readcounts.

### Immunopeptidomics

#### Sample preparation

Untreated and etoposide treated (48 h) DU145 and SNB-19 cell pellets from ∼100 × 10^6^ cells were subjected to HLA purification as previously described (42,43), with slight modifications. In brief, cell pellets were lysed with lysis buffer containing 0.25% sodium deoxycholate, 0.2 mM iodoacetamide, 1 mM EDTA, 0.1 mM phenylmethylsulfonyl fluoride (PMSF), 1% octyl-β-d-glucopyranoside and protease inhibitors cocktail (Merck) in ultra-pure distilled water (Invitrogen), and then incubated on ice for 1 h. The lysates were cleared by centrifugation at 4°C at 21 000 *g* for 45 min. Approximately 200 μl of protein A–sepharose 4B beads (Invitrogen) were taken for each sample, which were washed twice with 1 ml of lysis buffer. After the last washing step, the cleared lysate and 50 μl of pan-HLA-class I antibody (W6/32 purified from HB95 hybridoma cells) were added to the beads. Subsequently, the samples were incubated at 4°C overnight, with continuous mixing. The next day, the beads were washed four times with 1 ml of 150 mM NaCl in 20 mM Tris–HCl, pH 8.0, two times with 1 ml of 400 mM NaCl in 20 mM Tris–HCl, pH 8.0, two times with 1 ml of 150 mM NaCl in 20 mM Tris–HCl pH 8.0, and finally four times with 1 ml of 20 mM Tris–HCl. Approximately 96-well single-use microplates with glass fiber + 25 μm polyethylene support membranes (Agilent) were first washed with 1 ml of acetonitrile (Biosolve) making use of a positive pressure-96 processor (Waters Corporation). Next, it was washed with 1 ml of 0.1% trifluoroacetic acid (TFA, Merck) in H_2_O and 2 ml of 100 mM Tris–HCl, pH 8.0. After that, the washed beads in 20 mM Tris–HCl, pH 8.0 were loaded on the plate, and the plate was washed once with 1 ml of 20 mM Tris–HCl, pH 8.0. Next, a Sep-Pak tC18 100 mg Sorbent 96-well plate (Waters Corporation) was activated. This plate was washed with 1 ml of 80% acetonitrile (ACN) with 0.1% TFA and with 2 mL of 0.1% TFA in H_2_O. Next, the HLA-bound peptides were eluted from the microplate containing the beads into the Sep-Pak plate by applying 500 μl of 1% TFA in H_2_O. The Sep-Pak plate was washed with 2 ml of 0.1% TFA in H_2_O. Peptides were eluted with 500 μl of 28% ACN with 0.1% TFA, dried in a vacuum concentrator and stored at −80°C. Peptides were analyzed by LC-MS/MS on an Orbitrap Exploris 480 Mass spectrometer connected to an Evosep One LC system (Evosep Biotechnology). Prior to LC separation with the Evosep One, peptides were reconstituted in 0.1% formic acid and 50% of the sample was loaded on Evotip Pure™ (Evosep) tips. Peptides were then eluted and separated using the pre-programmed ‘Extended Method’ (88 min gradient) on an EV1137 (Evosep) column with an EV1086 (Evosep) emitter. Nanospray was achieved using the Easy-Spray NG Ion Source (Thermo Scientific) with a liquid junction set-up at 2 kV. On the Exploris 480, data-dependent acquisition was performed as follows. Full scan MS was acquired at resolution 60 000 with MS1 mass range 350–1700 *m/z*, normalized AGC target was set to 100% and maximum injection time was 50 ms. Dynamic exclusion was set to 10 sec. and MS2 spectra were acquired at 15 000 resolution. The top 10 precursors per cycle were HCD fragmented when their charge states were 2–4, whereas the top 5 precursors per cycle were subjected to HCD fragmentation if they were singly charged. MS2 isolation window was 1.1 *m/z*, the normalized collision energy was 30, the normalized AGC target was set to 50% and the maximum injection time was 100 ms. The mass spectrometry immunopeptidomics data have been deposited to the ProteomeXchange Consortium (http://proteomecentral.proteomexchange.org) via the PRIDE partner repository (37) with the dataset identifier PXD044085.

#### Construction of *in silico* database for leucine codon-derived chimeric peptides

Coding sequences starting from an AUG codon and ending on a canonical stop-codon were obtained from Ensembl GRCh37/hg19. Next, all transcripts containing an in-frame UUA or a CUA codon were included. Frameshifted sequences in the +1 direction were generated for each occurrence of in-frame UUA or CUA codon. The generated sequences were translated *in silico* until the first occurring stop codon. For the immunopeptidomics libraries only amino acids in a window 10 amino acids before and 10 amino acids after the UUA or CUA codon were kept. Only chimeric peptides, consisting from a partly in-frame and out-of-frame peptide sequence were considered. Full-length canonical human proteome sequences were obtained from uniprot (38). Additional decoy and contaminant sequences were added using the philosopher’s (version 5.0.0) database custom option (39).

#### Database search and filtration

The acquired data were run against the constructed database using the FragPipe (version 20.0) pipeline with MSFragger (version 3.8) for label-free quantification and philosopher (version 5.0.0) for post-processing of searched results. Peptide sequences were mapped back to the uniprot proteome to ensure they cannot originate from other in-frame sequences. Immunopeptides were only considered when they were assigned as trans-frame chimeras, meaning that they consist of an in-frame as well as an out-of-frame part, and additionaly were detected in at least two out of three replicates. Binding of the identified neoepitopes to the HLA-alleles expressed in the used cell lines was determined using NetMHCpan-4.1 software (44).

#### Measurement of H2-Kb-bound SIINFEKL levels

DU145 and PC3 cells were transduced with lentiviruses produced from pCDH-Hygro-H2-Kb and selected with hygromycin (Invitrogen). Next, the H2-Kb expressing cells were transduced with lentiviruses generated from the pCDH-V5-split sfGFP UUA +1-SIINFEKL or pCDH-V5-split sfGFP UUG +1-SIINFEKL. Transduced cells were selected for using blasticidin (Invivogen).

For the detection of presented H2-Kb-bound SIINFEKL peptides, cells were treated for 48 h. After treatment, the cells were washed with PBS and detached using PBS–EDTA (50 μM). Next, cells were pelleted and washed with PBS/0.5% BSA and incubated with APC anti-mouse H2-Kb-bound to SIINFEKL antibodies (Biolegend, clone 25-D1.16, 141 606; 1:200 in PBS/0.1% BSA) for 30 min on ice, in the dark. The cells were then washed twice with PBS-BSA and analyzed on an Attune NxT machine using Attune Nxt software version 4.2 (Thermo Fisher Scientific). Data were analyzed using FlowJo V10 software (FlowJo).

#### OT-I T cell SIINFEKL recognition assays

OT-I (B6J) mice were originally from The Jackson Laboratory. Mice used for experiments were between 3 and 12 weeks old and of both sexes. All experiments involving animals were performed in accordance with Dutch and European regulations on care and protection of laboratory animals and have been approved by the local animal experiment committee at Netherlands Cancer Institute, DEC NKI (OZP ID 12051). Mice were bred and maintained in accordance with institutional, national and European guidelines for Animal Care and Use.

OT-I T cells were isolated using Dynabeads Untouched Mouse CD8 Cells Kit (Invitrogen) according to the manufacturer’s protocol. T cells were maintained in Roswell Park Memorial Institute 1640 Medium (Gibco) containing 10% fetal bovine serum (Sigma), 50 μM 2-mercaptoethanol (Sigma), 100 U/ml penicillin, 100 μg/ml streptomycin (both Gibco), 100 μg/mL IL-2 (ImmunoTools), 5 μg/ml IL-7 (ImmunoTools) and 10 μg/ml IL-15 (ImmunoTools).

After 48 h of treatment, DU145 and PC3 cells expressing the SIINFEKL reporters were detached using PBS-EDTA and seeded at 100 000 cells per well in a U-shaped 96-well plate. Next, 100 000 OT-I T cells were added to the co-culture and the solution was supplemented with BD Golgiplug (BD Biosciences). The co-culture samples were then incubated for 4 h at 37 °C in a humidified CO_2_ incubator.

Next, the cells were pelleted by centrifugation, blocked with 0.1% PBS-BSA and stained with anti-mouse CD8-VioBlue antibodies (Miltenyi, 130–111-638, 1:100) and Live/Dead Fixable near-IR dead cell stain kit (Invitrogen). Subsequently, the cells were fixed and permeabilized using the eBioscience Foxp3 Transcription Factor Staining Buffer Set (Invitrogen) according to manufacturer’s instructions. Next, the cells were stained with APC-conjugated anti-mouse IFNγ (Miltenyi, 130–109-723 and 130–120-805, 1:100) antibodies. Cells were then washed and analyzed on a BD LSR Fortessa (BD Biosciences). The data were analyzed using FlowJo V10 software (FlowJo).

#### OT-I T cell-mediated killing assay

After 48 h of treatment, the medium of DU145 and PC3 cells expressing the SIINFEKL reporters was replaced by fresh RPMI. Then OT-I cells were added in different ratios. The co-cultures were left for 24 h at 37 °C in a humidified CO_2_ incubator. After the co-culture, the cells were fixed using 4% formaldehyde (Merck) in PBS. Then the cells were stained using crystal violet (0.1% in water, Merck) for 30 min, after which the plates were washed thoroughly in water and left to dry. Bound crystal violet was extracted using a 10% acetic acid solution (in water). To quantify the bound crystal violet in each well, the solution from the well was diluted tenfold with water and the absorbance was measured at 590 nM using an Infinite 200 PRO reader M-PLEX (Tecan).

#### Animal studies

All mice experiments were approved by the Netherlands Cancer Institute Animal Experimental Committee, and performed under the approval AVD30100202011584 WP 24.1.11064, 11 065 and 11 066 and were performed as previously described (45).

For testing of the optimal dosing of cisplatin *in vivo*, 5 × 10^6^ DU145 cells expressing the sfGFP UUA+1 reporter were injected into mammary gland #4 of 6- to 8-week-old NOD-SCID IL2R-null (jax) (NSG) mice. Tumor size was measured by caliper measurement and tumor volumes were calculated by the formula *V* = 1/2(*LW*^2^), where *L* corresponds to the length and *W* to the width of the tumor. After tumor volumes reached 100 mm^3^, mice (*n* = 3 per group) were either treated with mock treatment (saline), 3 mg/kg cisplatin or 6 mg/kg cisplatin for 72 h. Treatments were administered via intravenous (IV) injection. At the end of the experiment, tumor material was harvested and lysed in 6x the volume of RIPA buffer corresponding to the weight of the tumor. RIPA buffer consisted of 10 mM Tris-HCl pH 8.0, 140 mM NaCl, 0.1% SDS, 1% triton X-100, 1% sodium deoxycholate and protease inhibitors. Tumors were ground up using an ultra-TURAXX machine and the samples were sonicated with a sonicator probe. Protein contents were determined using a BCA analysis (Pierce), according to manufacturer’s instructions. In total, 50 μg of protein was loaded from each sample for immunoblot analysis.

For *in vivo* assessment of the efficacy of a combination of cisplatin and T cells, xenografts were established in 64 mice. Approximately 5 × 10^6^ DU145 cells expressing the sfGFP UUA+1-SIINFEKL reporter were injected into mammary gland #4 of 6- to 8-week-old NOD-SCID IL2R-null (jax) (NSG) mice (*n* = 32). Approximately 5 × 10^6^ DU145 cells expressing only H2-Kb were injected into mammary gland #4 of 6- to 8-week-old NOD-SCID IL2R-null (jax) (NSG) mice (*n* = 32). Tumor size was measured by caliper measurement and mice were randomized into four different treatment groups (*n* = 8 per group) when the average tumor size reached >50 mm^3^. Treatment groups were (i) mock treatment (saline) + 72 h later mock treatment (saline), (ii) mock treatment (saline) + 72 h later 5 × 10^6^ OT-I T cells, (iii) Cisplatin (6 mg/kg) + 72 h later mock treatment (saline) and (iv) Cisplatin (6 mg/kg) + 72 h later 5 × 10^6^ OT-I T cells. All treatments were administered via IV injection. On the day of OT-I T cell injection, and the two following days, 1 × 10^5^ IE of IL-2 (Proleukin, Clinigen) was administered via intraperitoneal injection. Tumor size was measured by caliper measurement three times a week.

### Data availability

Data were deposited in GEO with accession code GSE276695. Proteomics and immunopeptidomics data were deposited in PRIDE (46) with accession code PXD044085.

## Results

### Leucine shortage induces codon-biased ribosome stalling and frameshifting

**Figure 1.**
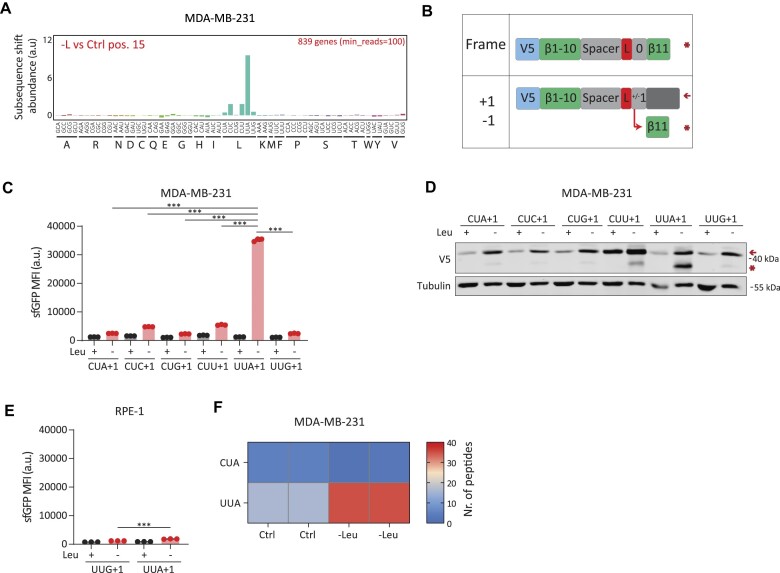
(**A**) Diricore analysis bar plots depicting differential codon usage (at position 15 of the RPFs) of leucine-depleted versus control MDA-MB-231 breast cancer cells. Data are the average from two biological replicates. (**B**) A scheme of the split superfolder GFP (sfGFP) reporters used for detection of frameshifting events at leucine codon. The coding sequence for the 11th β-sheet of sfGFP was placed either in-frame or out-of-frame (+1 or -1) downstream of leucine codons. * marks the full-length frameshifted sfGFP protein and ← marks the encoded out-of-frame protein, respectively. (**C**) Mean GFP fluorescence intensity (MFI) in MDA-MB-231 cells expressing +1 out-of-frame (+1) leucine reporters for all individual leucine codons. Data are mean ± s.d. of a representative experiment from two biological replicates. ***P < 0.001, ordinary one-way analysis of variance (ANOVA) using Sidak’s multiple testing correction. (**D**) Western blot (WB) showing V5 staining of MDA-MB-231 cells used in (C). Representative image shown from two biological replicates. (**E**) Bar plots representing MFI of GFP and in non-transformed epithelial RPE-1 cells expressing UUG+1 or UUA+1 reporters. Data are mean ± s.d. of a representative experiment from two biological replicates. ***P < 0.001, as per two-tailed t-test. (**F**) MDA-MB-231 cells were depleted from leucine and their proteomes were analyzed for trans-frame UUA- or CUA-derived peptides. The heat map depicts the total number of trans-frame peptides identified.

Leucine levels influence key cancer processes, such as mTORC1 signaling and epithelial-to-mesenchymal transition (47–49). In addition, LARS1 was shown to possess codon-specific tumor suppressive functions, which highlights the importance of this amino acid for cancer. To assess the consequences of leucine shortage on mRNA translation in tumor cells, we deprived MDA-MB-231, PC3 and MD55A3 cancer cell lines of leucine for 48 h and performed ribosome profiling (Supplementary Figure S1A) (50). First, we assessed the quality of our ribosome profiling using RiboWaltz and RUST analyses (Supplementary Figures S1B and D and S2) (32). The retrieved RPFs were then analyzed using diricore (differential ribosome codon reading) (31) to detect codon-specific ribosomal occupancies, which were validated using RUST A-site codon analysis (Figure 1A and Supplementary Figure S3). We expected the leucine shortage to stimulate enrichment of RPFs with the ribosomal A-site (position 15 from the 5′ end of each RPF) on all of the six leucine codons. Strikingly, our analysis revealed a prominent enrichment of RPFs with the ribosomal A-site mainly occupying UUA codons (Figure 1A and Supplementary Figure S3). This indicates the presence of a limiting factor that triggers a dominant ribosome stalling at one codon following leucine depletion.

Recent studies indicated that stalling during mRNA translation can lead to ribosomal frameshifting events (20,21,51). Therefore, we hypothesized that the UUA-enriched stalling of ribosomes following leucine depletion could lead to ribosomal frameshifting primarily on this codon. To examine this possibility, we engineered reporter constructs based on a self-complementing split superfolder GFP (sfGFP) gene (52). In these reporters, the tested leucine codons were introduced downstream of the last β-sheet 1–10 residue of sfGFP, with the last β-sheet (11th) of sfGFP placed out-of-frame in either the -1 or +1 direction (either removing a nucleotide or including an extra nucleotide, respectively) (Figure 1B). Only in the case of specific ribosomal frameshifting events fluorescent sfGFP signal will emerge (Figure 1B). We first expressed the engineered reporter constructs for the UUA codon in MD55A3 cells, and monitored sfGFP expression following leucine depletion by flow cytometry analysis. Frameshifting could be readily detected using either the -1 or +1 reporter in conditions of leucine starvation. However, a stronger sfGFP signal was detected for the UUA+1 construct as compared with UUA-1, indicating a preferential frameshifting in the +1-direction and the skipping of one nucleotide in conditions of amino acid shortage (Supplementary Figure S4A). We, therefore, introduced the +1-directed reporters for all six leucine codons in three individual cell lines, MDA-MB-231, PC3 and A549, and subjected these cells to leucine deprivation. Flow cytometry analysis showed sfGFP arising in all cell lines for each individual leucine codon following leucine depletion, suggesting that ribosomal frameshifts occur to a certain extent on all leucine codons in conditions of leucine shortage (Figure 1C; Supplementary Figure S4B and C). However, in agreement with the diricore analysis, the frameshifting assay showed a significantly higher signal at the UUA codon than all other leucine codons (Figure 1C; Supplementary Figure S4B and C). Immunoblot analysis using anti-V5-tag antibodies confirmed the detection of the chimeric frameshifted protein product with a smaller size predominantly from the UUA codon (Figure 1B and D). We measured mRNA levels of UUA+1 and UUG+1 reporters by qPCR, and confirmed no significant difference in mRNA expression between the two (Supplementary Figure S4D).

In line with previous findings showing that ribosomal frameshifting depends on oncogenic signaling (21), out-of-frame products were readily detected in various cancerous cell lines following leucine deprivation, but to a very limited extent in the non-cancerous cell lines RPE1 and BJ (Figure 1E and Supplementary Figure S4E–G). qPCR mRNA analysis of UUA+1 and UUG+1 indicated that while there is a significant difference between reporter expression between these cell lines (Supplementary Figure S4H), the difference is of a different order of magnitude compared to the observed signal for frameshifting (Supplementary Figure S4I).

Next, we expanded our observations made with the reporter constructs to endogenous proteins. We depleted MDA-MB-231 cells from leucine, performed mass spectrometry, and bioinformatically searched for frameshifted peptides derived from UUA codons. We controlled for specificity using an *in silico-*predicted proteome library of CUA-derived frameshifted peptides. We chose CUA as a control as both CUA and UUA are rare codons used at a rate of 0.72% and 0.77%, respectively (https://www.kazusa.or.jp/codon/cgi-bin/showcodon.cgi?species=9606). In addition, both codons have a 50% chance of generating a stop codon upon a +1 frameshift (UAA, UAG). Figure 1F shows that when cells are deprived of leucine, an enrichment of endogenously frameshifted peptides derived from UUA codons is detected, but not from control CUA codons (Supplementary Figure S4J and Supplementary Tables S4 and S5). Thus, our results indicate that leucine deprivation induces robust ribosome stalling and ribosomal frameshifting events primarily at one of the six leucine codons.

### Low tRNA^Leu^(UAA) level is the prime cause of preferential ribosomal stalling and aberrant protein production

Next, we wondered which underlying mechanism causes the restriction of mRNA translation primarily on UUA codons following leucine deprivation. UUA is one of the rare codons for leucine in the human protein-coding genome (0.77%). However, as we did not observe pronounced ribosomal stalling or frameshifting on the other rare leucine codon (CUA, 0.72%), codon abundance seems to be of minor importance in this context. Thus, we considered that a change in the supply and/or demand of tRNAs could be involved. To identify changes in demand, we analyzed the transcriptome of MDA-MB-231 cells in control and leucine-depletion conditions. This analysis indicated no significant changes in transcript levels induced by leucine shortage linked to their UUA codon content (Supplementary Figure S5A). Similarly, protein output was not differentially affected by leucine deprivation in relation to the UUA codon content of their respective transcript (Supplementary Figure S5B).

**Figure 2.**
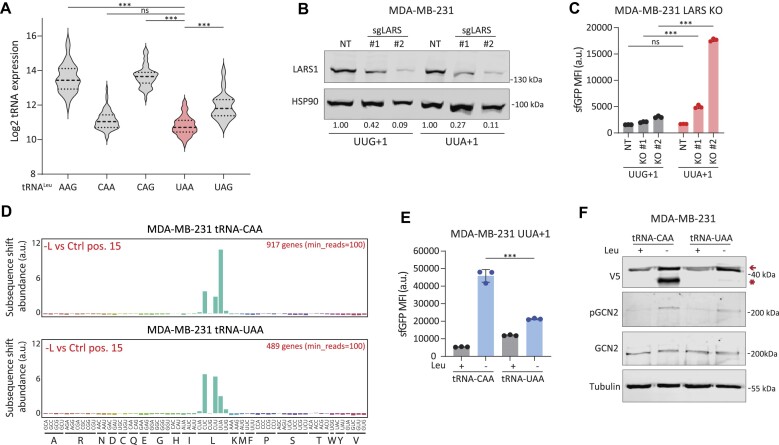
(**A**) Violin plots showing expression levels of individual leucine-tRNAs in the NCI-60 cell line panel. ***P < 0.001, ordinary one-way ANOVA using Sidak’s multiple testing correction. (**B**) Western blot showing LARS levels in MDA-MB-231 control cells (non-targeting/NT) or LARS KO (sgLARS). (**C**) Bar graph showing GFP MFI after LARS KO in MDA-MB-231 cells either expressing the UUG+1 or the UUA+1 reporter. Data are mean ± s.d. of a representative experiment from two biological replicates. ***P < 0.001, ordinary one-way ANOVA using Sidak’s multiple testing correction. (**D**) Diricore analysis bar plots depicting differential codon usage (at position 15 of the RPFs) in leucine-depleted versus control MDA-MB-231 cells with exogenous expression of tRNA^Leu^CAA (upper plot) or tRNA^Leu^UAA (lower plot). Data represent the average of from two biological replicates. (**E**and**F**) Fluorescence-activated cell sorting (FACS) (E) and western blot analysis (F) of MDA-MB-231 UUA+1-reporter cells expressing tRNA^Leu^CAA or tRNA^Leu^UAA. Representative experiments are shown from two biological replicates, data are mean ± s.d. ***P < 0.001, as per two-tailed t-test.

Since we did not find evidence for changes in the demand for tRNA^Leu^(UAA) for decoding the UUA codon, we examined the availability of tRNAs as the limiting factor for ribosomal translation in the event of leucine shortage. Using tRNA expression levels from the publicly available small non-coding RNA dataset generated from the NCI-60 cell line panel (33), we found that tRNA^Leu^(UAA) is generally the lowest expressed tRNA among all leucine tRNAs (Figure 2A). In addition, tRNA^Leu^(UAA) has the lowest expression level of all individual leucine tRNAs in each of the cell lines used in this study (Supplementary Figure S5C).

Hence, in conditions of leucine scarcity, a shortage in aminoacylated tRNA^Leu^(UAA) might become the critical limiting factor for the progression of mRNA translation. To test this idea independently of leucine depletion, we knocked out (KO) LARS1. As *LARS1* is an essential gene (53), two unique sgRNAs targeting *LARS1* were introduced into MDA-MB-231 and PC3 cells, and we measured its effect in a polyclonal population of cells (Figure 2B and Supplementary Figure S5D). Loss of LARS1 results in an abrogation of the aminoacylation of leucine tRNAs without affecting leucine levels. In these circumstances, uncharged leucine tRNAs will be produced due to ongoing mRNA translation. We expected that the leucine tRNAs with the most limiting levels for mRNA translation would be the ones to induce ribosomal stalling, followed by frameshifting on their cognate codons. Indeed, *LARS1* knockout induced ribosomal frameshifting predominantly on the UUA codon (Figure 2C and Supplementary Figure S5E). Of note, due to the essentiality of *LARS1*, its effect was lost over time (data not shown).

To further confirm that uncharged tRNA^Leu^(UAA) is the limiting factor upon depletion, we examined the levels of aminoacylated tRNAs. This indicated a significant increase in the levels of uncharged leucine-tRNAs following leucine depletion (Supplementary Figure S5F). Together, these results point towards a more general uncharging of leucine tRNAs after leucine deprivation, while tRNA^Leu^(UAA) is more specifically restricting the outcome on mRNA translation due to its low abundance.

We corroborated these results by exogenously expressing tRNA^Leu^(UAA), thereby increasing its expression levels and its availability for mRNA translation (Supplementary Figure S6A and B). Analyses of the ribosome profiles of these cells indicated specific mitigation of the ribosome stalling at UUA codons by ectopic expression of tRNA^Leu^(UAA), but not tRNA^Leu^(CAA) (Figure 2D; Supplementary Figure S6C and Supplementary Figure S7A,B). Interestingly, stalling on CUC and CUU codons is increased upon rescue of stalling on UUA codons, suggesting that tRNA^Leu^(AAG) that decodes both these codons becomes limiting in this context.

Using the reporter assay for UUA-induced frameshifting, we confirmed that exogenous tRNA^Leu^(UAA) expression, but not tRNA^Leu^(CAA), significantly suppressed frameshifting capacity on the UUA codon in conditions of leucine shortage (Figure 2E and F). Thus, these results suggest that the low abundance of tRNA^Leu^(UAA) is the prime cause of ribosomal stalling and frameshifting preferentially at one leucine codon out of the six potentially affected ones.

### DNA damage induces ribosomal frameshifting on UUA codons

**Figure 3.**
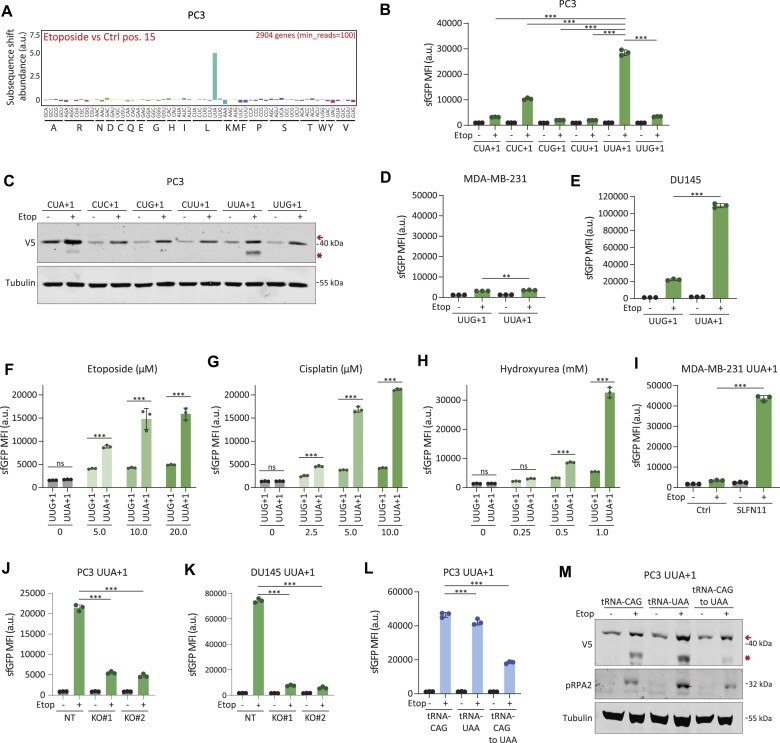
(**A**) Diricore analysis bar plots depicting differential codon usage (at position 15 of the RPFs) in etoposide-treated versus control PC3 prostate cancer cells. Data are the average from two biological replicates. (**B**and**C**) FACS analysis (B) and western blot (C) of PC3 cells expressing+1 out-of-frame (+1) leucine reporters for all leucine codons subjected to etoposide treatment. Representative experiments are shown from two biological replicates, data are mean ± s.d. ***P < 0.001, ordinary one-way ANOVA using Sidak’s multiple testing correction. (**D–E**) GFP mean fluorescence intensity (MFI) in SLFN11-negative MDA-MB-231 cells (D) or SLFN11-positve DU145 cells (E) expressing UUA+1 or UUG+1 constructs and subjected to etoposide treatment. Data are mean ± s.d. of a representative experiment from two biological replicates. ***P < 0.001, **P = 0.008, ordinary one-way ANOVA using Sidak’s multiple testing correction. (**F–H**) MFI of GFP in A549 UUA+1 and UUG+1 reporter cells subjected to etoposide (F), cisplatin (G) or hydroxyurea (H). Data are mean ± s.d. of a representative experiment from two biological replicates. ***P < 0.001, two-way ANOVA using Sidak’s multiple testing correction. (**I**) MFI of GFP in MDA-MB-231 UUA+1 cells with exogenous SLFN11 expression and treated with etoposide. Data are mean ± s.d. of a representative experiment from two biological replicates. ***P < 0.001, as per two-tailed t-test. (**J**and**K**) The effect of SLFN11 knockout (KO) on GFP frameshifting in PC3 UUA+1 cells (J) and DU145 UUA+1 cells (K) following etoposide treatment. Data are mean ± s.d. of a representative experiment from two biological replicates. ***P < 0.001, two-way ANOVA using Sidak’s multiple testing correction. (**L**and**M**) FACS analysis (L) and western blot (M) of PC3 UUA+1 reporter cells, expressing tRNA^Leu^(CAG), tRNA^Leu^(UAA) and tRNA^Leu^(CAG to UAA) and subjected to etoposide treatment. FACS data are mean ± s.d. of a representative experiment from two biological replicates. ***P < 0.001, two-way ANOVA using Sidak’s multiple testing correction.

Interestingly, activation of Schlafen Family Members 11 and 12 (SLFN11 and SLFN12), enzymes with ribonuclease activity towards type II tRNAs, lead to a prominent ribosomal stalling on UUA codons, in a similar fashion to what we observed for leucine starvation (14,16,23,54,55). Thus, we hypothesized that following SLFN11 activation upon DNA damage, SLFN11-induced tRNA^Leu^(UAA) shortage would induce ribosomal stalling and frameshifting on UUA codons in cancer cells, comparable to conditions of leucine shortage. To confirm the link between SLFN11 activity and ribosomal stalling, we first examined ribosomal occupancy in SLFN11-positive PC3 cells treated with the chemotherapeutic agent etoposide (Supplementary Figure S8A and B). As expected, we observed a prominent ribosomal accumulation preferentially on UUA codons, similar to the stalling observed in leucine-depleted conditions (Figure 3A and Supplementary Figure S8C–E)). Importantly, this ribosomal stalling was accompanied by frameshifting, as a robust fluorescent signal was significantly higher in PC3 cells expressing the UUA+1 reporter as compared to the signal observed from the reporters for all other leucine codons (Figure 3B and C). We corroborated these conclusions using UUA+1 and UUG+1 frameshift reporter constructs in SLFN11-negative MDA-MB-231 and SLFN11-positive PC3, DU145, A549 and SNB19 cells. Etoposide treatment induced a robust frameshift-derived fluorescent signal only in SLFN11-positive cells expressing the UUA+1 reporter, whereas SLFN11-negative cells showed only minimal levels of sfGFP induction (Figure 3D and E; Supplementary Figure S9A–C). Importantly, all tested replication stress-inducing agents (etoposide, cisplatin and hydroxyurea) provoked high levels UUA-codon-derived ribosomal frameshifting, whereas DNA damage induced by ionizing radiation did not (Figure 3F–H and Supplementary Figure S9D). To further confirm the causal role of SLFN11 in replication stress-induced ribosomal frameshifting, we either ectopically expressed SLFN11 in SLFN11-negative MDA-MB-231 cells, or knocked it out in the SLFN11-positive PC3, DU145 and A549 cells (Supplementary Figure S9E–H). In both cases, the presence of SLFN11 was clearly associated with the capacity to induce robust ribosomal frameshifting at UUA codons following etoposide treatment (Figure 3I–K and Supplementary Figure S9I).

To further confirm that SLFN11-induced shortage in tRNA^Leu^(UAA) is causal to ribosomal frameshifting after induction of replication stress, we ectopically expressed different leucine-decoding tRNAs. Expression plasmids for tRNA^Leu^(CAG), tRNA^Leu^(UAA) and tRNA^Leu^(CAG > UAA), which encodes a mutated tRNA^Leu^(CAG) where the anticodon is modified to UAA (CAG > UAA), were introduced in SLFN11 positive cell lines (Supplementary Figure S9J–L). Since SLFN11 most effectively cleaves tRNA^Leu^(UAA) (54,55), we expected tRNA^Leu^(CAG > UAA) to be resistant to SLFN11-mediated cleavage and thus be able to suppress etoposide-induced ribosomal frameshifting at UUA codons. Whereas exogenous expression of tRNA^Leu^(UAA) was able to mitigate frameshifting in conditions of leucine deprivation (Figure 2E), it only had limited effects on frameshifting after etoposide treatment (Figure 3L and M; Supplementary Figure S9M and N), since it is still a target for SLFN11-mediated cleavage. As expected, SLFN11-resistant tRNA^Leu^(CAG > UAA) expression substantially suppressed ribosomal frameshifting after etoposide treatment compared to control tRNA^Leu^(CAG) (Figure 3L and M; Supplementary Figure S9M and N). Altogether, these results indicated that both leucine shortage and chemotherapy-induced DNA replication stress limit the availability of tRNA^Leu^(UAA) for mRNA translation to induce ribosomal stalling and frameshifting events predominantly on UUA codons in cancer cells.

### HLA presentation and immune recognition of UUA-derived aberrant peptides

**Figure 4.**
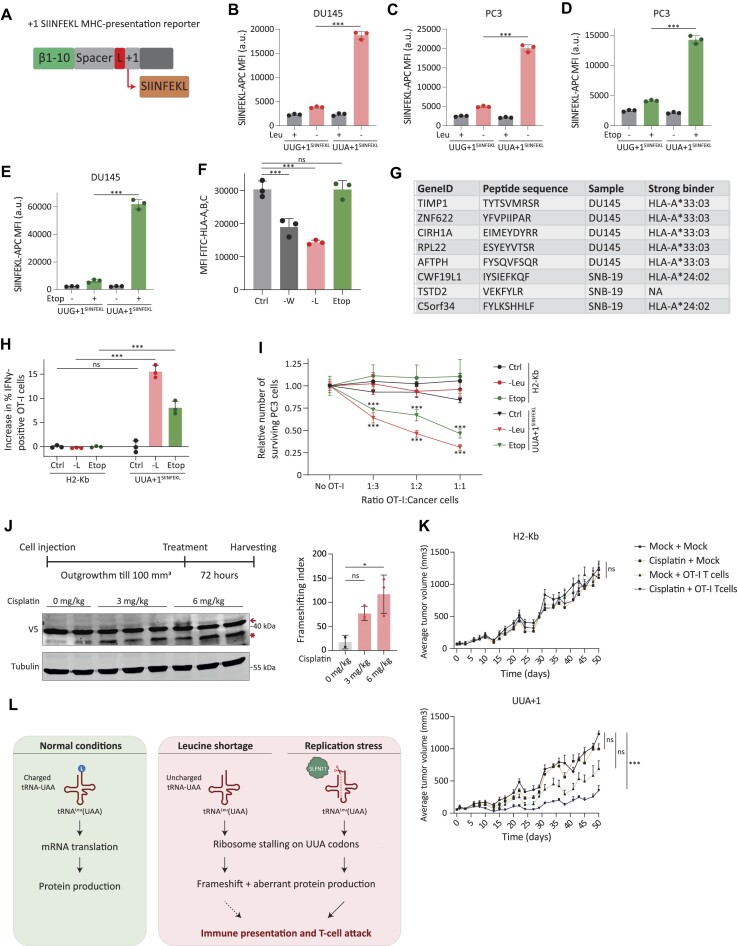
(**A**) Schematic representation of the combined frameshift-immune presentation reporter. The SIINFEKL model peptide presented on MHC-I molecules is +1 out-of-frame following a UUG (UUG+1) or UUA (UUA+1) codon. (**B–E**) MFI of H-2K^b^-bound SIINFEKL in PC3 and DU145 cells expressing H-2K^b^ in combination with UUG+1 and UUA+1 SIINFEKL reporters. Cells were analyzed after either leucine depletion (B and C) or etoposide treatment (D and E). Data are the mean ± s.d. of two independent experiments, ***P < 0.001, as per two-tailed t-test. (**F**) Pan-HLA levels after indicated treatments as determined by FACS staining. Representative experiment shown from two biological replicates, data are mean ± s.d. ***P < 0.001, ordinary one-way ANOVA using Sidak’s multiple comparison test. (**G**) Table showing trans-frame chimeric peptides specifically induced on UUA codons after etoposide treatment in DU145 and SNB-19 cells. (**H**) OT-I T cell activation as measured by IFN-y positivity after co-culture with pretreated PC3 H2-Kb control and UUA+1-SIINFEKL-expressing cells. Representative experiment shown from three biological replicates, data are mean ± s.d. *** *P* < 0.001, ordinary one-way ANOVA using multiple comparison test. (**I**) Anti-tumor cell activity of OT-I T cells in co-cultures with pretreated H2-Kb control and UUA+1-SIINFEKL-expressing cells. Representative experiment shown from three biological replicates, data are mean ± s.d. ***P < 0.001, ordinary two-way ANOVA using Sidak’s multiple comparison test. (**J**) Immunoblot analysis of xenografted DU145 cells expressing the UUA+1 reporter treated with the indicated treatments. Quantification of the frameshifting index (frameshifted product/in-frame product) is represented as a bar graph, data are the mean ± s.d. * *P* = 0.02, ordinary one-way ANOVA using multiple comparison test. (**K**) Anti-tumor cell effect of OT-I T cells *in vivo* toward H2-Kb control and UUA+1-SIINFEKL-expressing cells, that were pre-treated with mock-treatment or 6 mg/kg of cisplatin (*n* = 8 mice per group). Data are mean + SEM, *** *P* < 0.001, two-way ANOVA using Sidak’s multiple comparison test. (**L**) schematic representation of the effect of leucine shortage and replication stress on charged tRNA-Leu-UUA abundance and subsequent production of UUA-derived aberrant peptides.

Ribosomal frameshifting-derived peptides are considered to be defective ribosomal products (DRiPs) that are efficiently processed and presented at the cell surface to elicit T-cell activation (56). We, therefore, examined whether UUA-derived frameshifted peptides produced as a consequence of leucine depletion or replication stress can be presented at the cell surface to the immune system. We used the SIINFEKL model peptide from chicken ovalbumin and placed it downstream of our frameshift reporter constructs (UUA+1^SIINFEKL^ and UUG+1^SIINFEKL^, Figure 4A) (57). These constructs were introduced into PC3 and DU145 cells expressing H2-K^b^, the major histocompatibility complex class I molecule (MHC-I) presenting SIINFEKL peptides. In line with our previous results, flow cytometry analysis indicated a robust induction of frameshift-derived SIINFEKL at the cell surface of cells expressing the UUA+1^SIINFEKL^ reporter as compared with UUG+1^SIINFEKL^, following either leucine depletion or etoposide treatment (Figure 4B–E).

Encouraged by the UUA+1^SIINFEKL^ detection following leucine deprivation and chemotherapy treatment, we set out to identify endogenous UUA-induced frameshifted peptides in the immunopeptidome of cancer cells. We first examined the effect of both treatments on the overall levels of human leukocyte antigen class I (HLA-I) molecules at the cell surface. Flow cytometry analysis with a pan anti-HLA-I antibody (recognizing HLA-A, B and C) indicated that etoposide treatment largely unaffected HLA levels, whereas leucine deprivation led to a general reduction thereof (Figure 4F). Therefore, we focused on etoposide treatment and performed immunopeptidomics analysis on SLFN11 positive cells to identify treatment-induced immune-presented aberrant peptides (neoepitopes). To bioinformatically search for these neoepitopes, we generated an immunopeptidomics library containing chimeric peptides derived from UUA and control CUA codons. To ensure full confidence in the obtained hits, we only considered trans-frame peptides consisting of an in-frame peptide sequence followed by an out-of-frame part, as this ensures the specific identification of codon-specific frameshift-derived peptides only. In addition, all truncated peptides potentially induced by reading of a UAA or UAG stop codon after a +1 frameshift were excluded. This analysis showed that etoposide treatment specifically induced eight UUA-derived chimeric neoepitopes, whereas only one such CUA-derived neoepitope was detected after treatment (Figure 4G). Supporting the specificity of the detected neoepitopes, almost all of these (7 out of 8) were predicted to strongly bind the HLA alleles expressed in these cells (Supplementary Figures S10 and S11). Overall, these results demonstrate the induction of UUA-codon-derived neoepitope production from an endogenous route after treatment with chemotherapeutic agents.

Chemotherapy-induced neoepitope presentation can potentially be utilized for tumor cell killing via T-cell targeting. To examine this potential, we first used co-culture experiments using a model system of SLFN11-positive UUA+1^SIINFEKL^ cells with OT-I T cells, which recognize the SIINFEKL peptide presented by H2-K^b^ (57). T-cell activation was measured by flow cytometry using intracellular IFNγ accumulation in T cells as a marker for their activation. This experiment indicated that both etoposide and leucine deprivation treatments of UUA+1^SIINFEKL^ cells elicited OT-I T-cell activation, whereas H2-kb control cells that do not express the inducible neoepitope could not induce T-cell activation in any of the conditions. (Figure 4H). In line with these results, we observed a potent OT-I T-cell-killing response toward UUA+1^SIINFEKL^ cells pretreated with either etoposide or leucine-deprivation, as compared to untreated controls (Figure 4I). Hence, these results form a proof-of-concept that UUA-derived neoepitopes, induced either by leucine depletion or etoposide treatment, can potentially mediate T-cell attacks toward cancer cells.

While in the context of the UUA+1^SIINFEKL^ reporter vector, both chemotherapeutic agents and leucine deprivation led to T-cell recognition and killing (Figure 4H and I), we observed a suppression of cell surface levels of endogenous HLA molecules and, therefore, reduced neoepitope presentation by leucine deprivation (Figure 4F). This hampers further utilization of leucine deprivation for T-cell therapy *in vivo*. We, therefore, combined chemotherapy and T-cell therapy targeting UUA+1-derived SIINFEKL neoepitopes in a xenograft model of a human cancer cell line. First, to gain insight into the dynamics of the chemotherapy-induced aberrant protein expression, we tested whether and under which conditions systemic treatment with chemotherapy induces an optimal production of aberrant proteins in cancer cells *in vivo*. We transplanted the SLFN11-positive DU145 cell line containing the sfGFP UUA+1 reporter vector in the mammary fat pads of NOD-Scid IL2Rg-null mice. Once tumors reached 100 mm^3^ in volume, mice received either cisplatin (3 or 6 mg/kg) or mock treatment, and tumors were harvested for anti-V5 immunoblot analysis 72 h later. Indeed, we readily detected robust aberrant protein production with either of the tested doses of cisplatin, with 6 mg/kg giving rise to a robust and significant induction of ribosomal frameshifting (Figure 4J). It is important to note that some aberrant protein products were also observed in the mock-treated xenografts, potentially due to a tumor-induced leucine shortage in the tumor microenvironment.

Having validated and optimized the induction of aberrant proteins following systemic administration of cisplatin, we next examined whether the induction of frameshift-derived neoepitopes can elicit an anti-tumor T-cell response *in vivo*. Xenografts from DU145 cells expressing either H2-K^b^/UUA+1^SIINFEKL^ or control H2-K^b^ vectors were treated with cisplatin (6 mg/kg) or mock treatment, and 72 h later received a tail vein injection with either a mock treatment or OT-I T cells (Figure 4K). As expected, no significant effect on tumor growth was observed with any of the treatments in H2-K^b^ control xenografts (Figure 4K), which lack the inducible neoepitope. Interestingly, while the DU145 H2-K^b^/UUA+1^SIINFEKL^ tumor growth was refractory to cisplatin treatment and only mildly affected by administration of OT-I T (in line with the spontaneous slight induction of the SIINFEKL aberrant protein (Figure 4J), cisplatin followed by administration of T cells led to a significant, potent, and durable response on tumor growth (Figure 4K). Altogether, these results show that a combination of chemotherapeutic agents followed by administration of aberrant-peptide specific T cells has the potential to significantly impact tumor growth *in vivo*.

## Discussion

In this study, we demonstrate that both DNA damage and leucine depletion induce aberrant protein production, primarily at UUA codons. While both types of treatment can potentially induce a broad response on mRNA translation, a general limitation in availability of tRNA^Leu^(UAA) for protein production restricts the outcome. This ultimately leads to prominent ribosomal stalling at UUA codons and aberrant protein production from these codons in cancer cells. While leucine deprivation restricts HLA expression and limits peptide presentation, treatment with chemotherapeutic agents does not, thus permitting the presentation of induced neoepitopes. In addition, we provide a proof-of-concept that these inducible neoepitopes can serve as potential targets for a T-cell attack and can impact tumor growth following systemic treatment with chemotherapy (Figure 4L).

Interestingly, UUA-codon-specific regulation of mRNA translation was demonstrated to control the expression of DNA damage response genes in reaction to replication stress and to attenuate viral protein production as a viral defense mechanism (14,16,55,58). Our results suggest that these cellular responses could be exploited to induce aberrant protein production and neoepitope presentation in cancer cells that can potentially serve as T-cell targets (22).

It is important to note that subgroups of breast cancer and high-grade serous ovarian carcinoma patients with high SLFN11 expression showed a prominent association with immune activation after treatment with chemotherapy (59,60). Functionally, platinum treatments were specifically shown to induce tumor-immune transactivation in a SLFN11-dependent manner, suggesting that SLFN11 plays a key role in immune activation (60). This chemotherapy-induced immune activation could stem from various sources, with the possibility that UUA-codon-derived neopitopes could play a role here, which warrants further investigations.

Accumulating pieces of evidence have indicated that chemotherapeutic agents potentiate anti-tumor immune responses via multiple routes. These DNA-damaging agents enhance the release of neoantigens via the induction of cell lysis and by an mRNA translation-coupled increase in HLA class I presentation (61–63). In addition, the innate immune response is activated through direct DNA double-strand break-dependent induction of the cGAS-STING (stimulator of interferon genes) axis (61). By identifying the induction of codon-biased aberrant protein and neoepitope production following DNA damage, our study uncovers a novel connection between treatment with chemotherapeutic agents and the immune system.

Even though we observed abundant ribosomal stalling at UUA codons after treatment with chemotherapeutic agents, only a limited number of immune-presented aberrant chimeric peptides were identified in these conditions. This might be the result of a multitude of factors. First, around half of the +1 frameshifts occurring at UUA codons will result in termination of translation due to the reading of a stop codon (UAA or UAG). This limits the number of potentially produced chimeric proteins compared to other codons. Second, it has been shown that only few of all unique neoantigens are efficiently processed and bind with high affinity to a given HLA molecule (64,65). Third, we applied strict criteria for identifying ribosomal frameshift-derived neoepitopes, considering only chimeric peptides. Nonetheless, we identified several chemotherapy-induced ribosomal frameshift-derived neoepitopes for two major HLA types, all of which are predicted to be strong binders.

Frameshift-derived neoantigens are thought to possess intrinsically high immunogenicity, as they are fundamentally different in their composition from self-epitopes (66,67). This type of neoantigens is even thought to be the main contributor to the success of immune checkpoint blockade (ICB) therapy in certain types of cancer. In particular, in renal cell carcinomas, where high levels of immune infiltrates and a high response rate to ICB therapy were observed (68). This type of cancer harbors a high level of frameshift mutations but relatively few other genetic aberrations, indicating that frameshift-derived neoantigens are the main targets of the observed immune response (68,69). As frameshift-derived neoepitopes seem to have high immunogenicity, and effective T-cell reactivity is already observed towards only few immune-presented neoantigens per tumor (70), further exploration of the potential of the inducible neoepitopes identified in this study is warranted.

Altogether, we identified a novel link between the effect of chemotherapeutic agents and aberrant mRNA translation in cancer. This unexpected route of inducible ribosomal frameshifting at UUA codons may be valuable for neoepitope generation in tumor cells with a low mutational burden.

## Supplementary Material

gkae1110_Supplemental_File

## Data Availability

Data were deposited in GEO with accession code GSE276695. Proteomics and immunopeptidomics data were deposited in PRIDE (46) with accession code PXD044085.
