# Supplementary material for: Chemotherapeutic agents and leucine deprivation induce codon-biased aberrant protein production in cancer
Source: Nucleic Acids Res. 2024 Nov 26;52(22):13964–79. doi: 10.1093/nar/gkae1110 (PMC11662694; doi:10.1093/nar/gkae1110)
Supplement: gkae1110_Supplemental_File [file gkae1110_supplemental_file.pdf]

Supplementary Figure S1

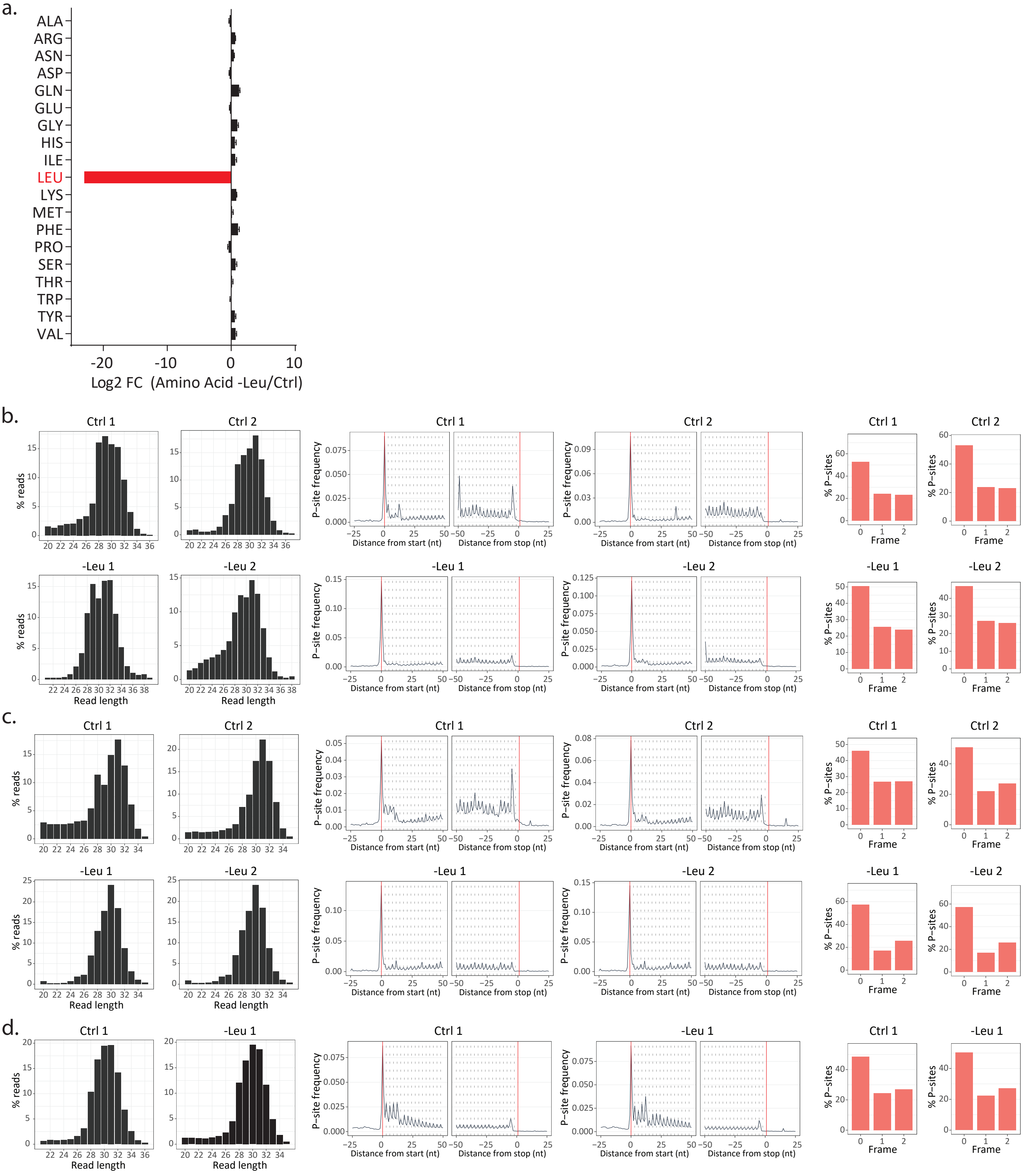

Figure S1. a, Cellular amino acid levels as determined by mass spectrometry in control conditions or 48hr after leucine depletion. Each bar represents the average of three independent experiments ±s.d. **b-d.** RiboWaltz quality control plots for Riboseq experiments for control and -Leu conditions for MDA-MB-231 **(b)**, PC3 **(c)** and MD55A3 **(d)** cells. Plots represent (from left to right) read length distribution, P-site distribution and periodicity and frame distribution over the coding sequence.

Supplementary Figure S2

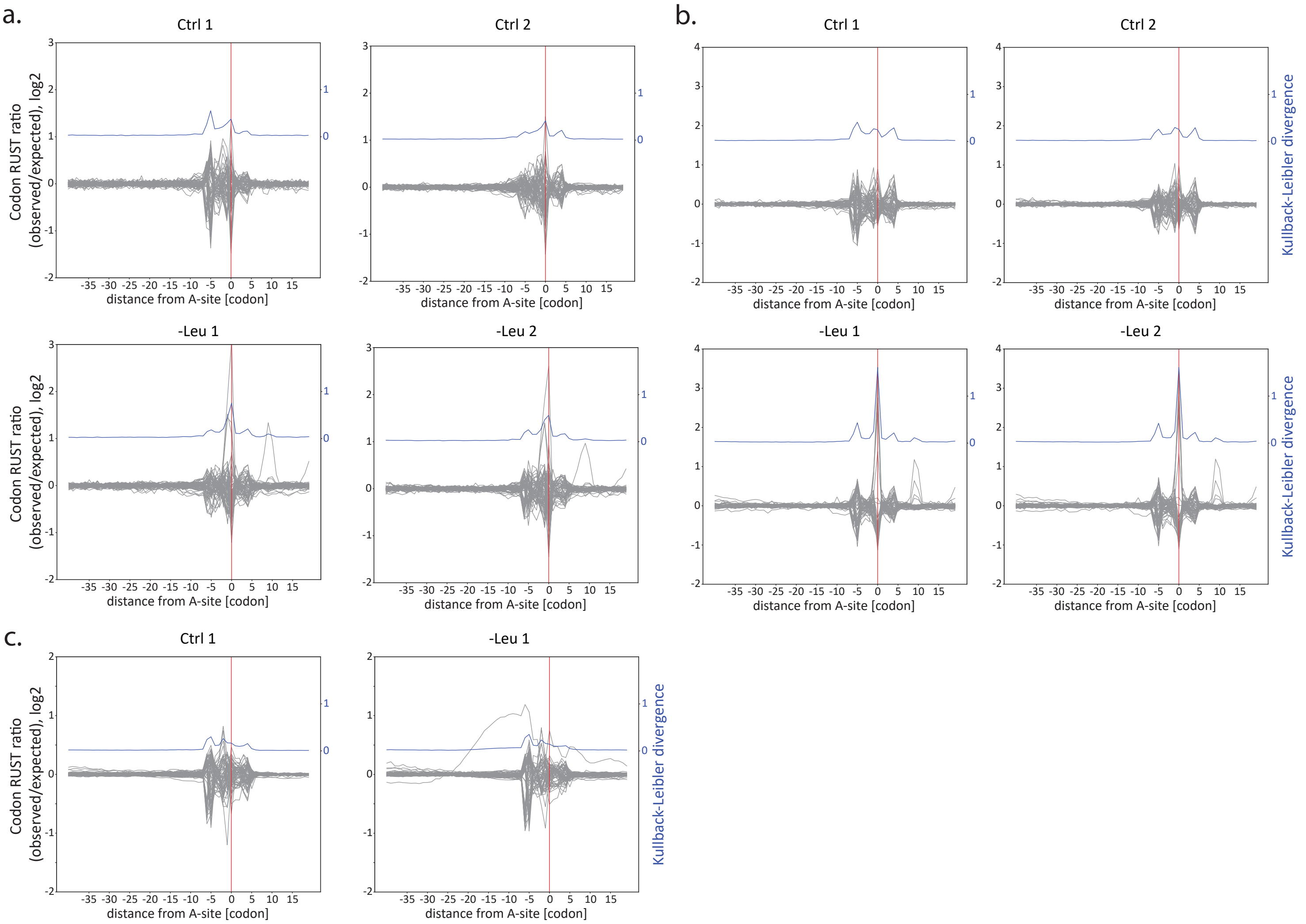

Figure S2. a-c, RUST codon analyses for Riboseq experiments for control and -Leu conditions for MDA-MB-231 **(a)**, PC3 **(b)** and MD55A3 **(c)** cells.

Supplementary Figure S3

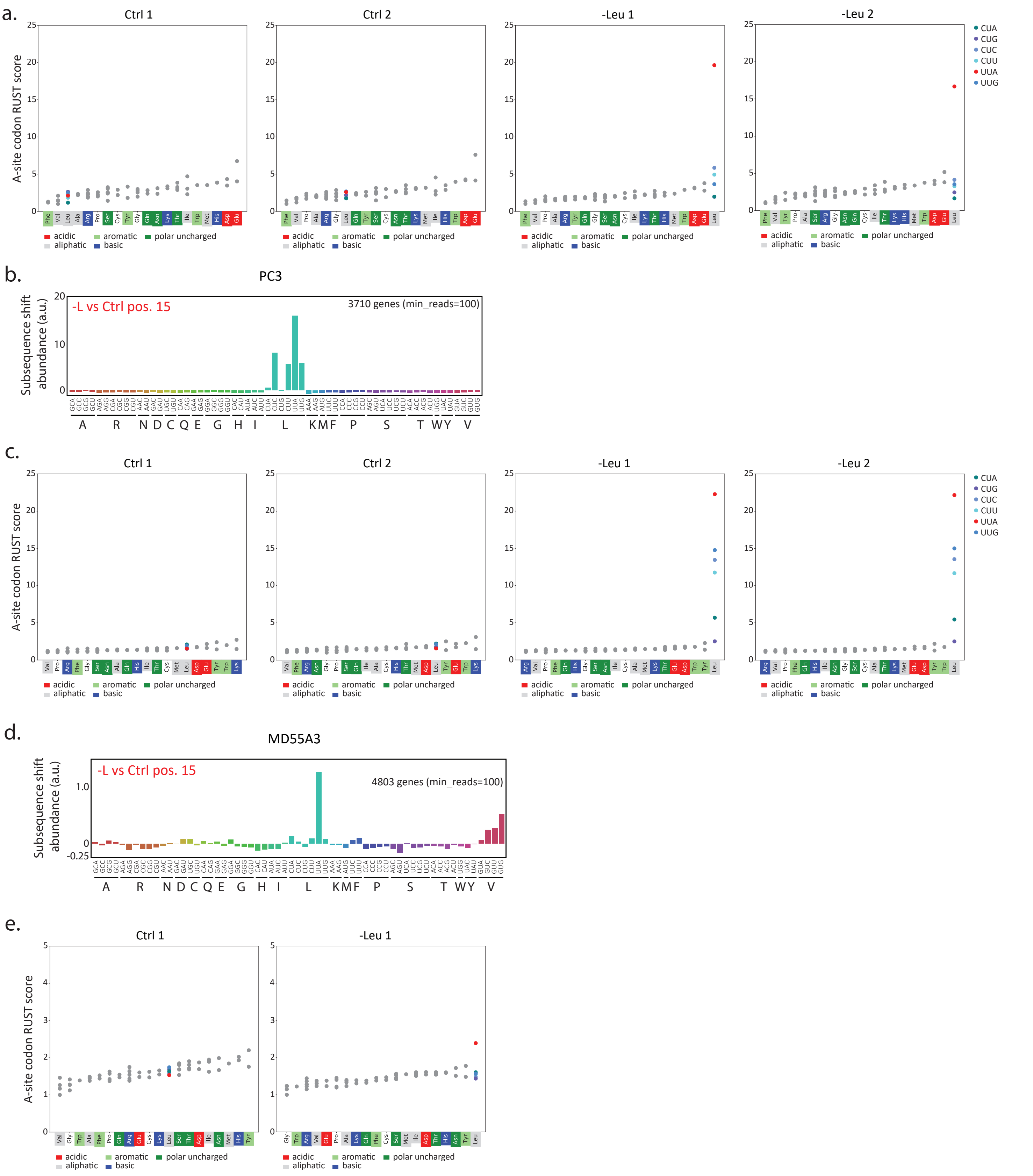

Figure S3. a, Graphs representing RUST A-site codon score for Riboseq experiments for control and -Leu conditions for MDA-MB-231 cells. **b,** Diricore analyses depicting differential codon usage (at position 15 of the RPFs) in leucine-depleted versus control PC3 prostate cancer cells. Data represent the average from two biological replicates. **c,** Graphs representing RUST A-site codon score for Riboseq experiments for control and -Leu conditions for PC3 cells. **d,** Diricore analyses depicting differential codon usage (at position 15 of the RPFs) in leucine-depleted versus control MD55A3 melanoma cancer cells. **e,** Graphs representing RUST A-site codon score for Riboseq experiments for control and -Leu conditions for MD55A3 cells.

Supplementary Figure S4

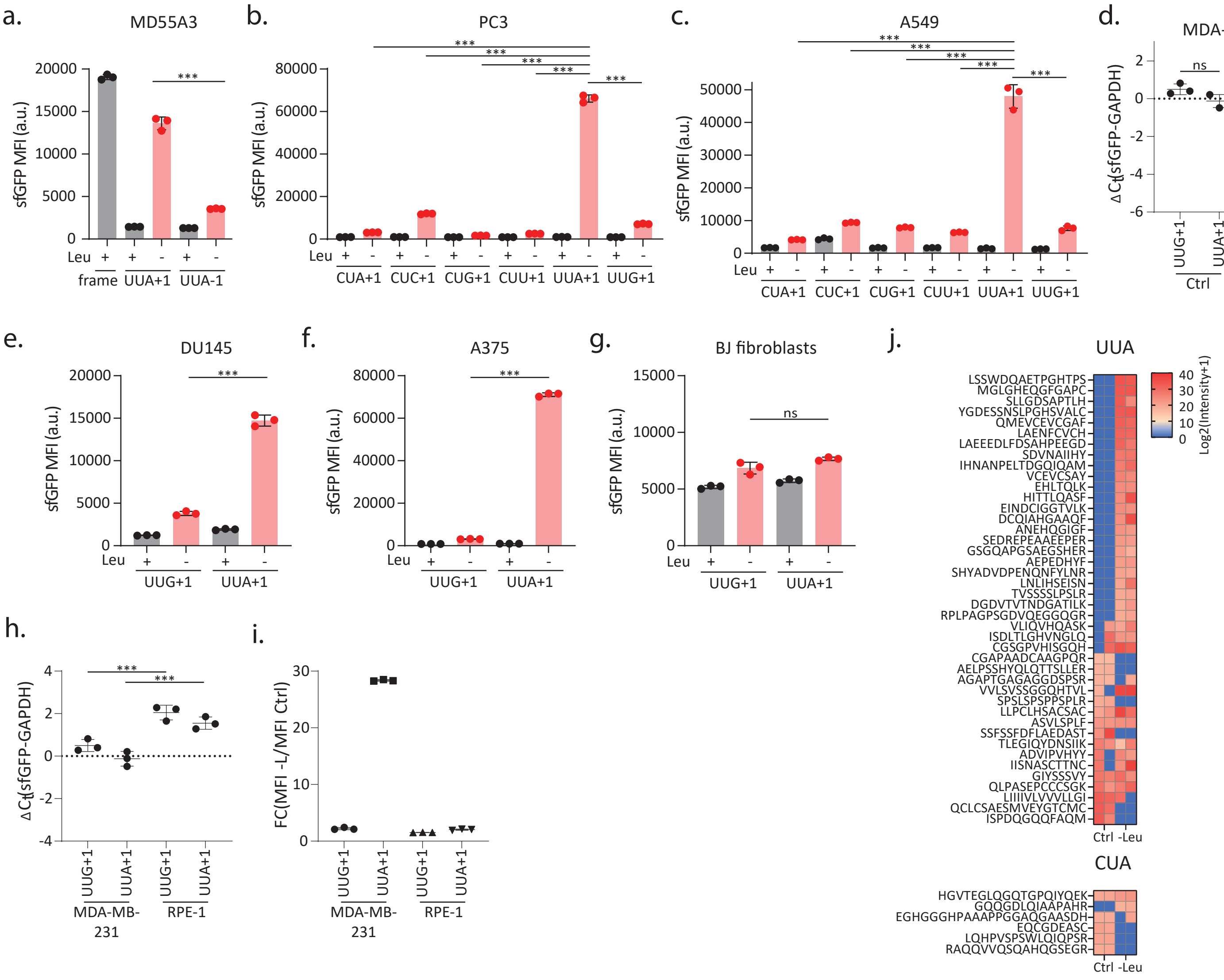

Figure S4. a, Bar plots representing MFI of GFP in MD55A3 cells expressing in-frame, UUA +1 and -1 reporters, Data are mean ±s.d. of representative experiment from two biological replicates. *** P < 0.001, as per two-tailed t test. **b-c**, Bar plots representing MFI of GFP in PC3 prostate cancer cells **(b)** and A549 lung cancer cells **(c)** expressing +1 out-of-frame (+1) reporters for all leucine codons. Data are mean ±s.d. of a representative experiment from two biological replicates. *** P < 0.001, ordinary one-way ANOVA using Sidak's multiple testing correction. **d**, Comparison of mRNA expression levels of the UUG+1 and UUA+1 reporters in MDA-MB-231 cells. Statistical significance was tested using an ordinary one-way ANOVA with Sidak's multiple testing correction. **e-g,** Bar plots representing MFI of GFP originating from UUA+1 and UUG+1 reporters in the indicated cell lines in ctrl and leucine depletion conditions. Data are mean ±s.d. of a representative experiment from two biological replicates. *** P < 0.001, as per two-tailed t test. **h,** Comparison of mRNA expression levels of the UUG+1 and UUA+1 reporters in MDA-MB-231 and RPE-1 cells. *** P < 0.001, ordinary one-way ANOVA using Sidak's multiple testing correction. **i,** Graph showing the fold change of fluorescent intensity from UUG+1 and UUA+1 reporters in MDA-MB-231 and RPE-1 cells from Figure 1c and 1e. **j,** Heat map showing UUA- or CUA-derived trans-frame peptides identified in the full proteome of leucine depleted MDA-MB-231 cells in two biological replicates.

Supplementary Figure S5

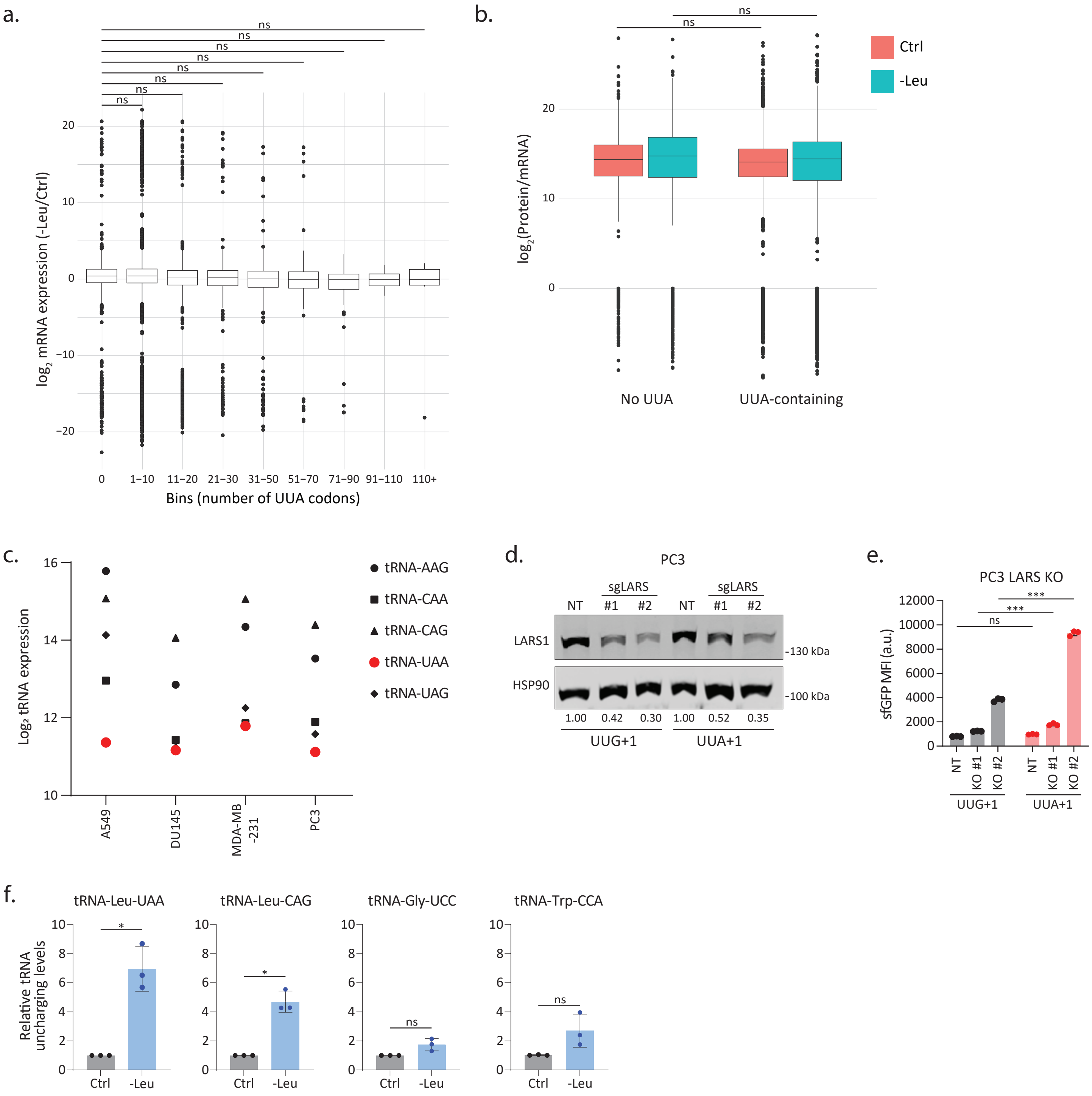

Figure S5. a, Log2 mRNA expression (-Leu/Ctrl) in MDA-MB-231 cells. Transcripts were binned according to the number of UUA codons present in the coding sequence. Statistical significance was tested using an ordinary one-way ANOVA with Sidak's multiple testing correction. **b,** Log2 (Protein/mRNA) ratio in MDA-MB-231 cells in control and leucine-depleted conditions. Groups were assigned by virtue of absence or presence of UUA codons in the coding sequence. Statistical significance was tested using a paired Welch's t-test. **c,** Expression levels of all individual leucine tRNAs in each of the cell lines used in this study. tRNA-Leu-UAA is indicated in red. Data were obtained from the publicly available non-coding RNA expression database of NCI-60 cell lines panel. **d,** Western blot analysis showing CRISPR-Cas9 knockouts of LARS1 or non-targeting (NT) control in PC3 cells expressing UUA+1 and UUG+1 constructs. Representative image shown from two biological replicates. **e,** The effect of LARS1 knockouts or NT control on GFP mean fluorescence intensity in PC3 cells expressing the UUG+1 or UUA+1 reporter. Data are the mean ±s.d. of a representative experiment from two biological replicates. *** $P < 0.001$, ordinary one-way ANOVA using Sidak's multiple testing correction. **f,** Bar plots representing the relative tRNA uncharging levels for control and leucine deprivation in MDA-MB-231 cells. * $P < 0.033$, as per paired two-tailed t test.

Supplementary Figure S6

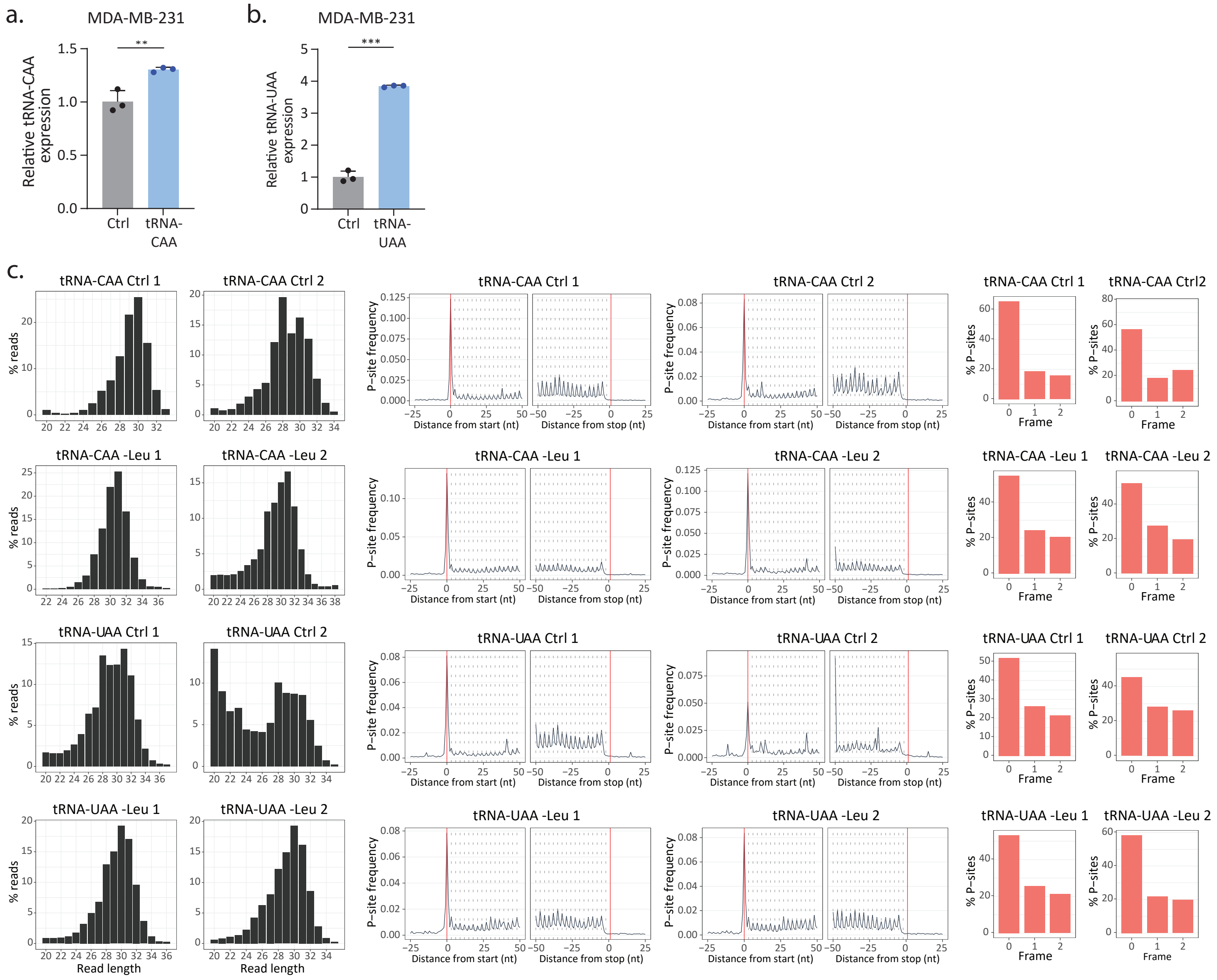

Figure S6. a-b, Relative tRNA expression levels in MDA-MB-231 measured by qRT-PCR. Representative experiment shown from two biological replicates, data are mean ±s.d. *** P < 0.001, ** P = 0.008 as per two-tailed t test. **c,** RiboWaltz quality control plots for Riboseq experiments for control and -Leu conditions for MDA-MB-231 cells exogenously expressing tRNA-Leu-CAA (top) tRNA-Leu-UAA (botom).

Supplementary Figure S7

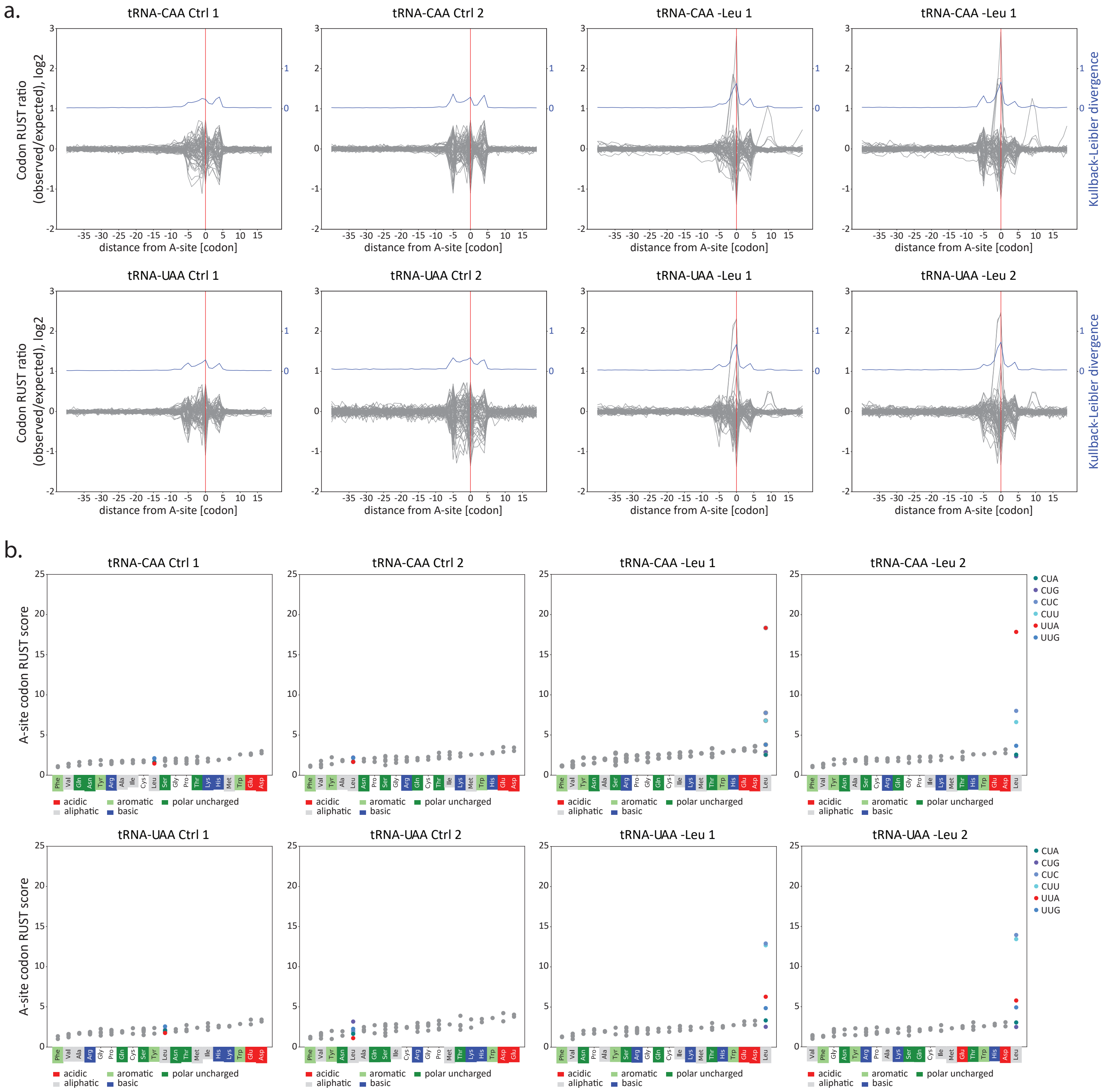

Figure S7. a, RUST codon analyses for Riboseq experiments for control and -Leu conditions for MDA-MB-231 cells exogenously expressing tRNA-Leu-CAA (top) tRNA-Leu-UAA (botom). **b,** Graphs representing RUST A-site codon score for Riboseq experiments for control and -Leu conditions for MDA-MB-231 cells exogenously expressing tRNA-Leu-CAA (top) tRNA-Leu-UAA (botom).

Supplementary Figure S8

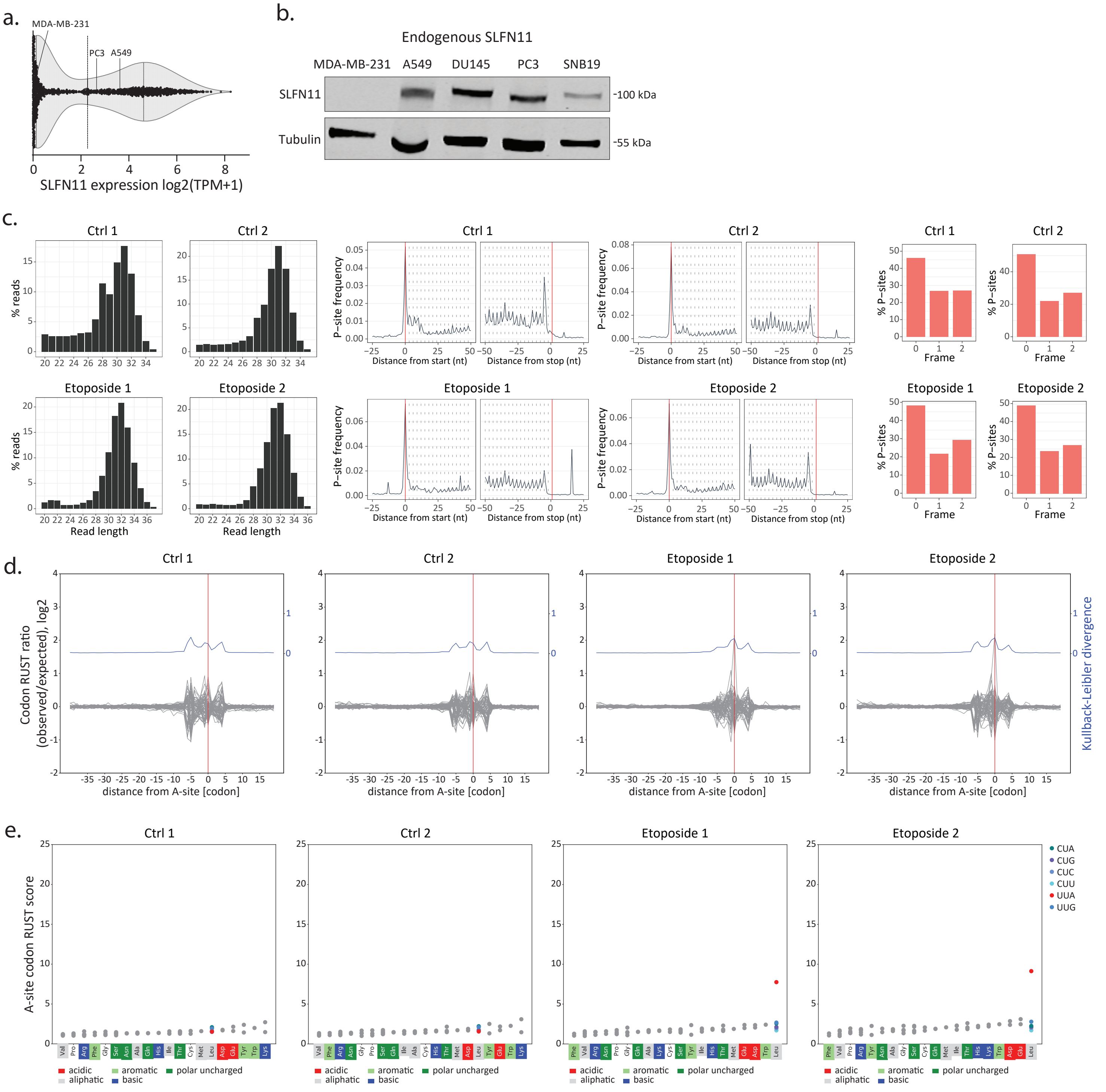

Figure S8. a, Expression of SLFN11 across cell lines from the cancer cell line encyclopedia. **b,** Western blot analysis of endogenous SLFN11 levels in MDA-MB-231, A549, DU145, PC3 and SNB19 cells. Representative image shown from two biological replicates. **c,** RiboWaltz quality control plots for Riboseq experiments of control and etoposide-treated conditions for PC3 cells. **d,** RUST codon analyses for Riboseq experiments for control and etoposide-treated conditions for PC3 cells. **e,** Graphs representing RUST A-site codon score for Riboseq experiments of control and etoposide-treated conditions for PC3 cells.

Supplementary Figure S9

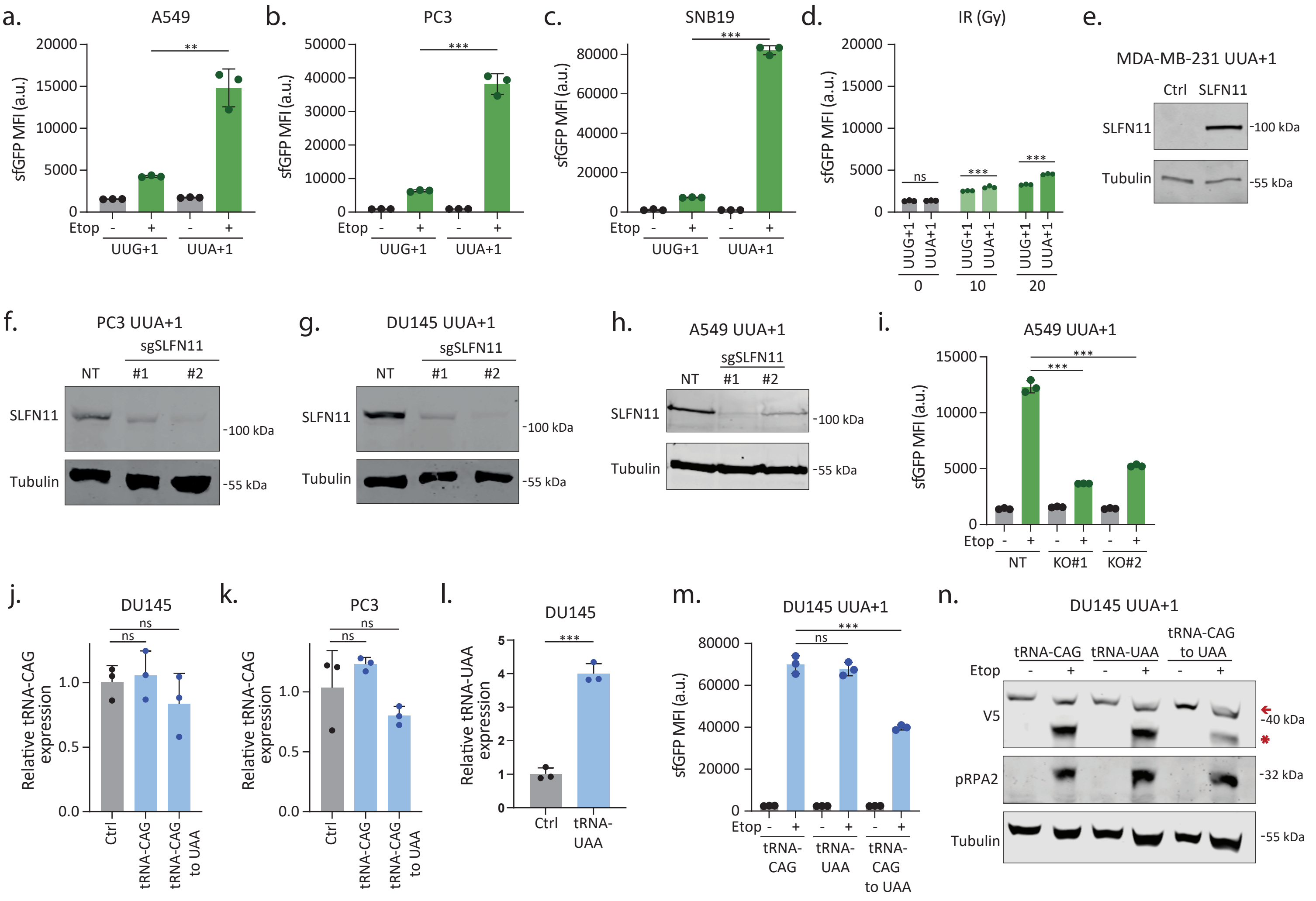

Figure S9. a-c, Mean fluorescence intensity (MFI) of GFP in A549 **(a)** , PC3 **(b)** and SNB-19 cells **(c)** expressing UUA+1 or UUG+1 reporter constructs subjected to etoposide treatment. Representative experiment shown from two biological replicates, data are mean ±s.d. *** P < 0.001, ** P = 0.001 as per two-tailed t test. **d,** Mean fluorescence intensity (MFI) of GFP in A549 with UUA+1 and UUG+1 reporters and subjected to ionizing radiation. Representative experiment shown from two biological replicates, data are mean ±s.d. *** P < 0.001, two-way ANOVA using Sidak's multiple testing correction. **e,** Western blot analysis for SLFN11 expression in parental MDA-MB-231 and cell lines with exogenous SLFN11 expression. Representative image shown from two biological replicates. **f-h,** Western blot analysis for SLFN11 knockout in SLFN11-positive cell lines- PC3 **(f)**, DU145 **(g)** and A549 **(h)**. Representative images shown from two biological replicates. **i,** The effect of SLFN11 knockout (KO) in A549 UUA+1 cells on GFP mean fluorescence intensity (MFI) following etoposide treatment. Representative experiment shown from two biological replicates, data are mean ±s.d. *** P < 0.001, ordinary one-way ANOVA using Sidak's multiple testing correction. **j-l,** Relative tRNA expression levels in DU145 and PC3 as measured by qRT-PCR. Representative experiment shown from two biological replicates, data are mean ±s.d. *** P < 0.001 as per two-tailed t test. **m-n,** FACS analysis **(m)** and western blot **(n)** of DU145 UUA+1 reporter cells, with exogenous expression of tRNA-Leu(CAG), tRNA-Leu(UAA), and tRNA-Leu(CAG to UAA), which were subjected to etoposide treatment. Representative experiment shown from two biological replicates, data are mean ±s.d. ***P < 0.001, ordinary one-way ANOVA using Sidak's multiple testing correction.

Supplementary Figure S10 Quality control plots generated using MHCVizPipe from immunopeptidomics analysis from DU145 cells.

MhcVizPipe (v0.7.9) - Analysis report

CARONLAB

Date: 2024-03-21

Submitted by: Anonymous

Analysis type: Class I

Description of experiment: None provided

Samples:

DU145_ctrl_P651_1_peptide.tsv:
Alleles: HLA-A0301, HLA-A3303, HLA-B5001, HLA-B5701, HLA-C0602
DU145_ctrl_P651_3_peptide.tsv:
Alleles: HLA-A0301, HLA-A3303, HLA-B5001, HLA-B5701, HLA-C0602
DU145_ctrl_P651_2_peptide.tsv:
Alleles: HLA-A0301, HLA-A3303, HLA-B5001, HLA-B5701, HLA-C0602
DU145_etop_P651_1_peptide.tsv:
Alleles: HLA-A0301, HLA-A3303, HLA-B5001, HLA-B5701, HLA-C0602
DU145_etop_P651_2_peptide.tsv:
Alleles: HLA-A0301, HLA-A3303, HLA-B5001, HLA-B5701, HLA-C0602
DU145_etop_P651_3_peptide.tsv:
Alleles: HLA-A0301, HLA-A3303, HLA-B5001, HLA-B5701, HLA-C0602

Sample Overview

- LF Score: fraction of peptides between 8 and 12 mers.
- BF Score: fraction of peptides between 8 and 12 mers which are predicted to be strong or weak binders.

Sample	Total peptides	Peptides between 8-12 mers	LF Score	BF Score
DU145_ctrl_P651_1_peptide.tsv	9871	9338	0.95	0.93
DU145_ctrl_P651_3_peptide.tsv	9681	9214	0.95	0.93
DU145_ctrl_P651_2_peptide.tsv	10387	9859	0.95	0.93
DU145_etop_P651_1_peptide.tsv	8198	7668	0.94	0.92
DU145_etop_P651_2_peptide.tsv	8916	8453	0.95	0.93
DU145_etop_P651_3_peptide.tsv	9100	8635	0.95	0.93

UpSet Plot (only displaying intersections containing >= 1% of at least one sample)

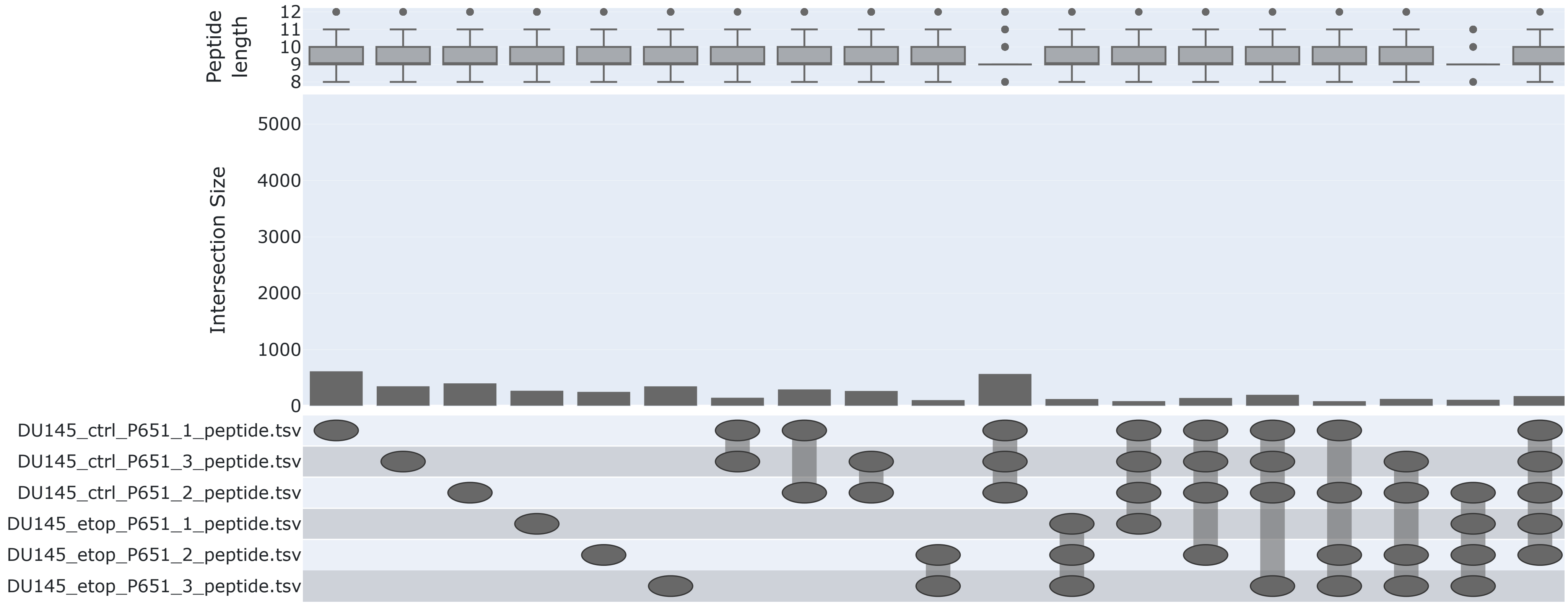

Peptide Length Distribution (maximum of 30 mers)

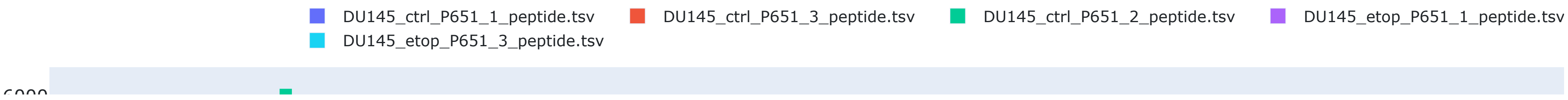

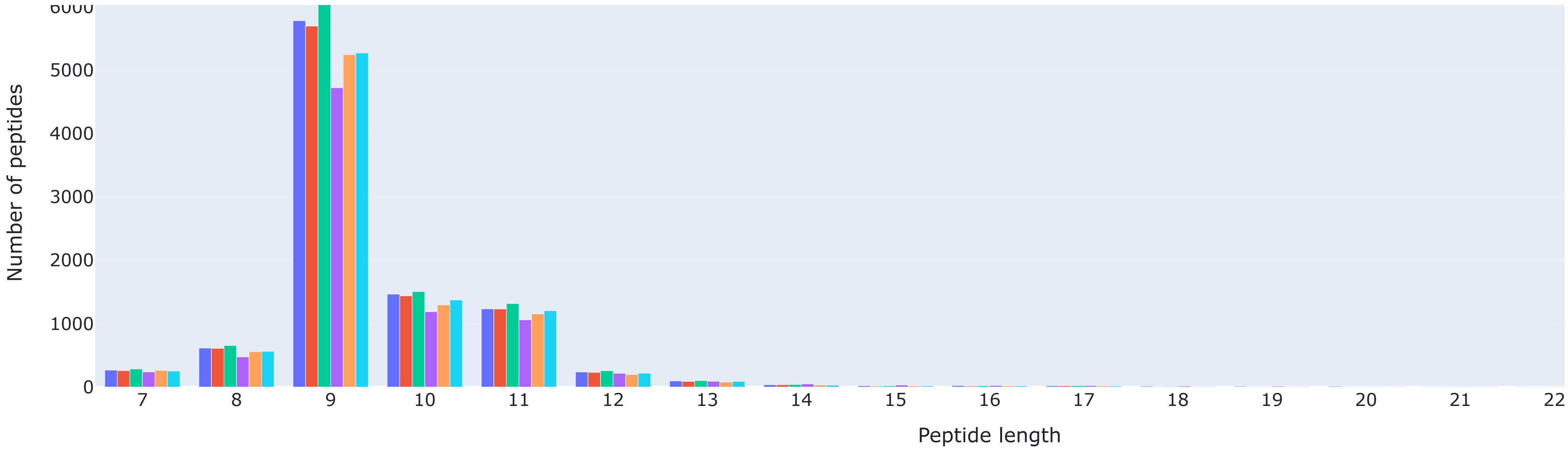

Annotation Results

NetMHCpan eluted ligand predictions made for all peptides between 8 & 12 mers, inclusive.

- Percent rank cutoffs for strong and weak binders: 0.5 and 2.0.
- Percentages are calculated across rows (i.e. percentage of total peptides for a respective sample).

Sample	Total peptides	Allele	Strong binders	Weak binders	Non-binders
DU145_ctrl_P651_1_peptide.tsv	9338	HLA-A0301	141 (1.5%)	556 (6.0%)	8641 (92.5%)
		HLA-A3303	3675 (39.4%)	806 (8.6%)	4857 (52.0%)
		HLA-B5001	1532 (16.4%)	170 (1.8%)	7636 (81.8%)
		HLA-B5701	1331 (14.3%)	215 (2.3%)	7792 (83.4%)
		HLA-C0602	1158 (12.4%)	422 (4.5%)	7758 (83.1%)
DU145_ctrl_P651_3_peptide.tsv	9214	HLA-A0301	144 (1.6%)	563 (6.1%)	8507 (92.3%)
		HLA-A3303	3658 (39.7%)	801 (8.7%)	4755 (51.6%)
		HLA-B5001	1506 (16.3%)	159 (1.7%)	7549 (81.9%)
		HLA-B5701	1302 (14.1%)	207 (2.2%)	7705 (83.6%)
		HLA-C0602	1158 (12.6%)	404 (4.4%)	7652 (83.0%)
DU145_ctrl_P651_2_peptide.tsv	9859	HLA-A0301	149 (1.5%)	592 (6.0%)	9118 (92.5%)
		HLA-A3303	3901 (39.6%)	877 (8.9%)	5081 (51.5%)
		HLA-B5001	1549 (15.7%)	164 (1.7%)	8146 (82.6%)
		HLA-B5701	1411 (14.3%)	223 (2.3%)	8225 (83.4%)
		HLA-C0602	1246 (12.6%)	456 (4.6%)	8157 (82.7%)

Binding Affinities

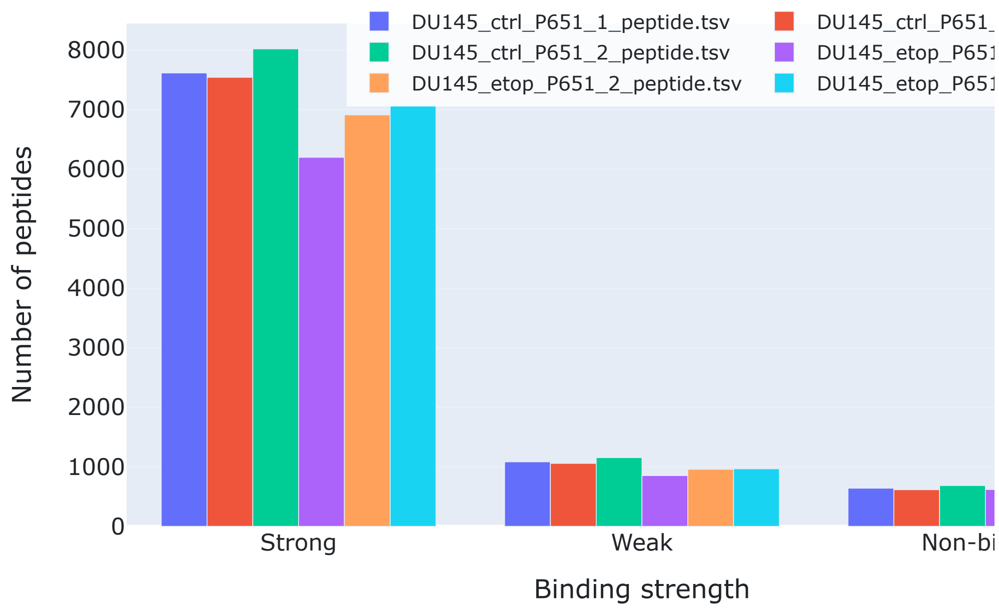

Sample	Total peptides	Allele	Strong binders	Weak binders	Non-binders
DU145_etop_P651_1_peptide.tsv	7668	HLA-A0301	123 (1.6%)	489 (6.4%)	7056 (92.0%)
		HLA-A3303	3459 (45.1%)	694 (9.1%)	3515 (45.8%)
		HLA-B5001	1282 (16.7%)	88 (1.1%)	6298 (82.1%)
		HLA-B5701	945 (12.3%)	120 (1.6%)	6603 (86.1%)
		HLA-C0602	561 (7.3%)	309 (4.0%)	6798 (88.7%)
DU145_etop_P651_2_peptide.tsv	8453	HLA-A0301	132 (1.6%)	543 (6.4%)	7778 (92.0%)
		HLA-A3303	3753 (44.4%)	767 (9.1%)	3933 (46.5%)
		HLA-B5001	1439 (17.0%)	108 (1.3%)	6906 (81.7%)
		HLA-B5701	1139 (13.5%)	134 (1.6%)	7180 (84.9%)
		HLA-C0602	640 (7.6%)	355 (4.2%)	7458 (88.2%)
DU145_etop_P651_3_peptide.tsv	8635	HLA-A0301	131 (1.5%)	561 (6.5%)	7943 (92.0%)
		HLA-A3303	3859 (44.7%)	769 (8.9%)	4007 (46.4%)
		HLA-B5001	1455 (16.9%)	111 (1.3%)	7069 (81.9%)
		HLA-B5701	1167 (13.5%)	146 (1.7%)	7322 (84.8%)
		HLA-C0602	651 (7.5%)	374 (4.3%)	7610 (88.1%)

Binding Heatmaps

NetMHCpan eluted ligand predictions made for all peptides between 8 & 12 mers, inclusive.
Approximate color legend (detailed mapping shown next to heatmaps):

Predicted strong binders (%rank <= 0.5) Predicted weak binders (0.5 < %rank <= 2.0) Predicted non-binders - - # of peptides in sample

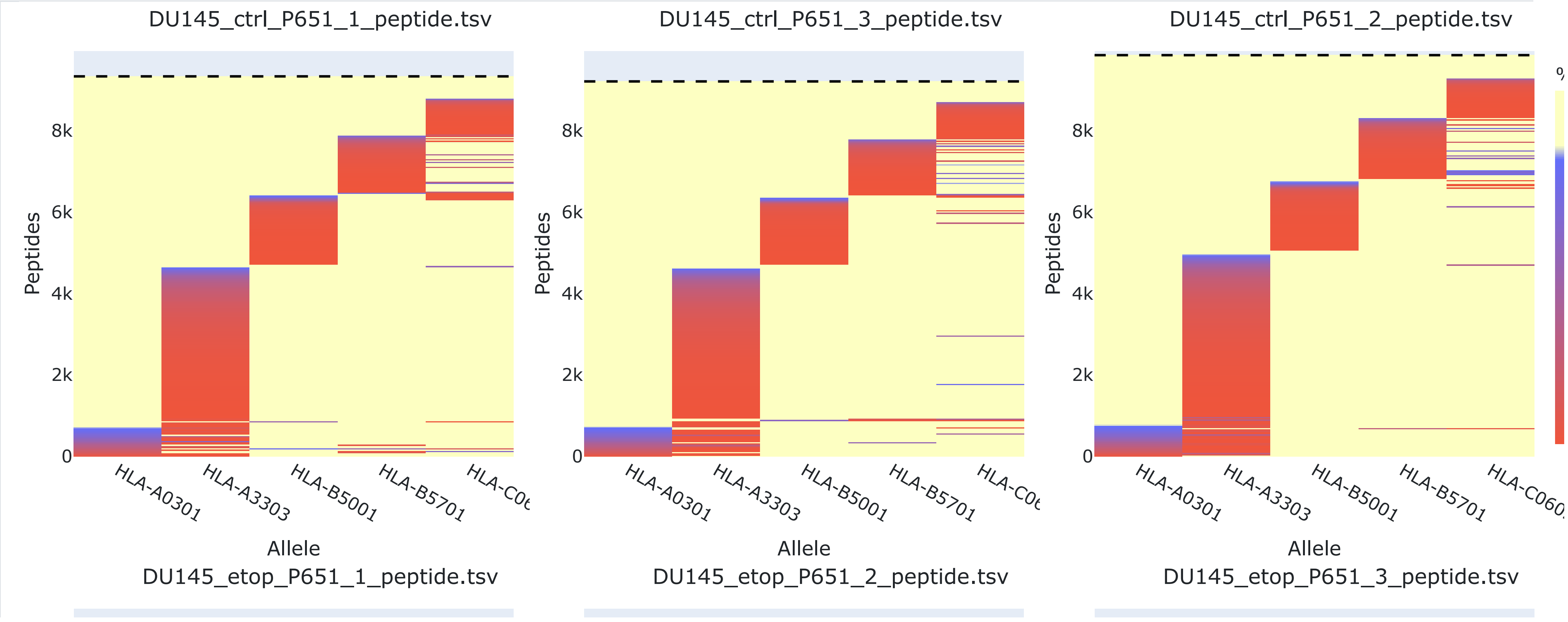

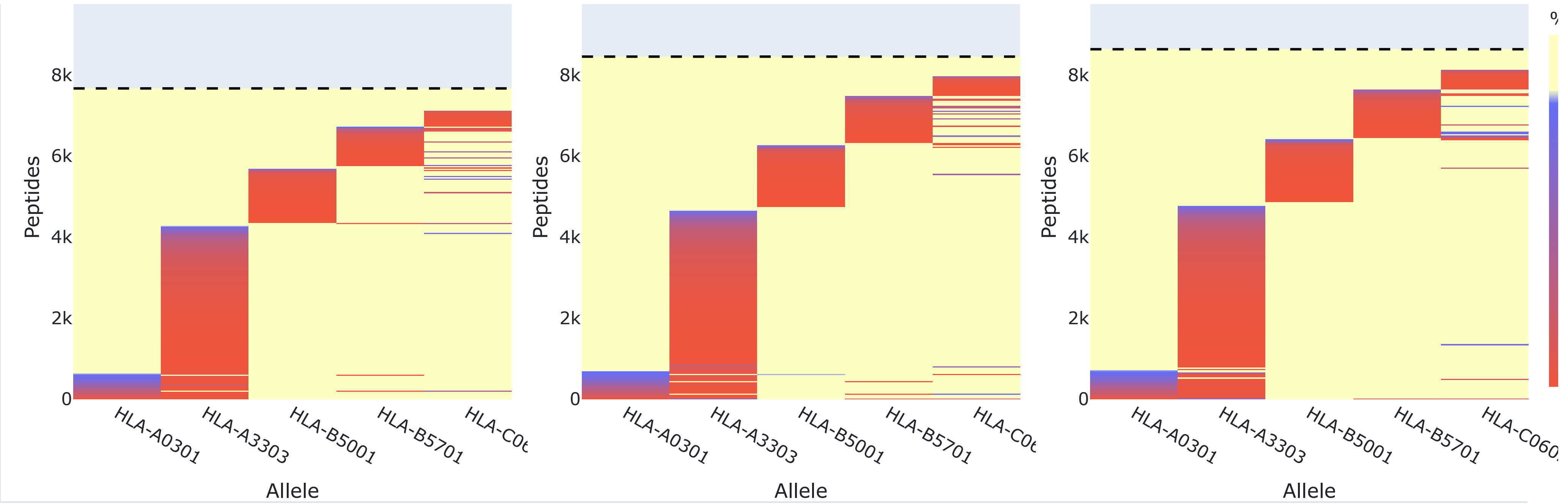

Sequence Motifs

Clustering performed with all peptides between 8 & 12 mers, inclusive.

- Percentages represent the percentage of peptides in a given group predicted to strongly bind the indicated allele.

Polar Neutral Basic Acidic Hydrophobic

Unsupervised GibbsCluster Allele-specific GibbsCluster

DU145_ctrl_P651_1_peptide.tsv (peptides clustered: 9338, outliers: 233)

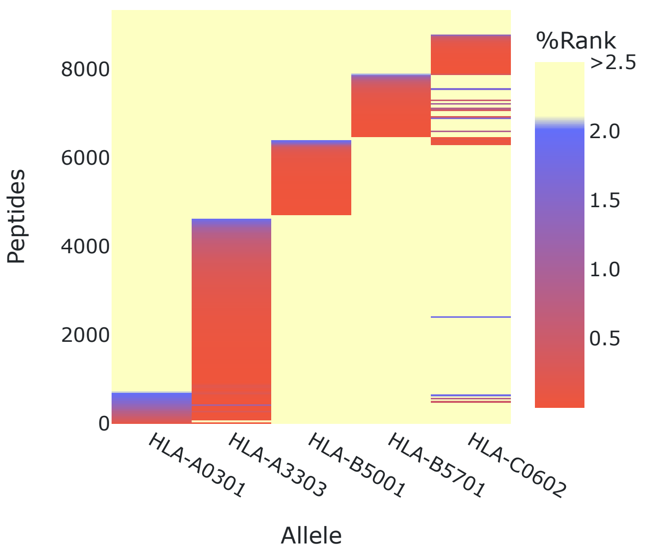

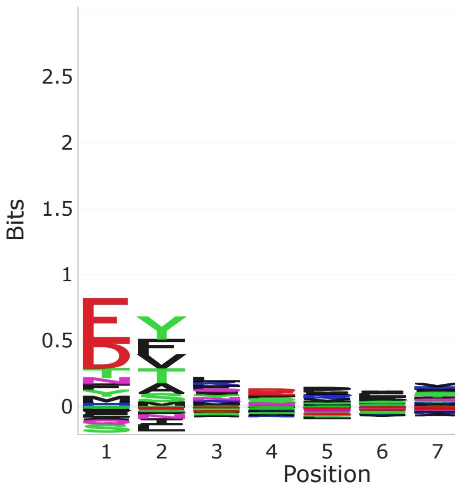

Peptides in group: 4773
HLA-A0301: 2%, **HLA-A3303: 77%,** HLA-B5001: 0%, HLA-B5701: 0%, HLA-C0602: 0%

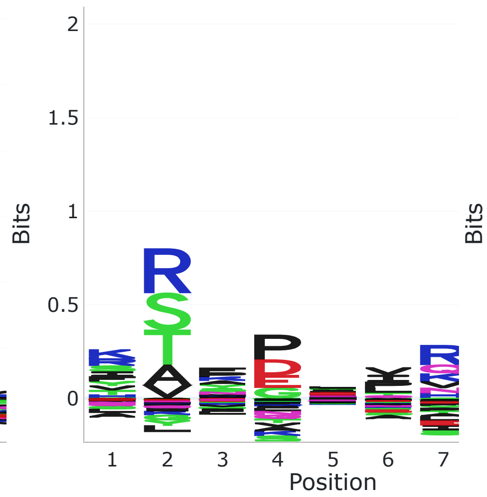

Peptides in group: 2657
HLA-A0301: 1%, HLA-A3303: 0%, HLA-B5001: 0%, **HLA-B5701: 49%,** HLA-C0602: 42%

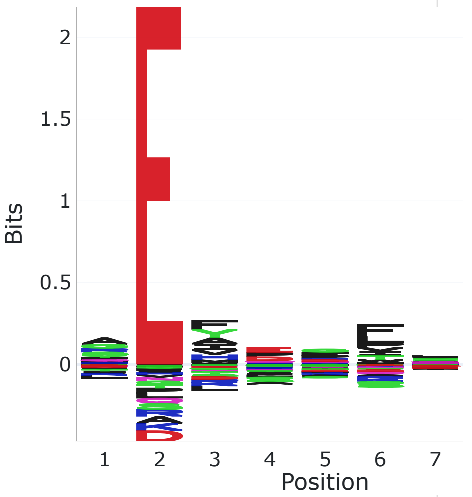

Peptides in group: 1675
HLA-A0301: 0%, HLA-A3303: 0%, **HLA-B5001: 91%,** HLA-B5701: 0%, HLA-C0602: 1%

DU145_ctrl_P651_3_peptide.tsv (peptides clustered: 9214, outliers: 224)

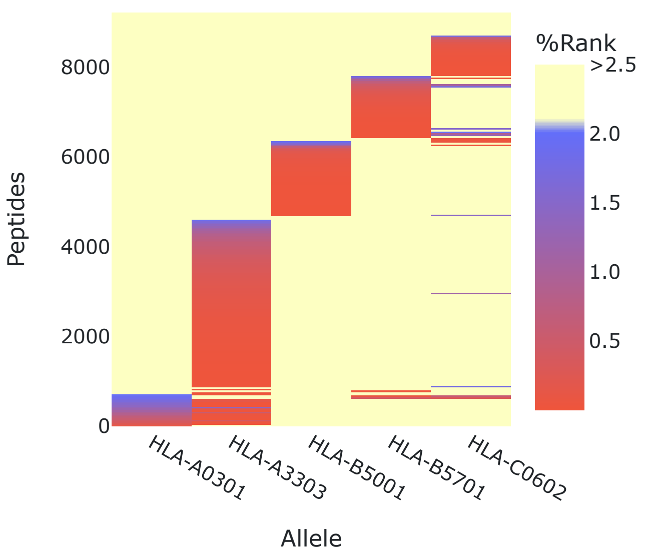

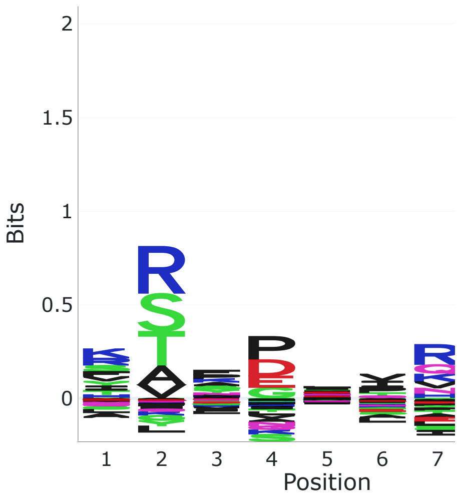

Peptides in group: 2617
HLA-A0301: 1%, HLA-A3303: 0%, HLA-B5001: 0%, **HLA-B5701: 49%,** HLA-C0602: 43%

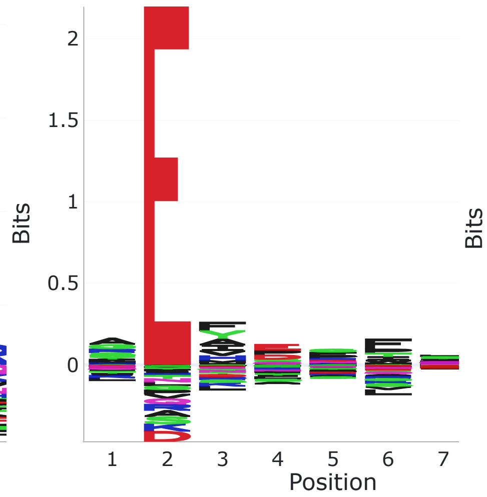

Peptides in group: 1624
HLA-A0301: 0%, HLA-A3303: 0%, **HLA-B5001: 92%,** HLA-B5701: 0%, HLA-C0602: 1%

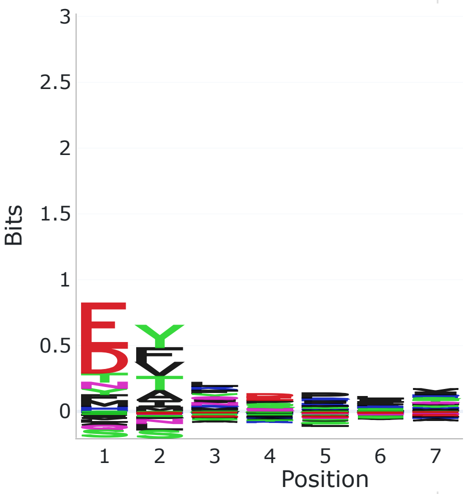

Peptides in group: 4749
HLA-A0301: 2%, **HLA-A3303: 77%,** HLA-B5001: 0%, HLA-B5701: 0%, HLA-C0602: 0%

DU145_ctrl_P651_2_peptide.tsv (peptides clustered: 9859, outliers: 265)

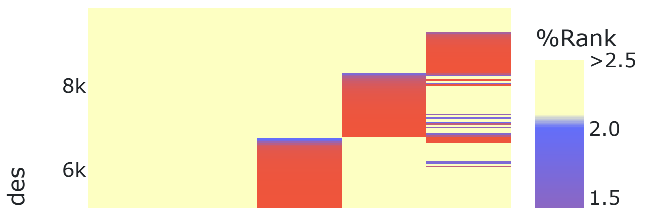

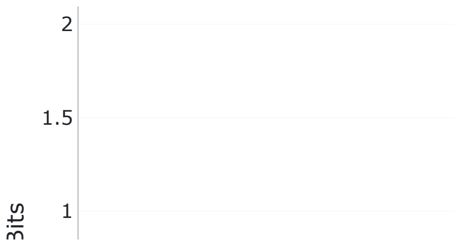

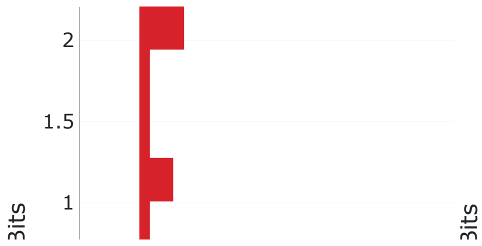

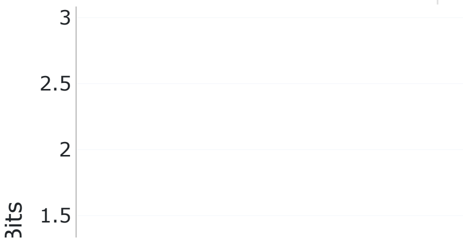

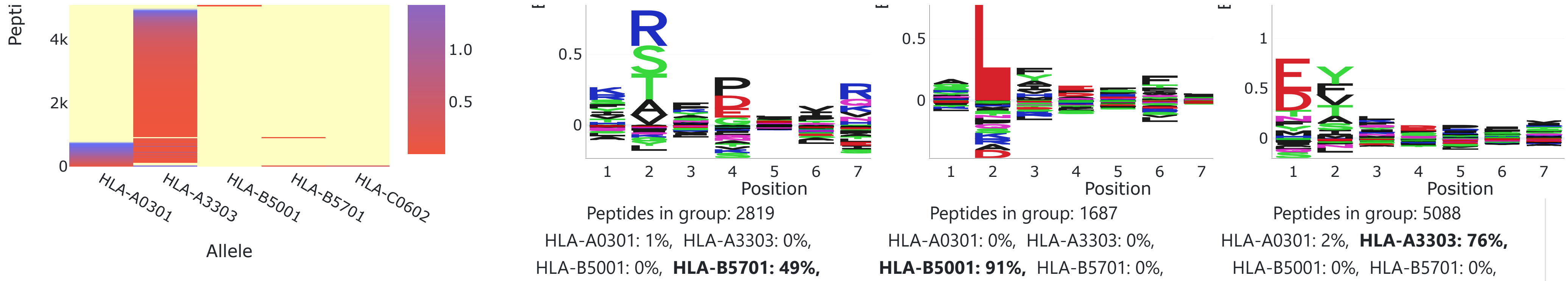

Peptides in group: 2819
HLA-A0301: 1%, HLA-A3303: 0%, HLA-B5001: 0%, **HLA-B5701: 49%,** HLA-C0602: 43%

Peptides in group: 1687
HLA-A0301: 0%, HLA-A3303: 0%, **HLA-B5001: 91%,** HLA-B5701: 0%, HLA-C0602: 1%

Peptides in group: 5088
HLA-A0301: 2%, **HLA-A3303: 76%,** HLA-B5001: 0%, HLA-B5701: 0%, HLA-C0602: 0%

DU145_etop_P651_1_peptide.tsv (peptides clustered: 7668, outliers: 514)

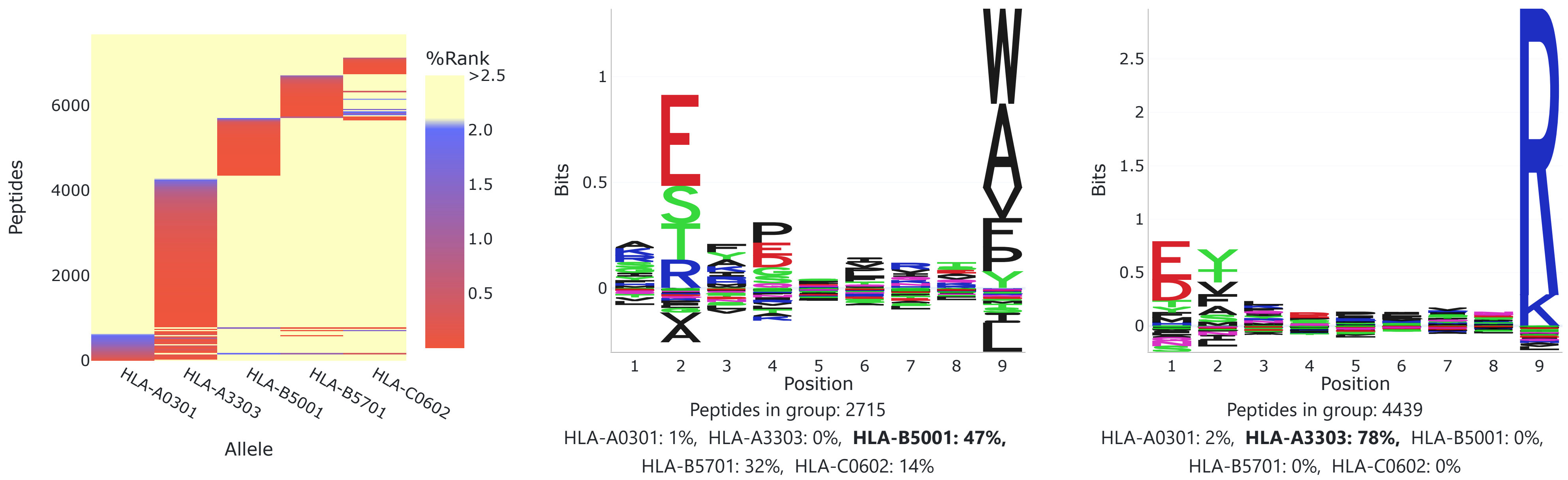

Peptides in group: 2715
HLA-A0301: 1%, HLA-A3303: 0%, **HLA-B5001: 47%,** HLA-B5701: 32%, HLA-C0602: 14%

Peptides in group: 4439
HLA-A0301: 2%, **HLA-A3303: 78%,** HLA-B5001: 0%, HLA-B5701: 0%, HLA-C0602: 0%

DU145_etop_P651_2_peptide.tsv (peptides clustered: 8453, outliers: 216)

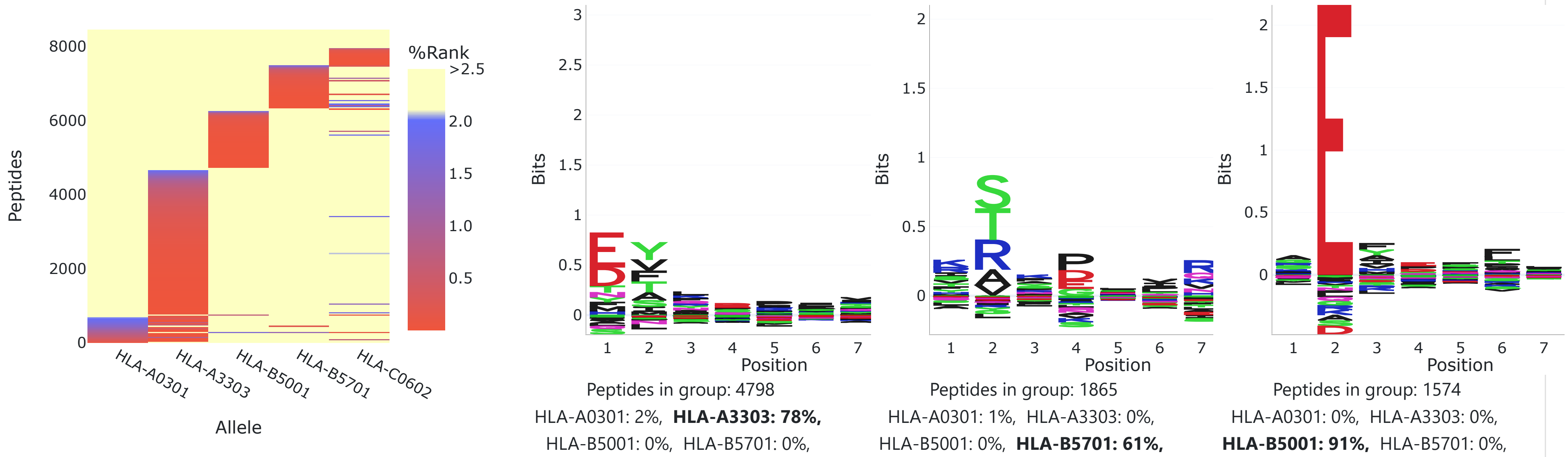

Peptides in group: 4798
HLA-A0301: 2%, **HLA-A3303: 78%,** HLA-B5001: 0%, HLA-B5701: 0%, HLA-C0602: 0%

Peptides in group: 1865
HLA-A0301: 1%, HLA-A3303: 0%, HLA-B5001: 0%, **HLA-B5701: 61%,** HLA-C0602: 33%

Peptides in group: 1574
HLA-A0301: 0%, HLA-A3303: 0%, **HLA-B5001: 91%,** HLA-B5701: 0%, HLA-C0602: 1%

DU145_etop_P651_3_peptide.tsv (peptides clustered: 8635, outliers: 271)

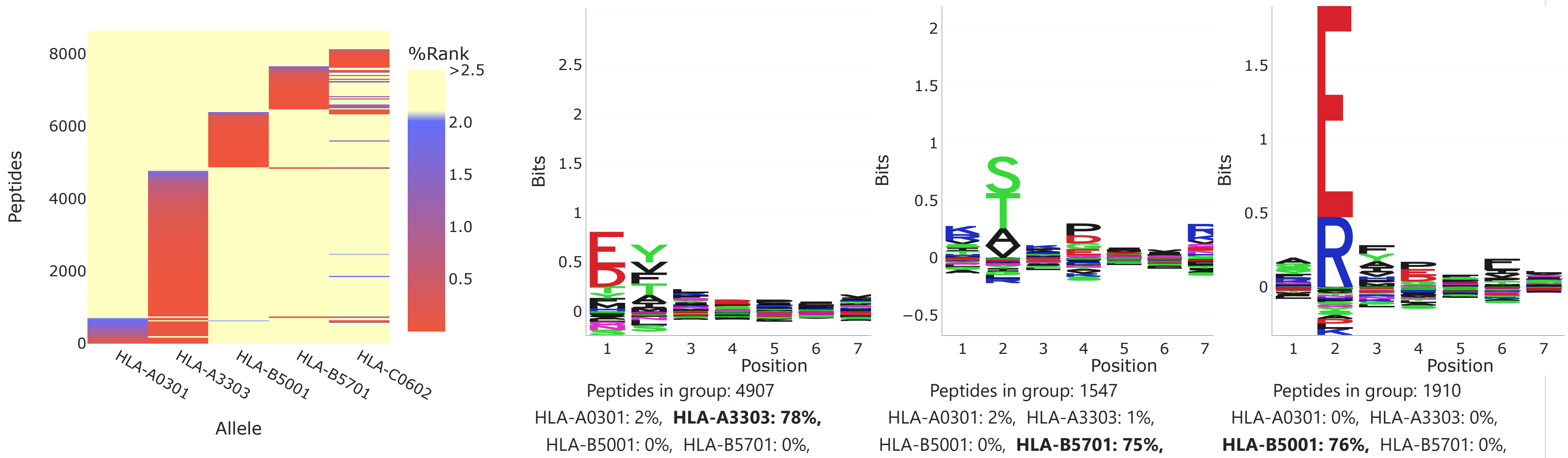

Peptides in group: 4907
HLA-A0301: 2%, **HLA-A3303: 78%,** HLA-B5001: 0%, HLA-B5701: 0%, HLA-C0602: 0%

Peptides in group: 1547
HLA-A0301: 2%, HLA-A3303: 1%, HLA-B5001: 0%, **HLA-B5701: 75%,** HLA-C0602: 15%

Peptides in group: 1910
HLA-A0301: 0%, HLA-A3303: 0%, **HLA-B5001: 76%,** HLA-B5701: 0%, HLA-C0602: 18%

Supplementary Figure S11 Quality control plots generated using MHCVizPipe from immunopeptidomics analysis from SNB19 cells.

MhcVizPipe (v0.7.9) - Analysis report

Date: 2024-01-02

Submitted by: Anonymous

Analysis type: Class I

Description of experiment:
None provided

Samples:

Snb19Ctrl_1_peptide.tsv:
Alleles: HLA-A2402, HLA-A0201, HLA-B1801, HLA-C0501
Snb19Ctrl_3_peptide.tsv:
Alleles: HLA-A2402, HLA-A0201, HLA-B1801, HLA-C0501
Snb19Ctrl_2_peptide.tsv:
Alleles: HLA-A2402, HLA-A0201, HLA-B1801, HLA-C0501
Snb19Etop_3_peptide.tsv:
Alleles: HLA-A2402, HLA-A0201, HLA-B1801, HLA-C0501
Snb19Etop_2_peptide.tsv:
Alleles: HLA-A2402, HLA-A0201, HLA-B1801, HLA-C0501
Snb19Etop_1_peptide.tsv:
Alleles: HLA-A2402, HLA-A0201, HLA-B1801, HLA-C0501

Sample Overview

- LF Score: fraction of peptides between 8 and 12 mers.
- BF Score: fraction of peptides between 8 and 12 mers which are predicted to be strong or weak binders.

Sample	Total peptides	Peptides between 8-12 mers	LF Score	BF Score
Snb19Ctrl_1_peptide.tsv	8550	8130	0.95	0.94
Snb19Ctrl_3_peptide.tsv	9701	9203	0.95	0.94
Snb19Ctrl_2_peptide.tsv	9613	9117	0.95	0.94
Snb19Etop_3_peptide.tsv	7791	7332	0.94	0.94
Snb19Etop_2_peptide.tsv	7773	7352	0.95	0.94
Snb19Etop_1_peptide.tsv	8354	7873	0.94	0.94

UpSet Plot (only displaying intersections containing >= 1% of at least one sample)

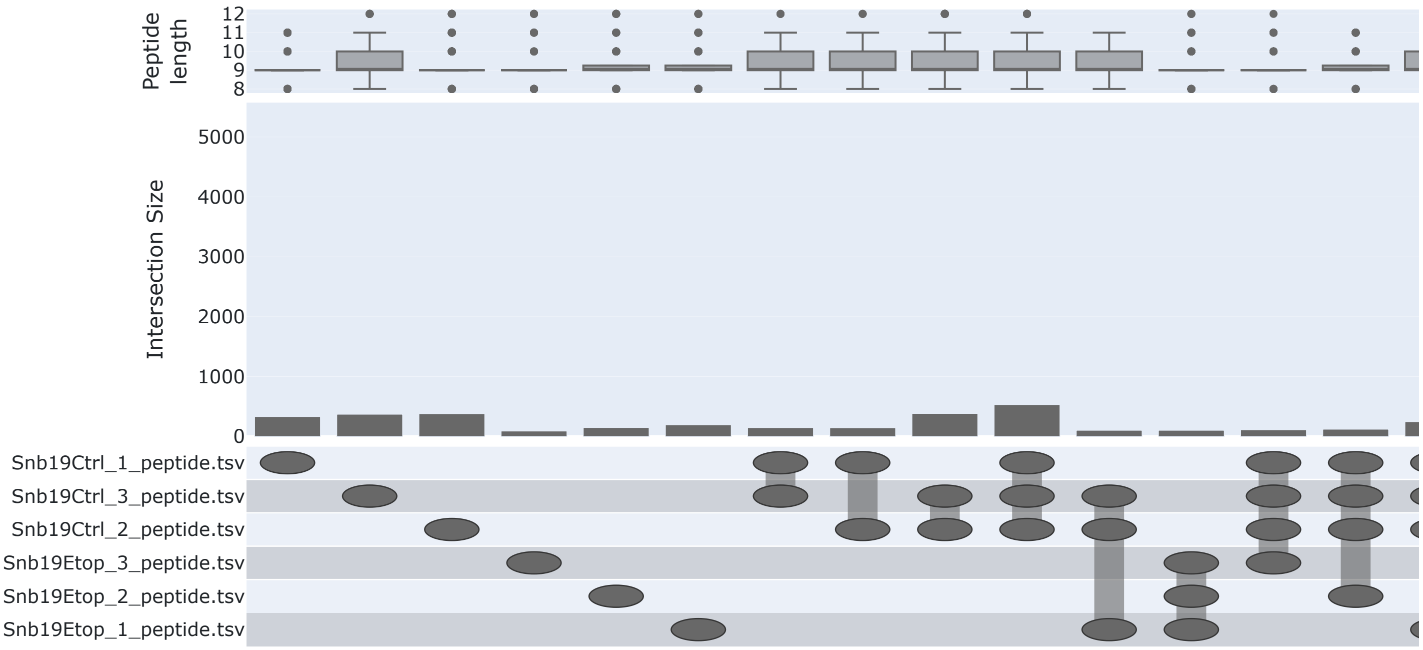

Peptide Length Distribution (maximum of 30 mers)

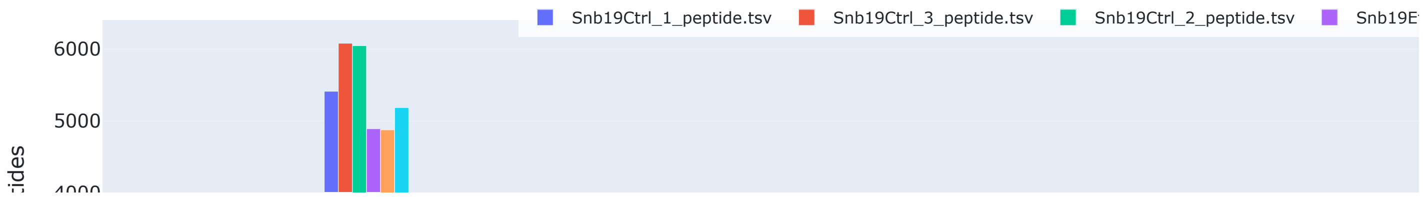

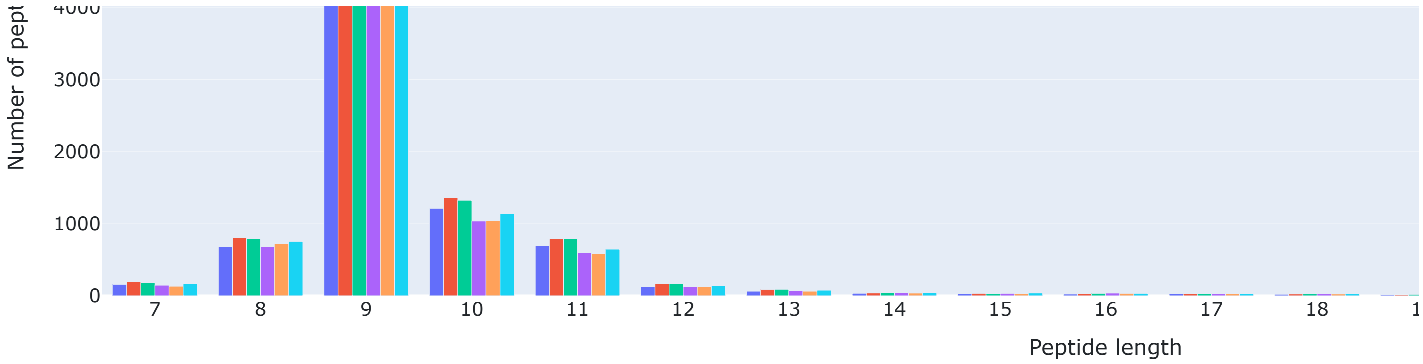

Annotation Results

NetMHCpan eluted ligand predictions made for all peptides between 8 & 12 mers, inclusive.
 - Percent rank cutoffs for strong and weak binders: 0.5 and 2.0.
 - Percentages are calculated across rows (i.e. percentage of total peptides for a respective sample).

Sample	Total peptides	Allele	Strong binders	Weak binders	Non-binders
Snb19Ctrl_1_peptide.tsv	8130	HLA-A2402	3616 (44.5%)	653 (8.0%)	3861 (47.5%)
		HLA-A0201	2520 (31.0%)	592 (7.3%)	5018 (61.7%)
		HLA-B1801	504 (6.2%)	473 (5.8%)	7153 (88.0%)
		HLA-C0501	1015 (12.5%)	1444 (17.8%)	5671 (69.8%)
Snb19Ctrl_3_peptide.tsv	9203	HLA-A2402	3952 (42.9%)	740 (8.0%)	4511 (49.0%)
		HLA-A0201	2942 (32.0%)	685 (7.4%)	5576 (60.6%)
		HLA-B1801	580 (6.3%)	504 (5.5%)	8119 (88.2%)
		HLA-C0501	1159 (12.6%)	1663 (18.1%)	6381 (69.3%)
Snb19Ctrl_2_peptide.tsv	9117	HLA-A2402	3879 (42.5%)	723 (7.9%)	4515 (49.5%)
		HLA-A0201	2932 (32.2%)	696 (7.6%)	5489 (60.2%)
		HLA-B1801	582 (6.4%)	491 (5.4%)	8044 (88.2%)
		HLA-C0501	1154 (12.7%)	1659 (18.2%)	6304 (69.1%)
Snb19Etop_3_peptide.tsv	7332	HLA-A2402	3216 (43.9%)	542 (7.4%)	3574 (48.7%)
		HLA-A0201	2352 (32.1%)	440 (6.0%)	4540 (61.9%)
		HLA-B1801	603 (8.2%)	417 (5.7%)	6312 (86.1%)
		HLA-C0501	852 (11.6%)	1312 (17.9%)	5168 (70.5%)
Snb19Etop_2_peptide.tsv	7352	HLA-A2402	3074 (41.8%)	559 (7.6%)	3719 (50.6%)
		HLA-A0201	2449 (33.3%)	462 (6.3%)	4441 (60.4%)
		HLA-B1801	623 (8.5%)	411 (5.6%)	6318 (85.9%)
		HLA-C0501	871 (11.8%)	1343 (18.3%)	5138 (69.9%)
Snb19Etop_1_peptide.tsv	7873	HLA-A2402	3332 (42.3%)	605 (7.7%)	3936 (50.0%)
		HLA-A0201	2559 (32.5%)	494 (6.3%)	4820 (61.2%)
		HLA-B1801	655 (8.3%)	448 (5.7%)	6770 (86.0%)
		HLA-C0501	911 (11.6%)	1413 (17.9%)	5549 (70.5%)

Binding Affinities

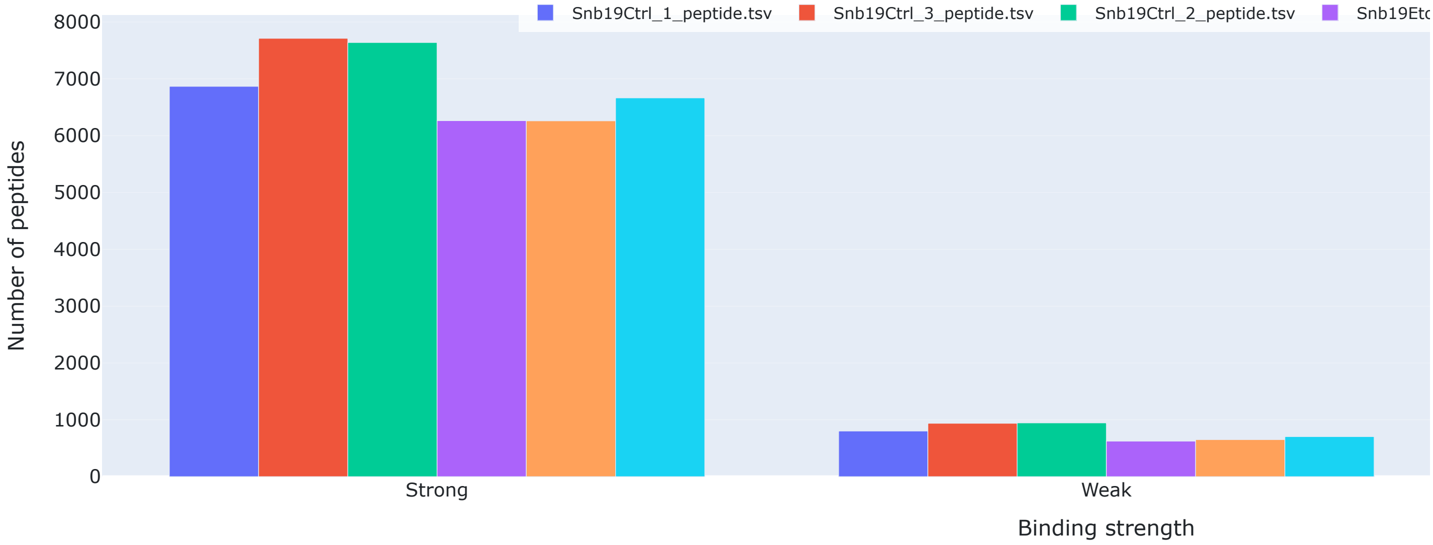

Binding Heatmaps

NetMHCpan eluted ligand predictions made for all peptides between 8 & 12 mers, inclusive.
Approximate color legend (detailed mapping shown next to heatmaps):

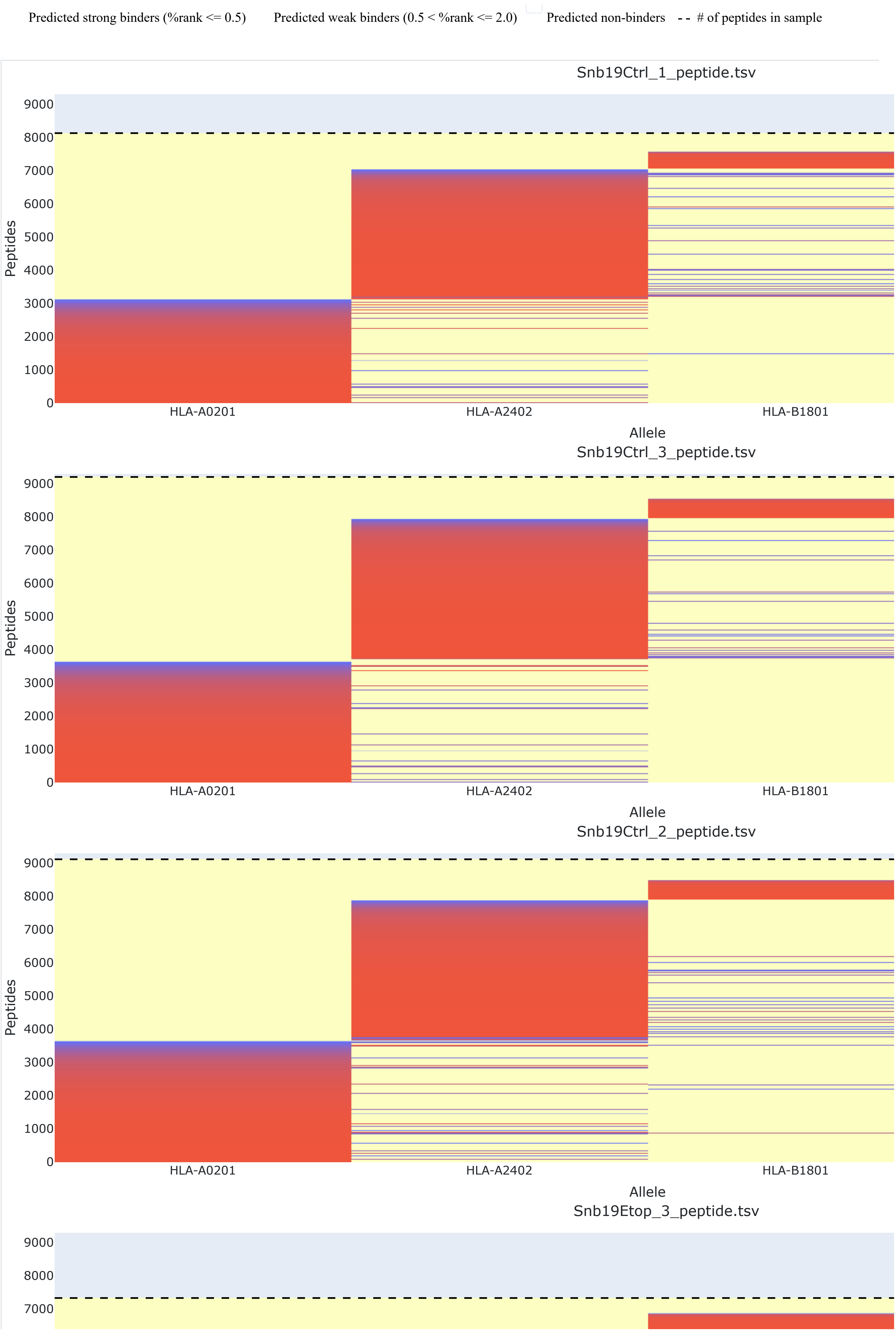

Predicted strong binders (%rank <= 0.5)
Predicted weak binders (0.5 < %rank <= 2.0)
Predicted non-binders
- - # of peptides in sample
Snb19Ctrl_1_peptide.tsv
Peptides
Allele
HLA-A0201
HLA-A2402
HLA-B1801
Snb19Ctrl_3_peptide.tsv
Snb19Ctrl_2_peptide.tsv
Snb19Etop_3_peptide.tsv
0
1000
2000
3000
4000
5000
6000
7000
8000
9000

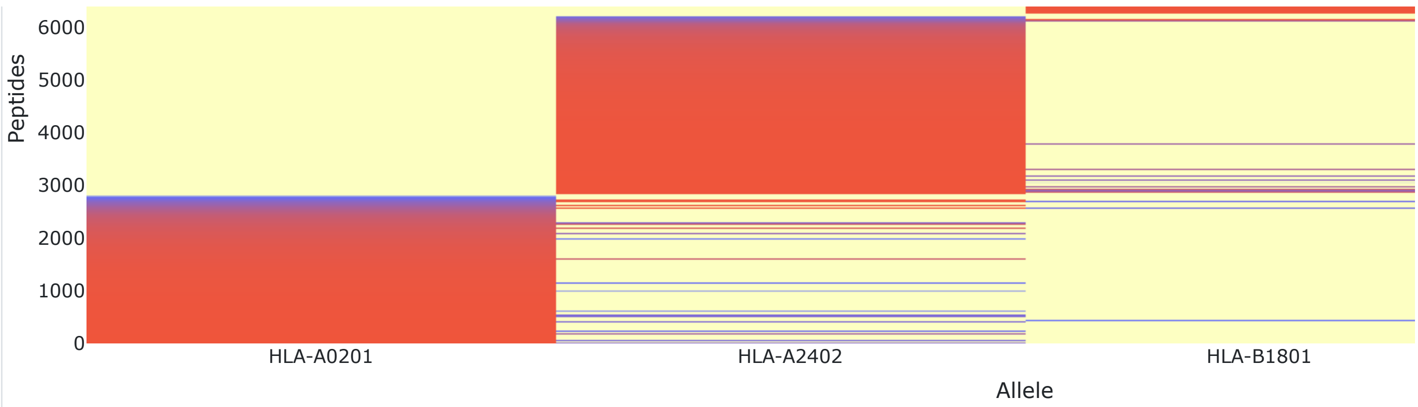

Snb19Etop_2_peptide.tsv

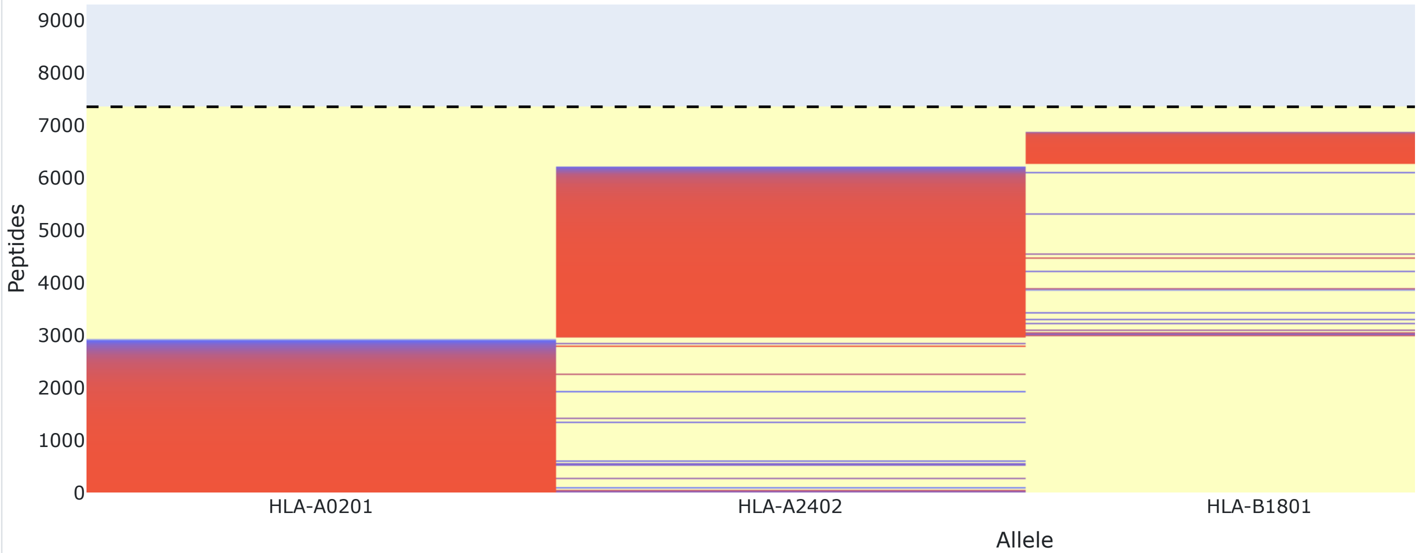

Snb19Etop_1_peptide.tsv

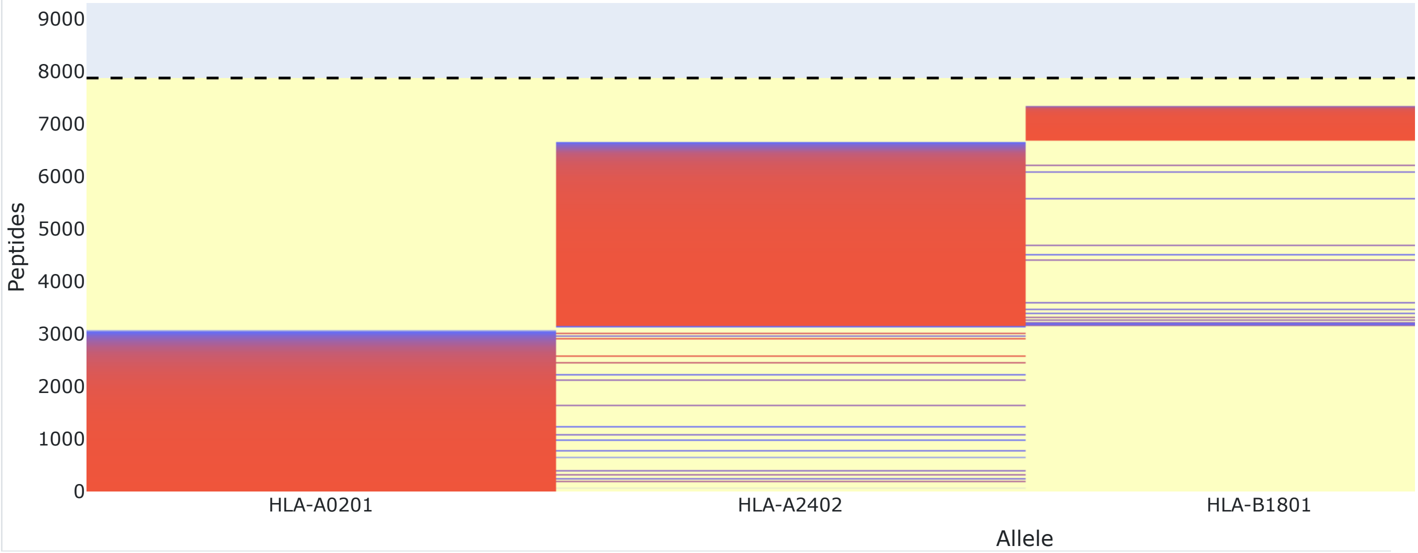

Sequence Motifs

Clustering performed with all peptides between 8 & 12 mers, inclusive.

- Percentages represent the percentage of peptides in a given group predicted to strongly bind the indicated allele.

Polar Neutral Basic Acidic Hydrophobic

- Unsupervised GibbsCluster
- Allele-specific GibbsCluster

Snb19Ctrl_1_peptide.tsv (peptides clustered: 8130, outliers: 519)

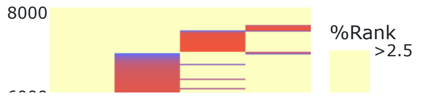

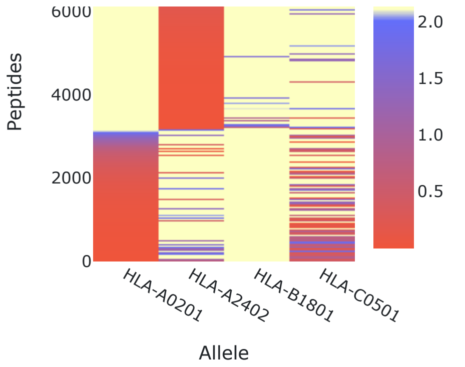

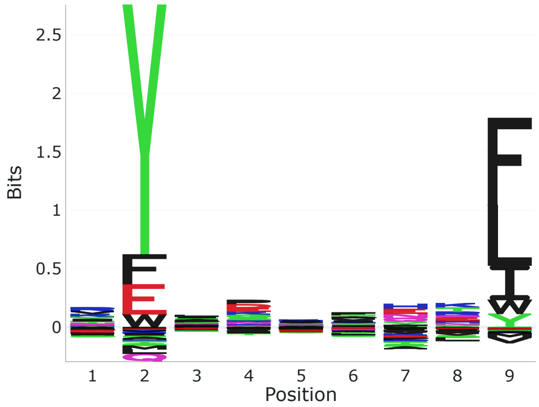

Peptides in group: 4299
HLA-A2402: 83%, HLA-A0201: 0%, HLA-B1801: 7%, HLA-C0501: 2%

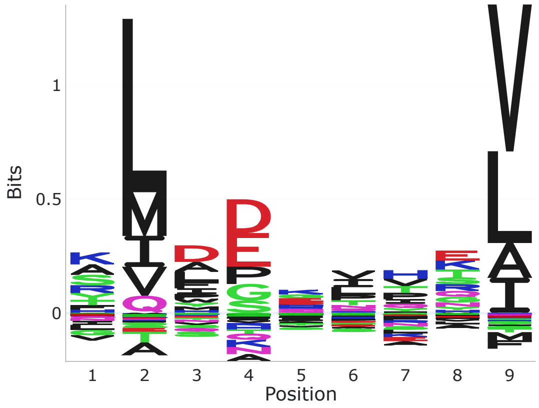

Peptides in group: 3312
HLA-A2402: 1%, **HLA-A0201: 75%,** HLA-B1801: 0%, HLA-C0501: 27%

Snb19Ctrl_3_peptide.tsv (peptides clustered: 9203, outliers: 572)

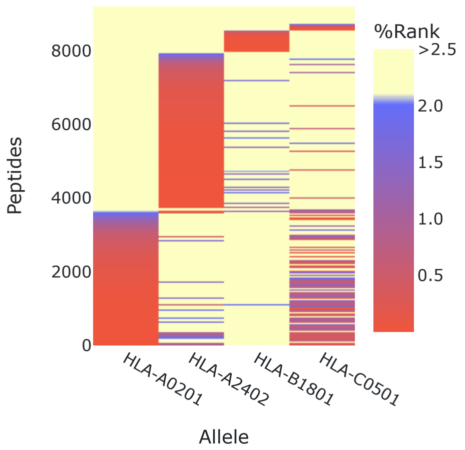

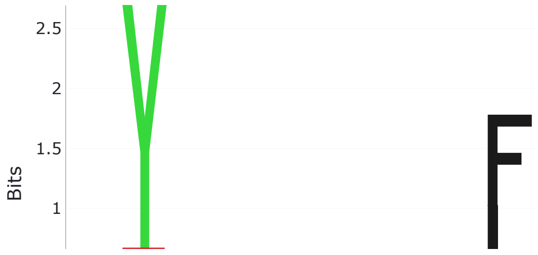

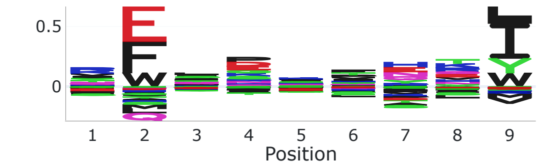

Peptides in group: 4766
HLA-A2402: 81%, HLA-A0201: 0%, HLA-B1801: 7%, HLA-C0501: 2%

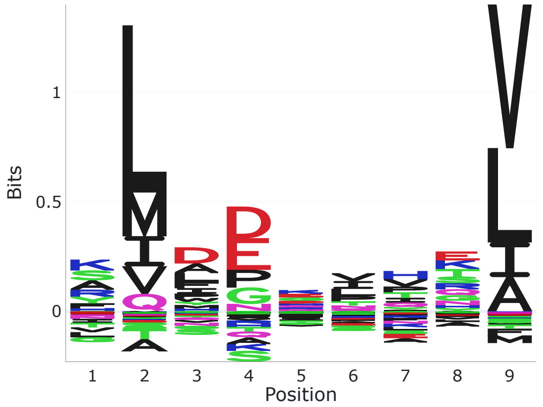

Peptides in group: 3865
HLA-A2402: 2%, **HLA-A0201: 75%,** HLA-B1801: 0%, HLA-C0501: 26%

Snb19Ctrl_2_peptide.tsv (peptides clustered: 9117, outliers: 566)

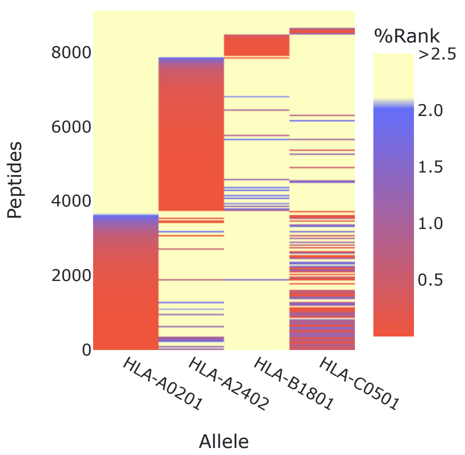

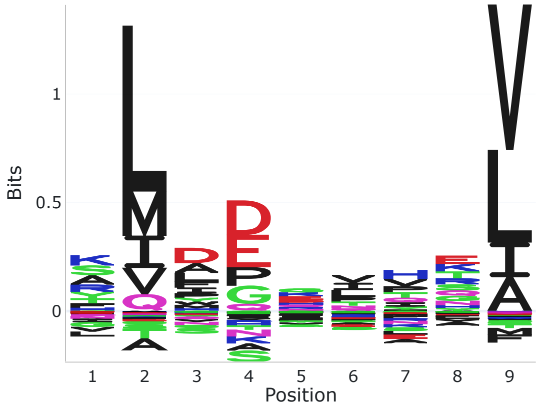

Peptides in group: 3872
HLA-A2402: 2%, **HLA-A0201: 75%,** HLA-B1801: 0%, HLA-C0501: 26%

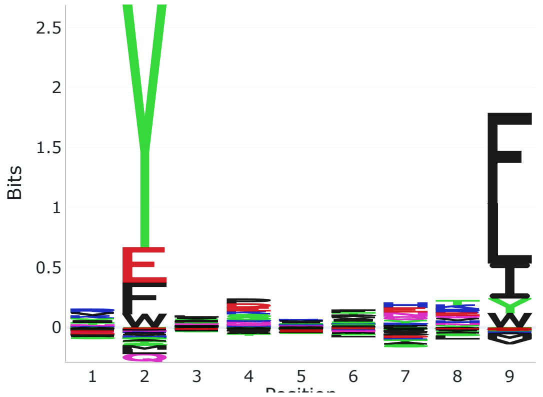

Position
Peptides in group: 4679
HLA-A2402: 81%, HLA-A0201: 0%, HLA-B1801: 8%, HLA-C0501: 2%

Snb19Etop_3_peptide.tsv (peptides clustered: 7332, outliers: 425)

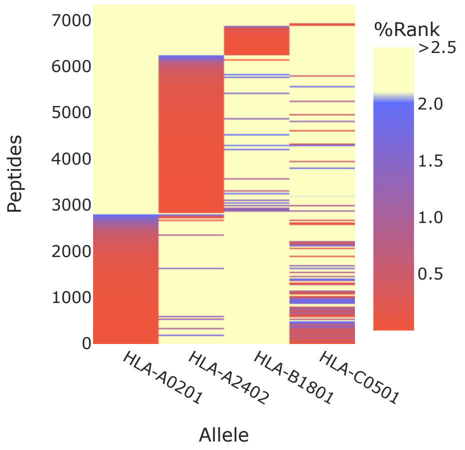

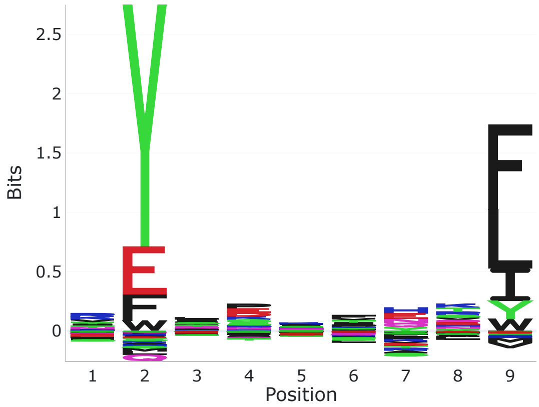

Peptides in group: 3944
HLA-A2402: 80%, HLA-A0201: 0%, HLA-B1801: 11%, HLA-C0501: 2%

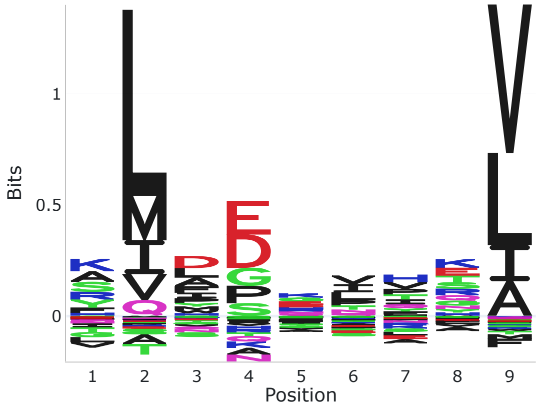

Peptides in group: 2963
HLA-A2402: 2%, **HLA-A0201: 79%,** HLA-B1801: 0%, HLA-C0501: 26%

Snb19Etop_2_peptide.tsv (peptides clustered: 7352, outliers: 230)

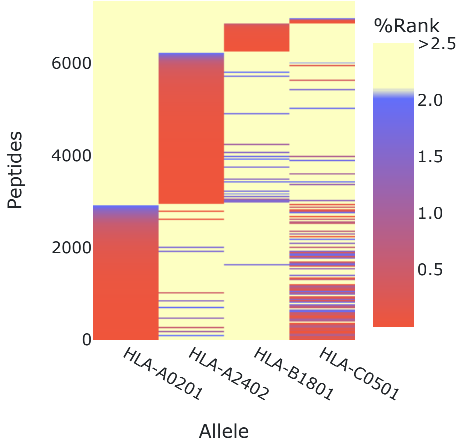

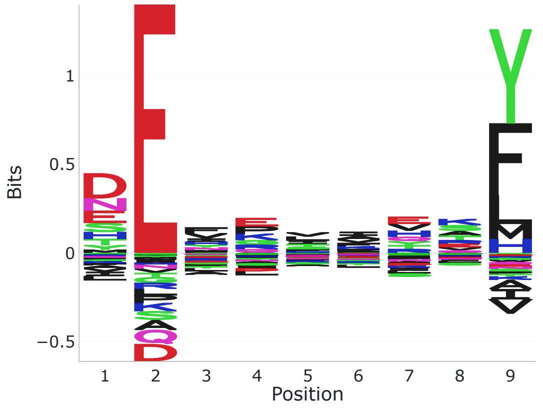

Peptides in group: 755
HLA-A2402: 2%, HLA-A0201: 0%, **HLA-B1801: 82%,**
HLA-C0501: 1%

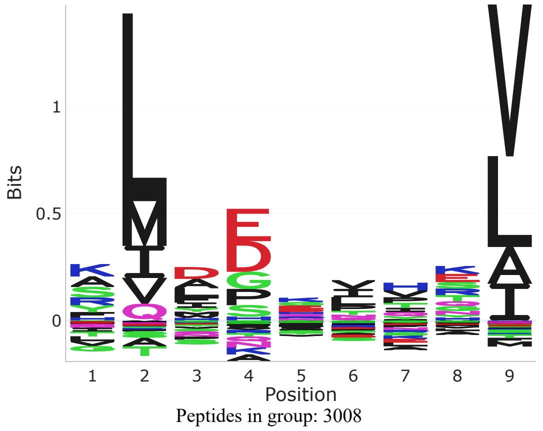

Peptides in group: 3008
HLA-A2402: 1%, **HLA-A0201: 80%,** HLA-B1801: 0%,
HLA-C0501: 25%

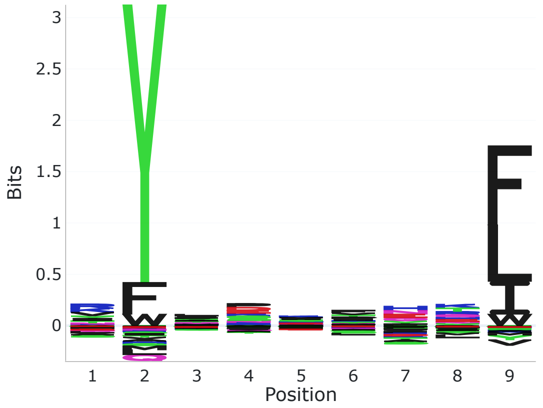

Peptides in group: 3359
HLA-A2402: 90%, HLA-A0201: 1%, HLA-B1801: 0%,
HLA-C0501: 2%

Snb19Etop_1_peptide.tsv (peptides clustered: 7873, outliers: 491)

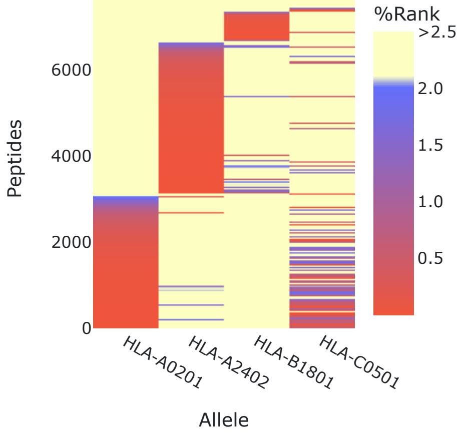

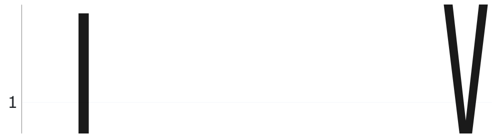

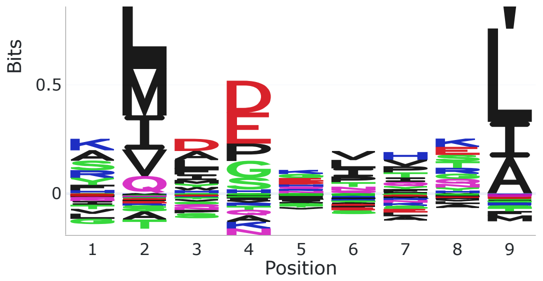

Peptides in group: 3218
HLA-A2402: 1%, **HLA-A0201: 79%,** HLA-B1801: 0%, HLA-C0501: 25%

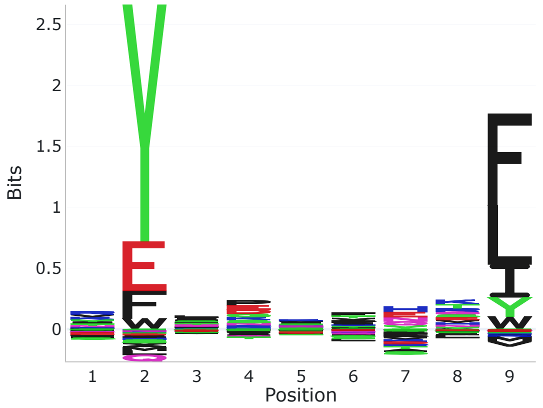

Peptides in group: 4164
HLA-A2402: 79%, HLA-A0201: 0%, HLA-B1801: 12%, HLA-C0501: 2%

Snb19Ctrl_1_peptide.tsv sequence motif(s)

HLA-A2402

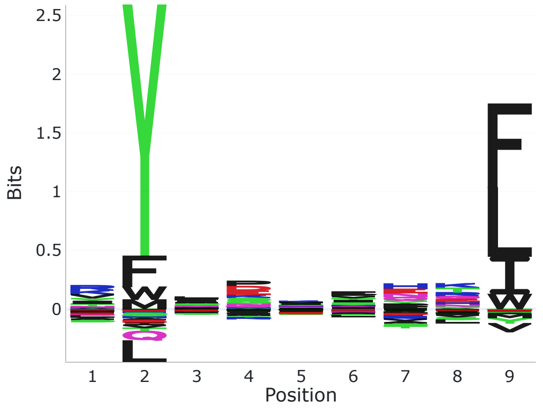

Peptides: 4228

HLA-A0201

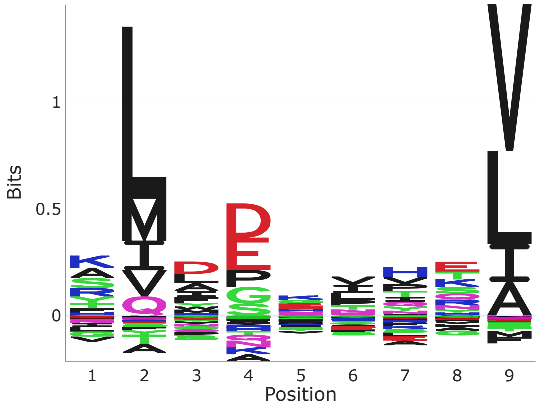

Peptides: 3057

HLA-B1801

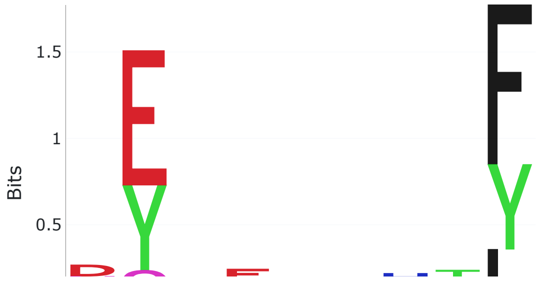

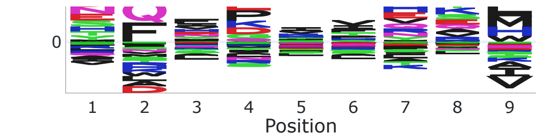

Peptides: 969

HLA-C0501

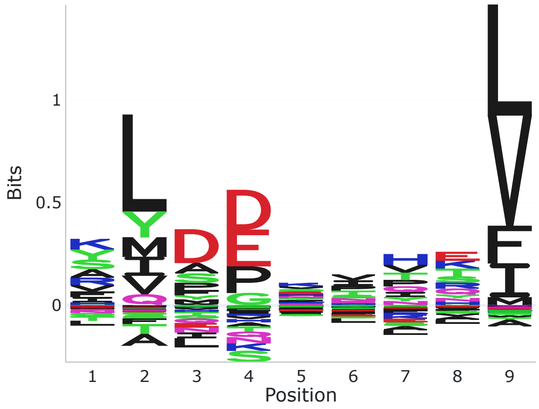

Peptides: 2416

Non-binders group 1

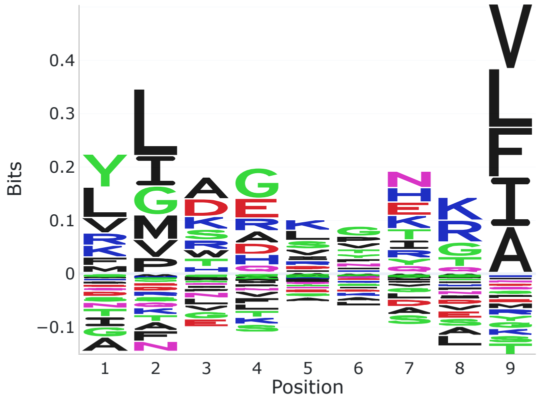

Peptides: 230

Non-binders group 2

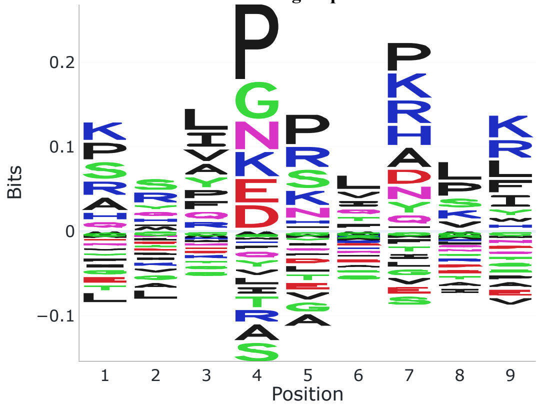

Peptides: 135

Snb19Ctrl_3_peptide.tsv sequence motif(s)

HLA-A2402

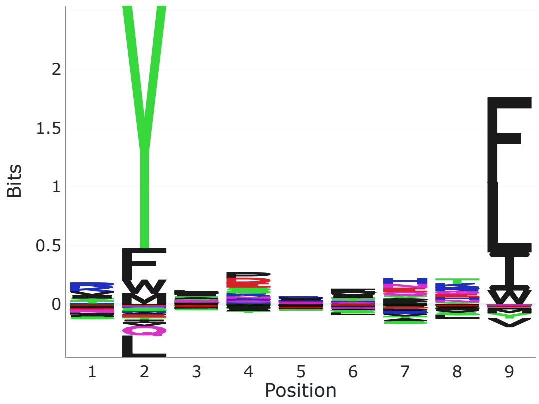

Peptides: 4640

HLA-A0201

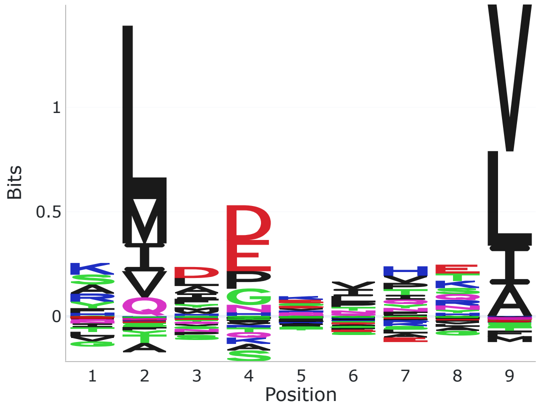

Peptides: 3574

HLA-B1801

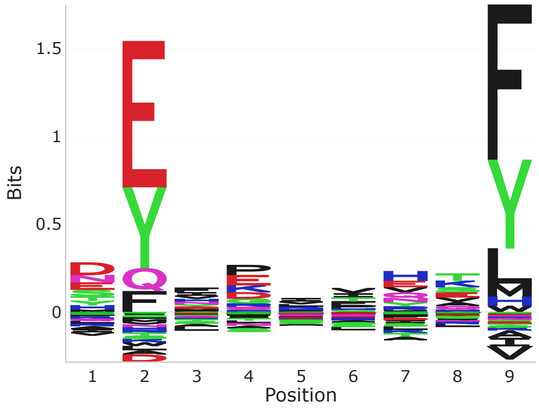

Peptides: 1078

HLA-C0501

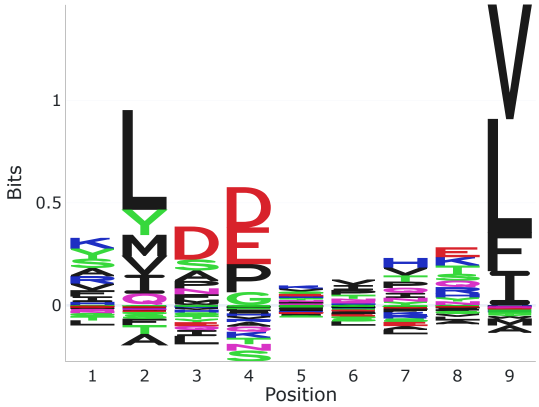

Peptides: 2769

Non-binders group 1

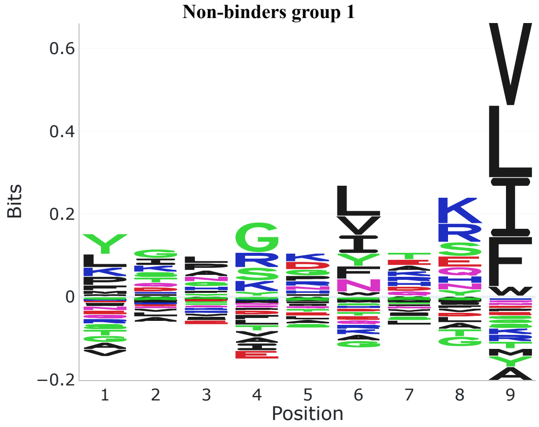

Peptides: 251

Non-binders group 2

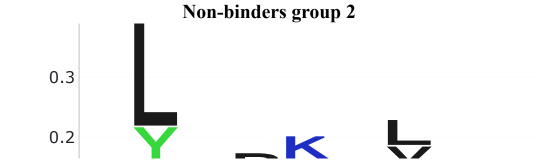

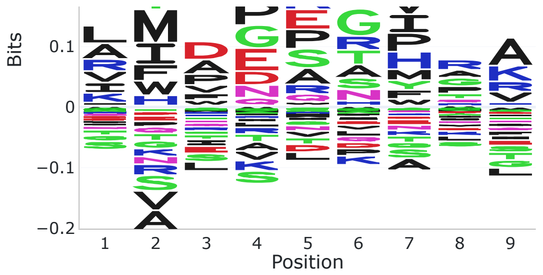

Peptides: 193

Snb19Ctrl_2_peptide.tsv sequence motif(s)

HLA-A2402

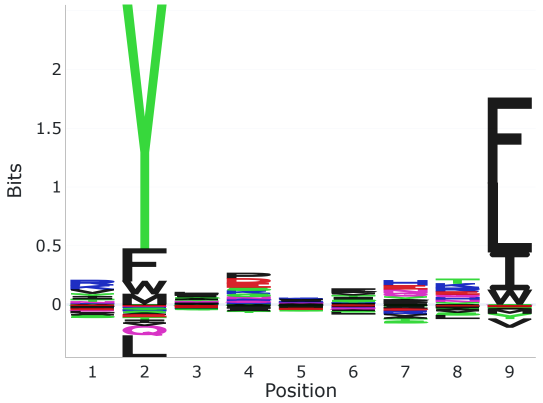

Peptides: 4557

HLA-A0201

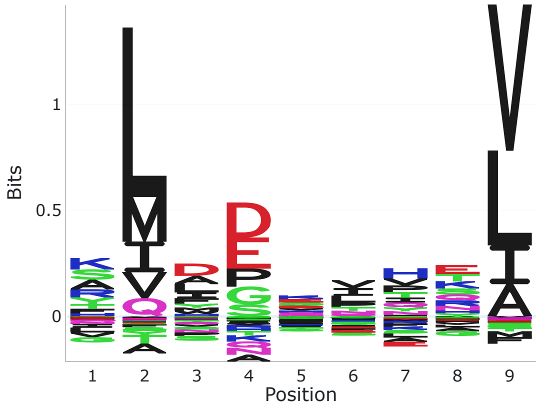

Peptides: 3569

HLA-B1801

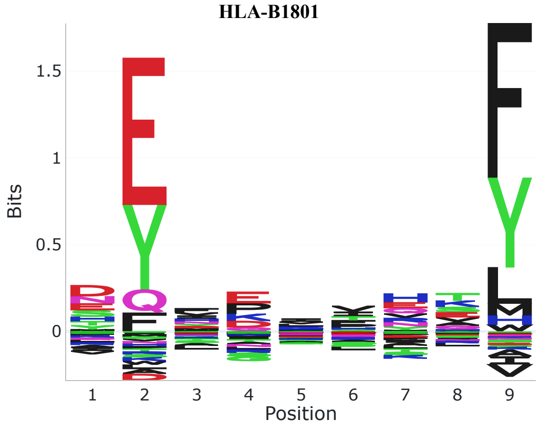

Peptides: 1066

HLA-C0501

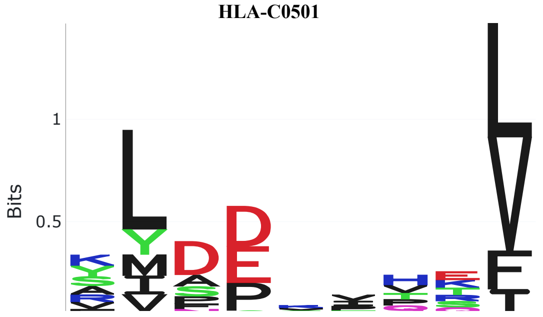

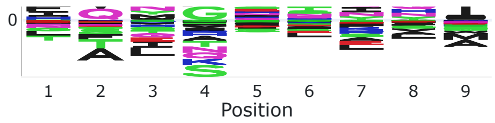

Peptides: 2760

Non-binders group 1

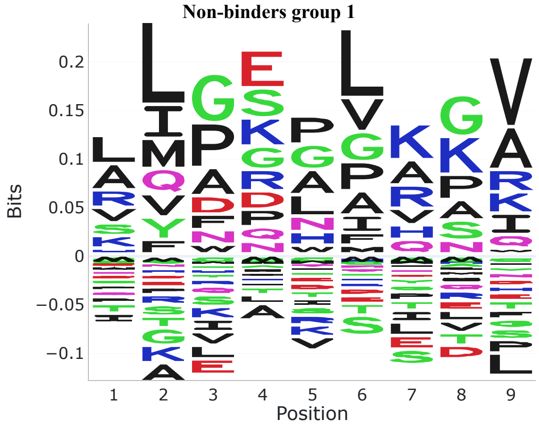

Peptides: 219

Non-binders group 2

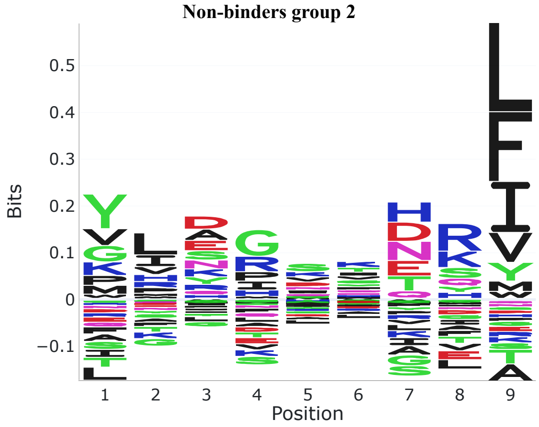

Peptides: 207

Snb19Etop_3_peptide.tsv sequence motif(s)

HLA-A2402

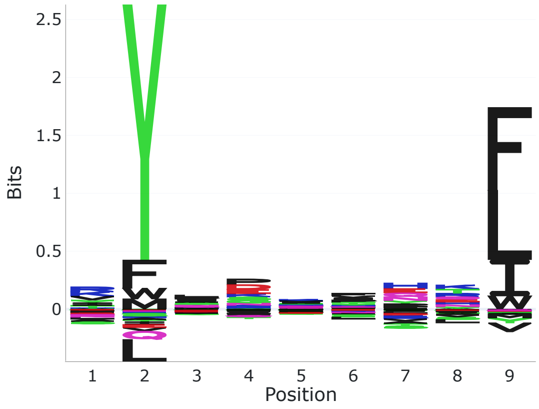

Peptides: 3718

HLA-A0201

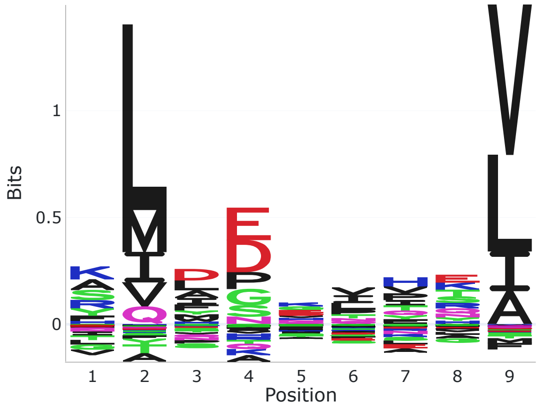

Peptides: 2760

HLA-B1801

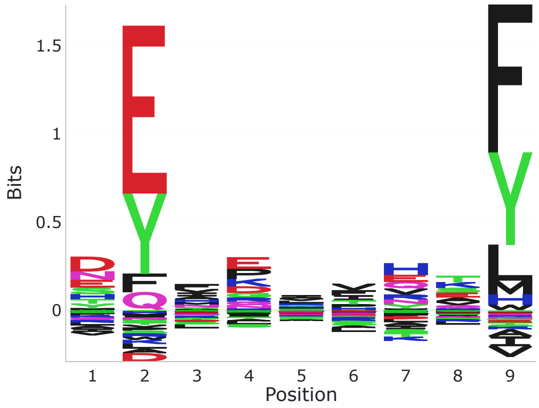

Peptides: 1015

HLA-C0501

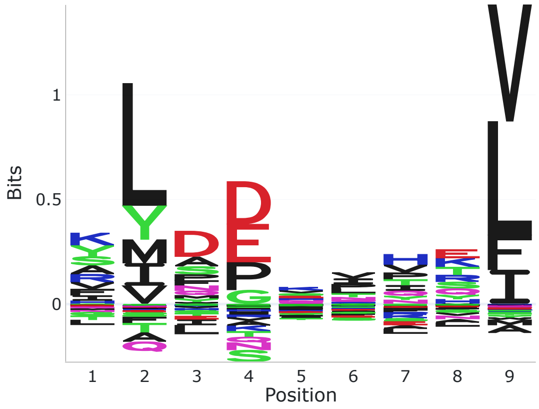

Peptides: 2135

Non-binders group 1

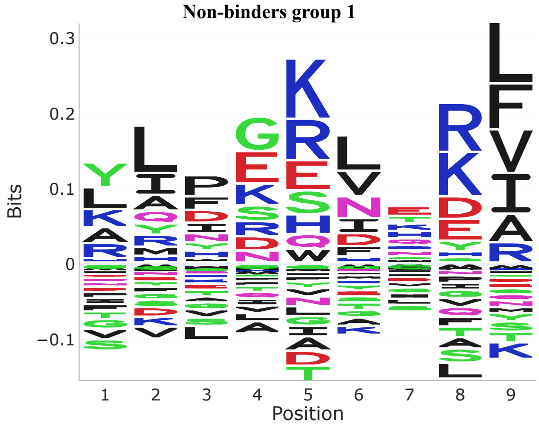

Peptides: 182

Non-binders group 2

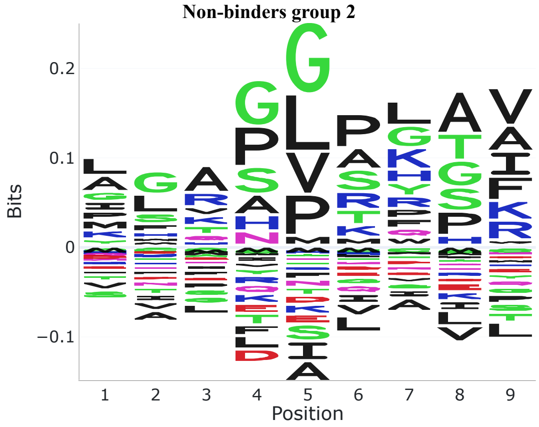

Peptides: 170

Snb19Etop_2_peptide.tsv sequence motif(s)

HLA-A2402

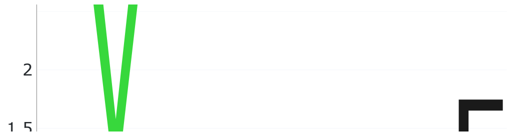

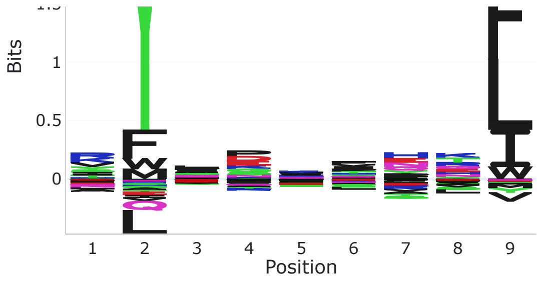

Peptides: 3600

HLA-A0201

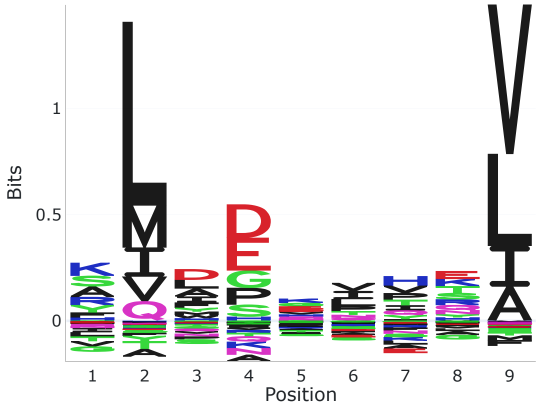

Peptides: 2869

HLA-B1801

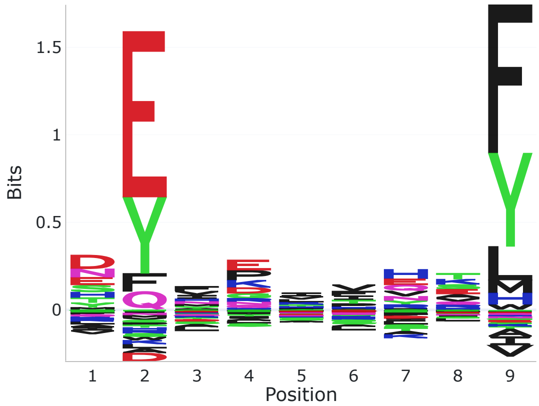

Peptides: 1029

HLA-C0501

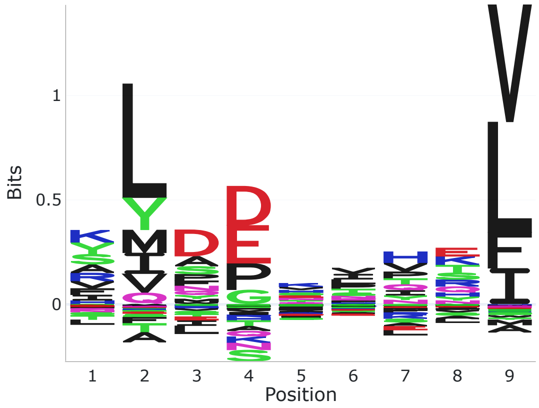

Peptides: 2185

Non-binders group 1

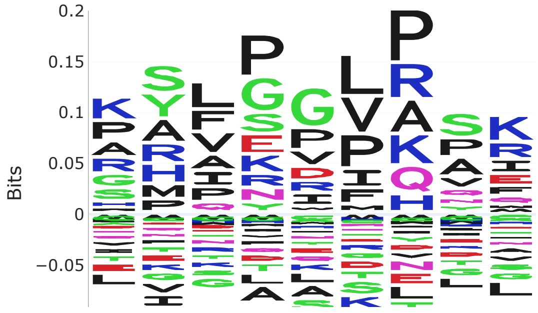

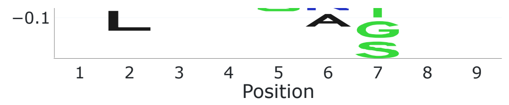

Peptides: 143

Non-binders group 2

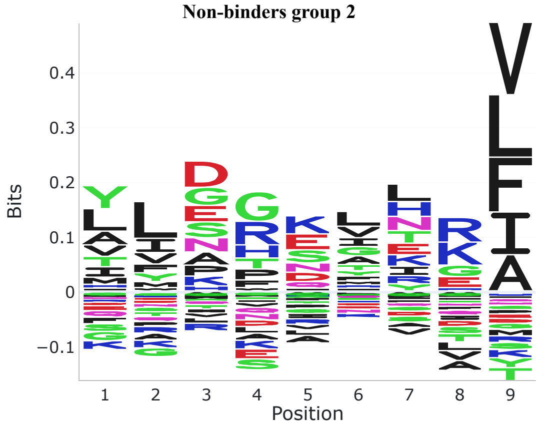

Peptides: 205

Snb19Etop_1_peptide.tsv sequence motif(s)

HLA-A2402

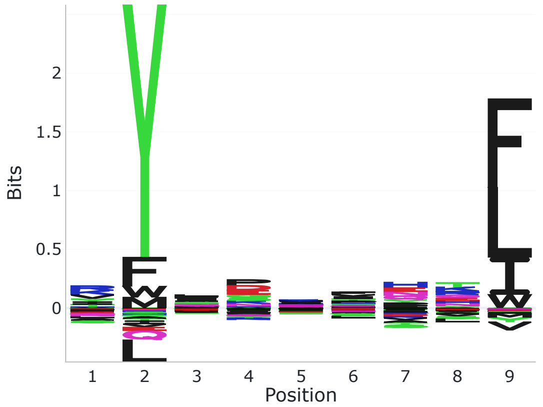

Peptides: 3896

HLA-A0201

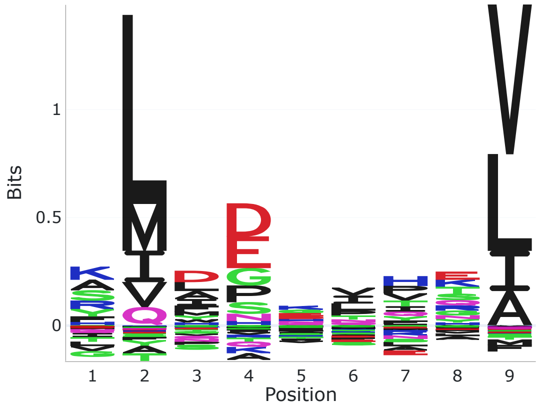

Peptides: 3014

HLA-B1801

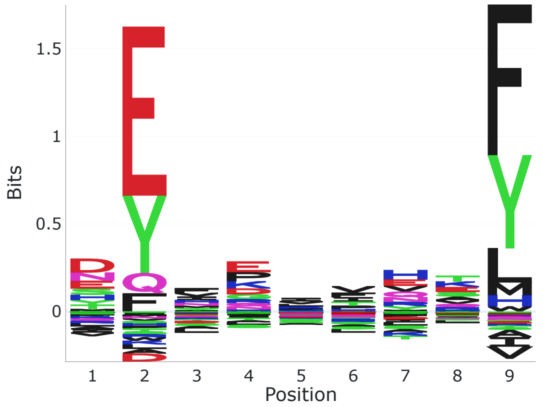

Peptides: 1096

HLA-C0501

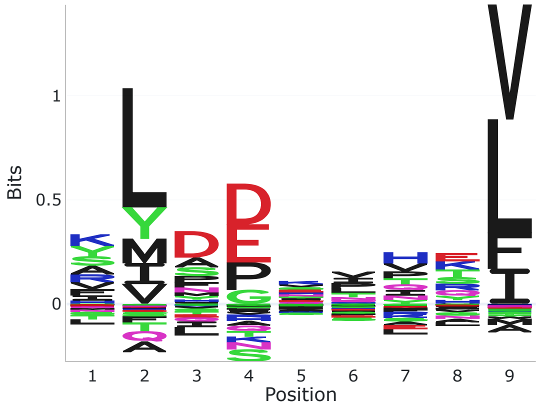

Peptides: 2289

Non-binders group 1

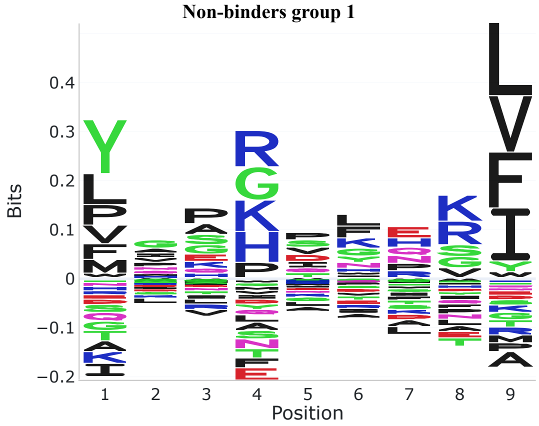

Peptides: 202

Non-binders group 2

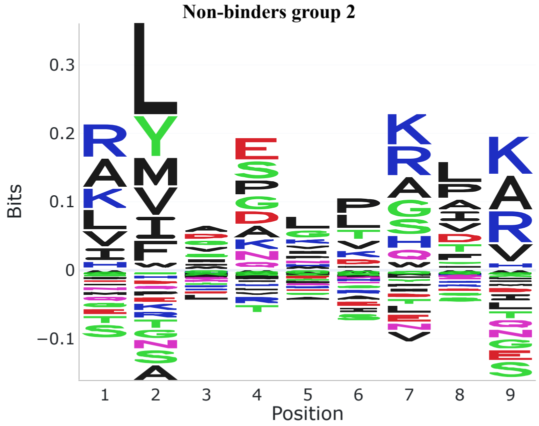

Peptides: 186

Supplementary Table S1. DNA sequences used for cloning.

Oligo name	Sequence (5' to 3')
splt sfGFP(β1-10)-spacer-(β11)	TATATTCTAGAGCCACCATGGTGAGCAAGGGCGAGGAGCTGTTCACCGGGGTGGTGCCCATCCTGGTCGAGCTGGACGGCGACGTAAACGGCCACAAGTTCAGCGTGCGCGGCGAGGGCGAGGGCGATGCCACCAACGGCAAGCTGACCCTGAAGTTCATCTGCACCACCGGCAAGCTGCCCGTGCCCTTCCCCACCCTCGTGACCACCCTGACCTACGGCGTGCAGTGCTTCAGCCGCTACCCCGACCACATGAAGCGCCACGACTTCTTCAAGTCCGCCATGCCCGAAGGCTACGTCCAGGAGCGCACCATCAGCTTCAAGGACGACGGCACCTACAAGACCCGCGCCGAGGTGAAGTTCGAGGGCGACACCCTGGTGAACCGCATCGAGCTGAAGGGCATCGACTTCAAGGAGGACGGCAACATCCTGGGGCACAAGCTGGAGTACAACTTCAACAGCCACAACGTCTATATCACCGCCGACAAGCAGAAGAACGGCATCAAGGCCAACTTCAAGATCCGCCACAACGTGGAGGACGGCAGCGTGCAGCTCGCCGACCACTACCAGCAGAACACCCCCATCGGCGACGGCCCCGTGCTGCTGCCCGACAACCACTACCTGAGCACCCAGTCCGTGCTGAGCAAAGACCCCAACGAGGACGTTGGTGGTGGCGGATCAGAAGGAGGCGGTAGCGGGGGCCCTGGTTCGGGAGGGGAAGGTTCTGCTGGGGGAGGGAGCGCTGGCGGGGGGTCTAAGCGCGATCACATGGTCCTGCTGGAGTTCGTGACCGCCGCCGGGATCACTCACTAAGCGGCCGCATATT
NheI-V5-split sfGFP fw	ACAGTGGCTAGCGAATTCGGATCCGCCACCATGGGTAAGCCTATCCCTAACCCTCTCCTCGGTCTCGATTCTACGGGCGGCATGGTGAGCAAGGGCGAGG
NotI-split sfGFP Frame rev	AGAATTGCGGCCGCGTTTAAACTTAGTGAGTGATCCCGGCGGCGGTCACGAACTCCAGCAGGACCATGTGATCGCGCTTTCCCCTGTATAACCATAACCATTCAGACCCCCCGCCAGCGCTCC
NotI-split sfGFP UUA+1 rev	AGAATTGCGGCCGCGTTTAAACTTAGTGAGTGATCCCGGCGGCGGTCACGAACTCCAGCAGGACCATGTGATCGCGCTTTCCCCCTGTATAACCATAACCATTCAGACCCCCCGCCAGCGCTCC
NotI-split sfGFP UUA-1 rev	AGAATTGCGGCCGCGTTTAAACTTAGTGAGTGATCCCGGCGGCGGTCACGAACTCCAGCAGGACCATGTGATCGCGCTTTCCCTGTATAACCATAACCATTCAGACCCCCCGCCAGCGCTCC
NotI-split sfGFP UUG+1 rev	AGAATTGCGGCCGCGTTTAAACTTAGTGAGTGATCCCGGCGGCGGTCACGAACTCCAGCAGGACCATGTGATCGCGCTTTCCCCCTGTACAACCACAACCATTCAGACCCCCCGCCAGCGCTCC
NotI-split sfGFP CUA+1 rev	AGAATTGCGGCCGCGTTTAAACTTAGTGAGTGATCCCGGCGGCGGTCACGAACTCCAGCAGGACCATGTGATCGCGCTTTCCCCCTGTATAGCCATA
NotI-split sfGFP CUC+1 rev	AGAATTGCGGCCGCGTTTAAACTTAGTGAGTGATCCCGGCGGCGGTCACGAACTCCAGCAGGACCATGTGATCGCGCTTTCCCCCTGTAGAGCCAG
NotI-split sfGFP CUC-1 rev	AGAATTGCGGCCGCGTTTAAACTTAGTGAGTGATCCCGGCGGCGGTCACGAACTCCAGCAGGACCATGTGATCGCGCTTTCCCTGTAGAGCCAGAGCCATTCAGACCCCCCGCCAGCGCTCC
NotI-split sfGFP CUG+1 rev	AGAATTGCGGCCGCGTTTAAACTTAGTGAGTGATCCCGGCGGCGGTCACGAACTCCAGCAGGACCATGTGATCGCGCTTTCCCCCTGTACAGCCACAGCCATTCAGACCCCCCGCCAGCGCTCC
NotI-split sfGFP CUU+1 rev	AGAATTGCGGCCGCGTTTAAACTTAGTGAGTGATCCCGGCGGCGGTCACGAACTCCAGCAGGACCATGTGATCGCGCTTTCCCCCTGTAAAGCCAAAGCCATTCAGACCCCCCGCCAGCGCTCC

NotI-split sfGFP UUA+1 SIINFEKL rev	ACAGCGGCGGCCGCTCAGCTATTTAGAGCTTTTCGAAGTTGATGATGGATTCCAGCTGCTCGAGCCCTGTATAACCATAACCATTCCTCGTTGGGGTCTTTGCTCA
NotI-split sfGFP UUG+1 SIINFEKL rev	ACAGCGGCGGCCGCTCAGCTATTTAGAGCTTTTCGAAGTTGATGATGGATTCCAGCTGCTCGAGCCCTGTACAACCACAACCATTCCTCGTTGGGGTCTTTGCTCA
LARS1 KO#1 sense	CACCGCTGGATCTTCTAAATACCAG
LARS1 KO#1 antisense	AAACCTGGTATTTAGAAGATCCAGC
LARS1 KO#2 sense	CACCGTAGGCATTCCAGTACAGTGC
LARS1 KO#2 antisense	AAACGCACTGTACTGGAATGCCTAC
SLFN11 KO#1 sense	CACCGAGGAGGAGGAGTGATTCGAA
SLFN11 KO#1 antisense	AAACTTCGAATCACTCCTCCTCCTC
SLFN11 KO#2 sense	CACCGTGAGTCCATGGAACGCACAG
SLFN11 KO#2 antisense	AAACCTGTGCGTTCCATGGACTCAC
tRNA-Leu-CAA-6-1 sense	CCGGGGCCCGTCAGGATGGCCGAGCAGTCTTAAGGCGCTGCGTTCAAATCGCACCCTCCGCTGGAGGCGTGGGTTCGAATCCCACTTTTGACAGGTACCTATTGAAAAATTTTTGGAACCCGGGG
tRNA-Leu-CAA-6-1 antisense	AATTCCCCGGGTTCCAAAAATTTTTCAATAGGTACCTGTCAAAAGTGGGATTCGAACCCACGCCTCCAGCGGAGGGTGCGATTTGAACGCAGCGCCTTAAGACTGCTCGGCCATCCTGACGGGCC
tRNA-Leu-TAA-1-1 sense	CCGGGGCCCACCAGGATGGCCGAGTGGTTAAGGCGTTGGACTTAAGATCCAATGGACATATGTCCGCGTGGGTTCGAACCCCACTCCTGGTAGGTACCTATTGAAAAATTTTTGGAACCCGGGG
tRNA-Leu-TAA-1-1 antisense	AATTCCCCGGGTTCCAAAAATTTTTCAATAGGTACCTACCAGGAGTGGGGTTCGAACCCACGCGGACATATGTCCATTGGATCTTAAGTCCAACGCCTTAACCACTCGGCCATCCTGGTGGGCC

SLFN11 ORF	CCTGGTCATCAATGTAGGAGAAGTGACTCTTGGAGAAGAAAACAGAA<br>AAAAGCTGCAGAAAATTCAGAGAGACCAAGAGAAGGAGAGAGTTATG<br>CGGGCTGCATGTGCTTTATTAAACTCAGGAGGAGGAGTGATTCGAATG<br>GCCAAGAAGGTTGAGCATCCCGTGGAGATGGGACTGGATTTAGAACA<br>GTCTTTGAGAGAGCTTATTCAGTCTTCAGATCTGCAGGCTTTCTTTGAG<br>ACCAAGCAACAAGGAAGGTGTTTTTACATTTTTGTTAAATCTTGGAGCA<br>GTGGCCCTTTCCCTGAAGATCGCTCTGTCAAGCCCCGCCTTTGCAGCCT<br>CAGTTCTTCATTATACCGTAGATCTGAGACCTCTGTGCGTTCCATGGAC<br>TCAAGAGAGGCATTCTGTTTCCTGAAGACCAAAAGGAAGCCAAAAATC<br>TTGGAAGAAGGACCTTTTCACAAAATTCACAAGGGTGTATACCAAGAG<br>CTCCCTAACTCGGATCCTGCTGACCCAAACTCGGATCCTGCTGACCTAA<br>TTTTCCAAAAAGACTATCTTGAATATGGTGAAATCCTGCCTTTTCCTGA<br>GTCTCAGTTAGTAGAGTTTAAACAGTTCTCTACAAAACACTTCCAAGAA<br>TATGTAAAAAGGACAATTCCAGAATACGTCCCTGCATTTGCAAACACT<br>GGAGGAGGCTATCTTTTTATTGGAGTGGATGATAAGAGTAGGGAAGT<br>CCTGGGATGTGCAAAAGAAAATGTTGACCCTGACTCTTTGAGAAGGAA<br>AATAGAACAAGCCATATACAAACTACCTTGTGTTCATTTTTGCCAACCC<br>CAACGCCCGATAACCTTCACACTCAAAATTGTGGATGTGTTAAAAAGG<br>GGAGAGCTCTATGGCTATGCTTGCATGATCAGAGTAAATCCCTTCTGCT<br>GTGCAGTGTTCTCAGAAGCTCCCAATTCATGGATAGTGGAGGACAAGT<br>ACGTCTGCAGCCTGACAACCGAGAAATGGGTAGGCATGATGACAGAC<br>ACAGATCCAGATCTTCTACAGTTGTCTGAAGATTTTGAATGTCAGCTGA<br>GTCTATCTAGTGGGCCTCCCCTTAGCAGACCAGTGTACTCCAAGAAAG<br>GCCTGGAACATAAAAAGGAACTCCAGCAACTTTTATTTTCAGTCCCACC<br>AGGATATTTGCGATATACTCCAGAGTCACTCTGGAGGGACCTGATCTC<br>AGAGCACAGAGGACTAGAGGAGTTAATAAATAAGCAAATGCAACCTT<br>TCTTTCGGGGAATTTTGATCTTCTCTAGAAGTTGGGCTGTGGACCTGAA

Supplementary Table S2. Number of mapped reads in Riboseq experiments

Sample	Nr of mapped reads
MDA-MB-231 Ctrl-1	1001227
MDA-MB-231 Ctrl-2	1345554
MDA-MB-231 -Leu-1	1400259
MDA-MB-231 -Leu-2	1678157
MDA-MB-231 tRNA-CAA Ctrl-1	691075
MDA-MB-231 tRNA-CAA Ctrl-2	1084682
MDA-MB-231 tRNA-CAA -Leu-1	1375006
MDA-MB-231 tRNA-CAA -Leu-2	880936
MDA-MB-231 tRNA-UAA Ctrl-1	725032
MDA-MB-231 tRNA-UAA Ctrl-2	1548016
MDA-MB-231 tRNA-UAA -Leu-1	692718
MDA-MB-231 tRNA-UAA -Leu-2	392555
PC3 Ctrl-1	8866491
PC3 -Leu-1	8255142
PC3 Etoposide-1	5506703
PC3 Ctrl-2	1357318
PC3 -Leu-2	5777520
PC3 Etoposide-2	2254625
MD55A3 Ctrl-1	15190586
MD55A3 -Leu-1	11914357

Supplementary Table S3. Primers used for qPCR

Oligo name	Sequence (5' to 3')
tRNA-Leu-CAA-6-1 Fw	GTCAGGATGGCCGAGC
tRNA-Leu-CAA-6-1 Rev	TGTCAAAAGTGGGATTCGAAC
tRNA-Leu-TAA-1-1 Fw	ACCAGGATGGCCGAG
tRNA-Leu-TAA-1-1 Rev	TACCAGGAGTGGGGTTC
tRNA-Leu-CAG-1-7 Fw	TGTCAGGAGTGGGATTC
tRNA-Leu-CAG-1-7 Rev	GTCAGGATGGCCGAG
tRNA-Leu-TAA-3-1 Fw	ACCAGAATGGCCGAG
tRNA-Leu-TAA-3-1 Rev	TACCAGAAGTGGGGTTC
tRNA-Gly-TCC-1-1 Fw	GCGTTGGTGGTATAGTGGTTAGC
tRNA-Gly-TCC-1-1 Rev	TGCGTTGGCCGGGAA
tRNA-Trp-CCA-1-1 Fw	CTCGTGGCGCAACGG
tRNA-Trp-CCA-1-1 Rev	TGACCCCGACGTGATTTG
sfGFP Fw	CCACTACCAGCAGAACAC
sfGFP Rev	CAACGTCCTCGTTGGG
GAPDH Fw	AACTTTGGCATTGTGGAAGG
GAPDH Rev	GGATGCAGGGATGATGTTCT
Adapter set- 5' adapter	/5/rCrCrUrArArGrArGrCrArArGrArArGrArArGrCrCrUrGrGrN
Adapter set- 3' adapter	/5Phos/rGrGrCrUrUrCrUrUrCrUrUrGrCrUrCrUrUrArGrGrArArArArArArArArArAAAA
RT primer set- adapter 1	/5Phos/GGCTTCTTCTTGCTCTTAGGTAGTAGGTTC
RT primer set- adapter 2	GAGGCGAGCGGTCAATTTTCCTAAGAGCAAGAAGAAGCCTTTTTTTTTT

Supplementary Table S4. UUA codon-specific frameshift-derived peptides as identified by mass spectometry

Peptide sequence	GeneID	Ctrl	Ctrl	-Leu	-Leu
LSSWDQAETPGHTPS	SF3B1	0	0	34,3613	33,0876
MGLGHEQGFGAPC	TES	0	0	33,6716	33,7851
SLLGDSAPTLH	AGFG1	0	0	33,161	24,9743
YGDESSNSLPGHSVALC	NCBP1	0	0	32,8458	33,875
QMEVCEVCGAF	LUC7L3	0	0	31,954	30,7175
LAENFCVCH	AK2	0	0	31,5694	30,823
LAEEEDLFDSAHPEEGD	SAFB2	0	0	31,2891	27,7644
SDVNAIIHY	IFIT1	0	0	27,6694	28,1779
IHNANPELTDGQIQAM	ACACA	0	0	26,7905	30,062
VCEVCSAY	LUC7L	0	0	25,7885	24,8415
EHLTQLK	ANKRD30/	0	0	24,7139	24,4247
HITTLQASF	RAB21	0	0	24,2553	34,7668
EINDCIGGTVLK	RSL1D1	0	0	24,0984	23,709
DCQIAHGAAQF	POLR2B	0	0	24,0322	35,276
ANEHQGIGF	UPF1	0	0	23,2287	21,9294
SEDREPEAAEEPER	STX5	0	0	21,8452	21,5325
GSGQAPGSAEGSHER	CPT1C	0	0	21,4683	18,051
AEPEDHYF	ACTR3	0	0	21,1667	22,0838
SHYADVDPENQNFYLNR	SLC38A2	0	0	20,3831	19,963
LNLIHSEISN	MACROH2	0	0	20,0201	28,5203
TVSSSSLPSLR	HELZ	0	0	19,1739	20,1672
DGDVTVTNDGATILK	CCT5	0	0	18,8582	22,1354
RPLPAGPSGDVQEGGQGR	UPF1	0	0	18,6227	20,1632
VLIQVHQASK	TENM1	0	22,0756	19,9275	26,299
ISDLTLGHVNGLQ	CRHBP	0	29,8491	22,4595	22,6551
CGSGPVHISGQH	NPM1	0	30,8217	34,614	31,9329
CGAPAADCAAGPQR	BST2	16,6259	16,63	0	0
AELPSSHYQLQTTSLLER	VKORC1L1	17,7568	18,3811	0	0
AGAPTGAGAGGDSPSR	ZNF524	17,7714	17,5301	0	19,1106
VVLSVSSGGQHTVL	RCC1	18,1612	0	36,6995	37,4104
SPSLSPSPPSPLR	MAP1B	18,6888	19,8365	0	0
LLPCLHSACSAC	TRIM28	19,9019	23,2186	36,4468	29,6952
ASVLSPLF	MTMR8	22,5523	22,2592	23,9316	24,1268
SSFSSFDFLAEDAST	WDR76	23,6732	36,0004	0	0
TLEGIQYDNSIIK	IKBIP	26,7318	24,2571	15,7498	26,5906
ADVIPVHYY	TPP2	27,7637	0	25,5691	21,8894
IISNASCTTNC	GAPDH	28,0177	0	24,0147	37,0131
GIYSSSVY	TEFM	28,0332	26,378	29,6656	24,5097
QLPASEPCCCSGK	PHC3	29,0511	23,2212	26,6965	31,2613
LIIIIVLVVVLLGI	STX3	32,6163	31,1483	29,5012	0
QCLCSAESMVEYGTCMC	SPRY1	32,6424	29,2177	0	0
ISPDQGQQFAQM	CLTC	32,8581	25,7092	0	0

Supplementary Table S5. CUA codon-specific frameshift-derived peptides as identified by mass spectometry

Peptide sequence	GeneID	Ctrl	Ctrl	-Leu	-Leu
HGVTEGLQGQTGPQIYQEK	RPL36	19,5129	19,9735	22,0124	22,2165
GQQGDLQIAAPAHR	BHLHE40	0	0	18,0477	18,8016
EGHGGGHPAAAPPGGAQGAASDH	NUDC	18,8067	19,0334	0	20,2564
EQCGDEASC	CCDC136	20,6994	19,8699	0	0
LQHPVSPSWLQIQPSR	PODXL	20,2062	20,1	0	0
RAQQVVQSQAHQGSEGR	TACO1	18,9669	19,4726	0	0
